# Osteosarcoma Cell-Derived Exosomal ELFN1-AS1 Mediates Macrophage M2 Polarization *via* Sponging miR-138-5p and miR-1291 to Promote the Tumorgenesis of Osteosarcoma

**DOI:** 10.3389/fonc.2022.881022

**Published:** 2022-06-17

**Authors:** Bangmin Wang, Xin Wang, Po Li, Xiaoying Niu, Xiaoxiao Liang, Guancong Liu, Zhiyong Liu, Hong Ge

**Affiliations:** ^1^ Department of Bone Oncology, The Affiliated Cancer Hospital of Zhengzhou University & Henan Cancer Hospital, Zhengzhou, China; ^2^ Department of Radiotherapy, The Affiliated Cancer Hospital of Zhengzhou University & Henan Cancer Hospital, Zhengzhou, China

**Keywords:** tumor microenvironment, tumor-associated macrophages, exosomes, long noncoding RNAs, osteosarcoma

## Abstract

**Background:**

Exosomes play an important role in cell-cell communication by transferring genetic materials such as long non-coding RNAs (lncRNAs) between cancer cells and tumor-associated macrophages (TAMs) in the tumor microenvironment (TME). Recent studies revealed that lncRNA ELFN1-AS1 could function as an oncogene in many human cancers. However, the role of extracellular lncRNA ELFN1-AS1 in cell-to-cell communication of osteosarcoma (OS) has not been fully investigated.

**Methods:**

Functional studies, including CCK-8, EdU staining and transwell assay were performed to investigate the role of ELFN1-AS1 in the progression of OS. 143B *via* xenograft mouse model was established to assess the role of ELFN1-AS1 *in vivo*. In addition, transmission electron microscopy (TEM) and real-time quantitative PCR (RT-qPCR) assay were used to verify the existence of exosomal ELFN1-AS1.

**Results:**

The level of ELFN1-AS1 was markedly upregulated in patients with advanced OS and in OS cells. In addition, overexpression of ELFN1-AS1 significantly promoted the proliferation, migration and invasion of OS cells, while knockdown of ELFN1-AS1 exhibited the opposite effects. Meanwhile, ELFN1-AS1 could be transferred from OS cells to macrophages *via* exosomes. Exosomal ELFN1-AS1 from 143B cells was able to promote macrophage M2 polarization, and M2 macrophage in return facilitated OS progression. Mechanistically, overexpression of ELFN1-AS1 upregulated CREB1 level *via* sponging miR-138-5p and miR-1291 in macrophage *via*.

**Conclusion:**

OS cell-derived exosomal ELFN1-AS1 was able to induce macrophage M2 polarization *via* sponging miR-138-5p and miR-1291, and M2 macrophage notably facilitated the progression of OS. These data suggested that ELFN1-AS1 might serve as a potential therapeutic target for osteosarcoma.

## Introduction

Osteosarcoma (OS) is a musculoskeletal malignancy that occurs mostly in children and adolescents, which is characterized by a high risk of metastatic progression in humans (1–4). Recently, clinical treatments for patients with OS typically include immunotherapy, chemotherapy, surgery and radiotherapy (5, 6). However, the 5-year survival rate of patients with metastatic or recurrent OS is about 20% (7). Therefore, identification of promising therapeutic molecular targets of OS may help to improve the treatment of patients.

Long noncoding RNAs (lncRNAs) are a class of noncoding RNAs longer than 200 nucleotides (8). LncRNAs have been found to regulate transcriptional or posttranscriptional gene expression by acting as sponges of microRNA (miRNA) (9). In addition, lncRNAs function as tumor suppressors or oncogenes in many cancers, including OS (1, 10–12). Previous study showed that lncRNA SNHG3 was able to promote the migration and invasion of OS cells by sponging miR-151a-3p (10). Wang et al. found that lncRNA GAS5 inhibited the migration and invasion of OS cells *via* sponging miR-203a (13). Recently, ELFN1-AS1 has been found to act as an oncogene in many human cancers (14–16). Zhang et al. reported that the expression of ELFN1-AS1 was increased in esophageal cancer tissues; downregulation of ELFN1-AS1 inhibited the proliferation, migration and invasion of esophageal cancer cells *via* sponging miR-183-3p (14). Additionally, du et al. illustrated that ELFN1-AS1 could promote the proliferation and invasion of colorectal cancer cells *via* sponging miR-191-5p (17). However, the role of ELFN1-AS1 in OS has not yet been explored.

Tumor microenvironment (TME) is a complex system containing multiple cells, which exert important roles in the sustained growth and invasion of tumor cells (18, 19). Tumor-associated macrophages (TAMs), important immune cells, are the major component of the TME (20). It has been reported that TAMs can be polarized into either a pro−tumor (M2 macrophages) or an anti−tumor phenotype (M1 macrophages) (21). M2-polarized TAMs have been shown to contribute to tumor progression and metastasis (22, 23). Nevertheless, the relationship between ELFN1-AS1 and macrophage M2 polarization remains largely unknown.

Exosomes (30-200 nm in diameter) containing a variety of bioactive molecules such as miRNAs, lncRNAs and proteins can serve as communication tools between cells (24). In addition, exosomes contribute to the modulation of TME through intercellular communication (25). Liang et al. found that tumor cell-derived exosomal lncRNA RPPH1 induced macrophage M2 polarization to promote colorectal cancer metastasis (26). However, the interaction among OS-derived exosomes, ELFN1-AS1 and macrophage M2 polarization remains unclear.

In the current study, we found that the level of ELFN1-AS1 was markedly upregulated in OS tissues. In addition, we found that 143B cell-derived exosomal ELFN1-AS1 could induce macrophages M2 polarization *via* sponging miR-138-5p and miR-1291, and M2 macrophages in return promoted OS progression. These data suggested that exosomal ELFN1-AS1 may serve as a therapeutic target for OS.

## Materials and Methods

### Data Collection and Differential Expression Analysis

The lncRNAs expression data of patients with OS were downloaded from the cancer genome atlas (TCGA) and TARGET datasets. LASSO Cox regression model analysis was applied to screen the differentially expressed lncRNAs (DElncRNAs) between patients with OS. In addition, TCGA dataset was used to determine the association between the overall survival of patients with OS and ELFN1-AS1 level.

### Patient Samples

OS tissues and matched normal tissues were obtained from 10 patients with OS who were admitted to the Affiliated Cancer Hospital of Zhengzhou University. In addition, a total of 20 serum samples were collected from 10 patients with OS and 10 healthy donors from the Affiliated Cancer Hospital of Zhengzhou University. The ethical approval was approved by the ethics committee of the Affiliated Cancer Hospital of Zhengzhou University. Written informed consent was obtained from all participators.

### Cell Culture

Three human OS cell lines (143B, MG63 and SW1353) and normal human osteoblasts cell line (hFOB) and THP-1 cell line were obtained from the Type Culture Collection of the Chinese Academy of Sciences (Shanghai, China). Cells were maintained in DMEM (Thermo Fisher Scientific, Waltham, MA, USA) medium supplemented with 10% fetal bovine serum (FBS; Thermo Fisher Scientific) at 37°C under 5% CO_2_.

### Real-Time Quantitative PCR (RT-qPCR)

RT-qPCR assay was used to determine the gene level of ELFN1-AS1 and CREB1 in OS tissues and in cells. The TRIzol reagent (Thermo Fisher Scientific) was used for the extraction of total RNA. Reverse transcription was conducted using an EntiLink™ 1st Strand cDNA Synthesis Kit (ELK Biotechnology, Wuhan, China) to synthesize the first chain of cDNA. After that, qPCR was carried out using EnTurbo™ SYBR Green PCR SuperMix (ELK Biotechnology) according to the manufacturer’s instructions. β-actin was used as an internal control for ELFN1-AS1, CREB1. U6 was used as an internal control for miR-138-5p. The method of 2^−ΔΔct^ was used for the calculation of qPCR results.

### Fluorescence *In Situ* Hybridization (FISH)

FISH assay was performed to measure the level of ELFN1-AS1 in OS tissues and matched normal tissues. Tumor tissues were fixed with 10% formaldehyde, embedded in paraffin and then cut into 5-μm thick slices. After that, the slices were deparaffinized and rehydrated. Later on, the level of ELFN1-AS1 in tumor tissues was detected using biotin-labeled ELFN1-AS1 probes (BOSTER, Wuhan, China), as described previously (27). In addition, cellular localization of ELFN1-AS1 and miR-138-5p and miR-1291 in macrophages were analyzed using FISH assay. The fluorescence-conjugated ELFN1-AS1, miR-138-5p or miR-1291 probes were obtained from RiboBio (Guangzhou, China). Then, hybridizations were performed using a FISH kit (RiboBio) according to the manufacturer’s instructions. Subsequently, the images of FISH were captured using a fluorescence microscope (Leica CTR5000; Buffalo Grove, IL, USA).

### Cell Transfection

ELFN1-AS1 siRNA 1 and ELFN1-AS1 siRNA2 and siRNA control were purchased from GenePharma (Shanghai, China). In addition, ELFN1-AS1 was synthesized and cloned into pcDNA3.1 vector. These plasmids were transfected into 143B and MG63 cells using Lipofectamine 3000 (Thermo Fisher Scientific) according to the manufacturer’s instructions.

### Cell Counting Kit-8 (CCK-8) Assay

CCK-8 assay was used to measure cell viability. 143B or MG63 cells (5.0 × 10^3^ cells/well) were plated onto 96-well plates and then incubated for indicated time points. After that, the cells were treated with 10 μL of CCK-8 (Dojindo, Kyushu, Japan) reagent, and cells were then incubated for another 2 h. Next, the absorbance at 450 nm was recorded using a microplate reader at different time points (0, 24, 48 and 72 h).

### EdU Staining Assay

The Cell-Light EdU Apollo567 *In Vitro* Kit (RiboBio) was used to determine cell proliferation ability according to the manufacturer’s instructions. Briefly, cells were incubated with EdU reagent for 2 h, fixed in 4% paraformaldehyde and then stained with apollo dye solution. Later on, the EdU-positive cells were observed using a fluorescence microscope. EdU-positive cells in three random fields were counted.

### Flow Cytometry Assay

The fluorescein isothiocyanate (FITC) Annexin V Apoptosis Detection Kit (BD Biosciences, Franklin Lake, NJ, USA) was used to detect apoptosis according to the manufacturer’s instructions. In addition, the number of early apoptotic cells (Annexin V positive/PI negative) and late apoptotic/necrotic cells (Annexin V positive/PI positive) was measured using a flow cytometer (BD Biosciences). The cell apoptosis was analyzed using with BD CellQuest™ Pro software (version 5.1).

### Transwell Assays

Cell migration and invasion was detected using transwell chamber (Corning, New York, NY, USA). Briefly, OS cells resuspended in 200 μL serum-free DMEM medium were placed on the upper chamber. Meanwhile, DMEM medium containing 12% FBS was added into the lower chamber. After 24 h of incubation at 37°C, cells on the lower membrane surface were stained with 0.2% crystal violet solution. After that, the migrated or invaded cells were observed using a light microscope. The migrated or invaded cells in three random fields were counted. Regarding as cell invasion, transwell chamber with pre-coated 100 μL Matrigel (BD Biosciences) was used.

### Western Blot Assay

The concentration of total protein was assessed with a BCA Protein assay kit (Beyotime, Shanghai, China). Later on, proteins were separated by 10% sodium dodecyl sulfate-polyacrylamide gel electrophoresis and then transferred onto polyvinylidene difluoride (PVDF) membrane. Following blocking in 5% skimmed milk in TBST for 1 h, the membrane was incubated with primary antibodies at 4°C overnight. Next, the membrane was probed with corresponding secondary antibody for 1 h. Subsequently, the membrane was treated with an electrochemiluminescence reagent (Thermo Fisher Scientific) and then exposed to x-ray films for chemiluminescence. The antibodies for vimentin, N-cadherin, E-cadherin, CREB1, CD206, β-actin (Abcam, Cambridge, MA, USA) were used for blotting. The intensity of blots was quantified using ImageJ software.

### Exosome Isolation and Characterization

The cell lines were cultured in a DMEM complete medium until about 80% confluent at 37°C under 5% CO_2_. Then, cells were replaced with DMEM medium without FBS; the exosomes in OS conditioned medium (CM) were isolated using ultracentrifugation methods as described previously (26). Nanoparticle Tracking Analysis (NTA) with a ZetaView nanoparticle tracking analyzer instrument (Particle Metrix) was applied to measure the size distribution of exosomes. In addition, the expression of exosomal specific surface biomarkers CD81 and TSG101 were detected with western blot assay.

The exosomes were resuspended and fixed in 2.5% glutaraldehyde and then absorbed onto a copper grid. Later on, 1% phosphotungstic acid was used to stain exosomes for 3 min. Next, morphologies of the samples were captured using a transmission electron microscope (TEM, Hitachi HT7700).

### Exosome Labeling and Uptake

Exosomes were labeled with PKH26 dye (Sigma Aldrich, St. Louis, MO, USA). Later on, macrophages were incubated with the labeled exosomes for 48 h. Then, a fluorescence microscope was applied to observe the internalization of exosomes by macrophages. Nucleus was counterstained with DAPI.

### Co-Culture System

OS cells transfected with Cy3 labeled-ELFN1-AS1 (RiboBio) were cultured in the Transwell^®^ polyester permeable supports. Meanwhile, macrophages were incubated in the lower chamber. After that, OS cells were co-cultured with macrophages for 24 h. Subsequently, images were captured using a fluorescence microscope.

### Dual-Luciferase Reporter Assay

The wild-type (WT) or mutant (Mut) miRNA binding sites of ELFN1-AS1 or CREB1 were separately inserted into the pMir-GLO-based luciferase reporter vector (Promega). Then, macrophages were co-transfected with luciferase reporter vector and miR-138-5p mimics. After that, the luciferase activity was detected at 48 h using the Dual Luciferase Reporter Assay System (Beyotime).

### *In Situ* RNA Pull-Down Assay

Pierce Magnetic RNA-Protein Pull-Down Kit (Thermo Fisher Scientific) was used for RNA pull-down assay according to the manufacturer’s instructions. Briefly, the extracts from OS cells were incubated with biotin-labeled ELFN1-AS1 probes and magnetic beads. After that, the retrieved RNA was measured by RT-qPCR.

### Animal Study

BALB/c nude mice (4-5 weeks old) were purchased from the Vital River Laboratories (Beijing, China). Animals were divided randomly into four groups: 143B cell, 143B cell + Macrophages (143B + M), MG-63 cell + exosomes-treated macrophages (143B + M-Exo) and 143B + M-Exo-ELFN1-AS1 siRNA1 groups. 143B cells plus indicated macrophages were subcutaneously injected into the left flank of nude mice. The volume was calculated by the following formula V = (length x width^2^)/2. The mice were sacrificed on day 21, and the tumors were collected. In addition, cell apoptosis in tumor tissues was measured using the TUNEL Assay Kit (Roche) according to the manufacturer’s instructions. All animal experiments were approved by the Ethics Committee of the Affiliated Cancer Hospital of Zhengzhou University and performed following the recommended procedures of National Institutes of Health guide for the care and use of laboratory animals.

### Statistical Analysis

The non-parametric analysis was used for the statistical analysis of this study. Data are presented as the mean ± standard deviation (S.D.). Differences between tumor tissues and adjacent normal tissues were analyzed using paired Student’s t-test. Differences between OS patients and healthy controls were analyzed by un-paired Student’s t-test. Differences between three or more groups were analyzed by One-way analysis of variance (ANOVA) and Tukey’s tests. P-value<0.05 was considered as statistically significant. Software Gradpad Prism was used to perform these analyses. All data were repeated independently at least three times.

## Results

### The Level of ELFN1-AS1 Is Elevated in OS Tissues and OS Cells

**Figure 1:**
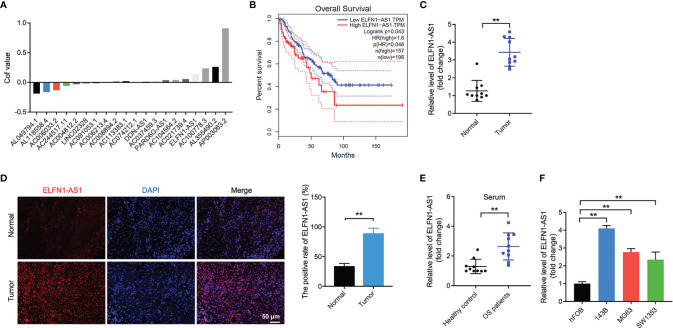
ELFN1-AS1 levels are elevated in OS tissues and OS cells. **(A)** In the TARGET dataset, 8 downregulated DElncRNAs, and 12 upregulated DElncRNAs from OS tissues and corresponding normal controls were identified using LASSO Cox regression analysis. Volcano plot of DElncRNAs in TARGET dataset. **(B)** The correlation between the level of ELFN1-AS1 and overall survival rate of patients with OS in the TCGA dataset. **(C)** ELFN1-AS1 level in OS tissues and matched normal sample tissues were measured using RT-qPCR (n = 10). The data were statistically analyzed using Student’s t-test. **(D)** FISH staining analysis of ELFN1-AS1 level in OS tissues and matched normal tissues. **(E)** ELFN1-AS1 level in serum samples from patients with OS and healthy controls were measured using RT-qPCR (n = 10). The data were statistically analyzed using Student’s t-test. **(F)** RT-qPCR analysis of ELFN1-AS1 level in MG63 and SW1353 cells (n = 3). The significance between groups was analyzed by one-way ANOVA. **P < 0.01.

With the aim of screening the DElncRNAs in patents with OS, TARGET dataset was used and DElncRNAs were analyzed using LASSO Cox regression analysis. As shown in **Figure 1A**, a total of 20 DElncRNAs were identified in TARGET dataset. Among these DElncRNAs, ELFN1-AS1 was reported to be obviously upregulated in human cancers including colorectal cancer, ovarian cancer and esophageal cancer (14–16). Meanwhile, the data in TCGA dataset showed that high level of ELFN1-AS1 was negatively associated with the overall survival of patients with OS (**Figure 1B**). In addition, ELFN1-AS1 expression was notably upregulated in OS tissues compared with that in normal tissues (**Figures 1C, D**); ELFN1-AS1 level in serum samples was higher in patients with OS compared with that in healthy donors (**Figure 1E**). Moreover, the analysis of the TCGA dataset indicated that the average level of ELFN1-AS1 in sarcoma (SARC) tissues were higher than that of in normal tissues (**Supplementary Figure 1**). Furthermore, ELFN1-AS1 levels were markedly increased in 143B, MG63 and SW1353 cells compared with that in hFOB cells (**Figure 1F**). Collectively, ELFN1-AS1 is elevated in OS and high level of ELFN1-AS1 was associated with a poor overall survival of patients.

### ELFN1-AS1 Promotes the Proliferation, Migration and Invasion of OS Cells

**Figure 2:**
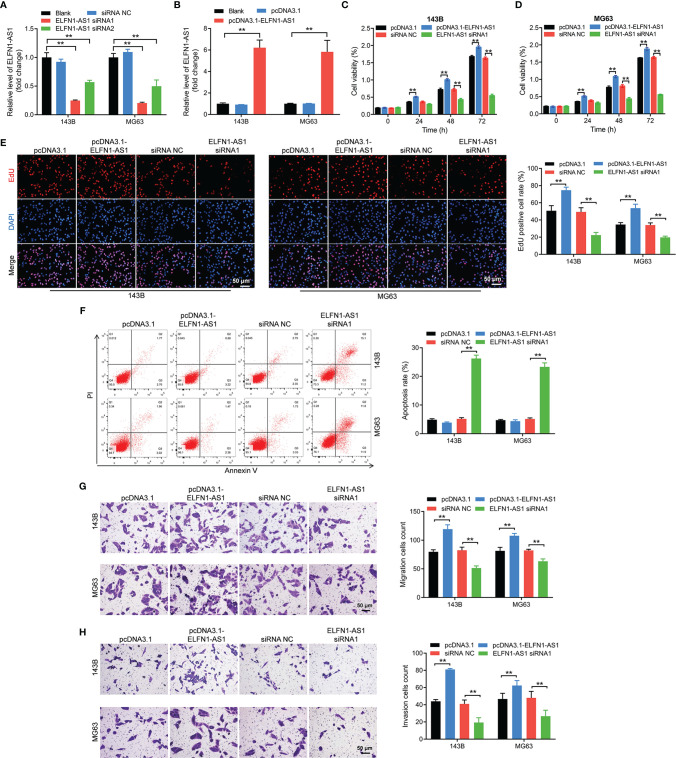
ELFN1-AS1 promoted the proliferation, migration and invasion of OS cells. **(A)** RT-qPCR analysis of ELFN1-AS1 level in 143B and MG63 cells transfected with ELFN1-AS1 siRNA1 or ELFN1-AS1 siRNA2 (n = 3). **(B)** RT-qPCR analysis of ELFN1-AS1 level in 143B and MG63 cells transfected with pcDNA3.1 ELFN1-AS1 (n = 3). **(C, D)** 143B and MG63 cells were transfected with pcDNA3.1 ELFN1-AS1 or ELFN1-AS1 siRNA1. CCK-8 assay was applied to measure cell viability (n = 3). **(E)** EdU staining assay was applied to detect cell proliferation (n = 3). **(F)** Flow cytometry assay was applied to determine cell apoptosis. **(G)** Transwell migration and **(H)** invasion assays were applied to determine cell migration and invasion (n = 3). The significance between groups was analyzed by one-way ANOVA. **P < 0.01.

To investigate the effect of ELFN1-AS1 on OS cells, we knockdown the ELFN1-AS1 level in OS cells (143B and MG63 cells) with siRNAs. As shown in **Figure 2A**, ELFN1-AS1 siRNA1 remarkably reduced ELFN1-AS1 level in OS cells. In addition, the expression of ELFN1-AS1 in OS cells was significantly elevated by ELFN1-AS1-overexpressing plasmid (pcDNA3.1-ELFN1-AS1) (**Figure 2B**). Additionally, overexpression of ELFN1-AS1 markedly increased the viability, proliferation, migration and invasion abilities of OS cells; in contrast, knockdown of ELFN1-AS1 exhibited opposite effects (**Figures 2C–H**). To sum up, ELFN1-AS1 was able to promote the proliferation, migration and invasion of OS cells.

### ELFN1-AS1 Can Be Transferred From 143B Cells to Macrophages *via* Exosomes

**Figure 3:**
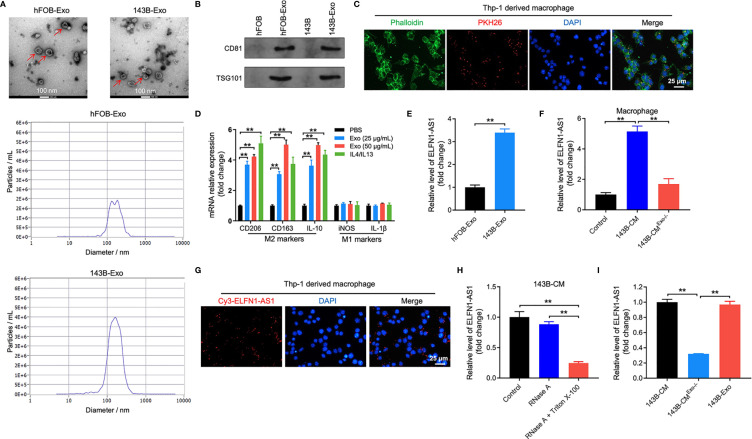
ELFN1-AS1 could be delivered to macrophages by exosomes. **(A)** Exosomes were isolated from the hFOB and 143B cell CM. The size of exosomes was assessed by NTA assay. The morphology of exosomes was observed by TEM at 100 keV. Red arrow, exosomes. **(B)** The expression of exosome surface markers CD81 and TSG101 were determined by western blot assay (n = 3). **(C)** PKH26-labeled exosomes from 143B cells absorbed by macrophages were observed under fluorescence microscope (n = 3). **(D)** RT-qPCR analysis of CD206, CD163, IL-10, iNOS, IL-1β in macrophages treated with indicated exosomes or IL4/IL13 (n = 3). **(E)** ELFN1-AS1 level in exosomes isolated from CM of hFOB and 143B cells were detected using RT-qPCR (n = 3). **(F)** RT-qPCR analysis of ELFN1-AS1 level in macrophages incubated with CM, 143B cell-CM, exosome-depleted 143B cell-CM (n = 3). **(G)** Cy3-tagged ELFN1-AS1 labeled 143B cells were co-cultured with macrophages for 48 h. Meanwhile, the fluorescence signal in macrophages was observed by microscopy (n = 3). **(H)** RT-qPCR analysis of ELFN1-AS1 level in the CM of 143B cells (n = 3). **(I)** RT-qPCR analysis of ELFN1-AS1 level in 143B cell-Exo, 143B cell-CM, exosome-depleted 143B cell-CM (n = 3). The significance between two groups was analyzed by Student’s t test or one-way ANOVA respectively. **P < 0.01.

It has been reported that cancer cell-derived exosomes could induce macrophage M2 polarization, which in turn promoted the progression of cancer (28). Thus, to investigate interaction between OS-derived exosomes and macrophages (PMA-treated THP-1 monocytes) M2 polarization *via*, exosomes were collected from mediums of OS cells. As indicated in **Figure 3A**, the shape of isolated vesicles was round, cup-shaped and the diameter of vesicles ranged from 50 to 150 nm. In addition, these vesicles expressed exosomal specific markers CD81 and TSG101 (**Figure 3B**). All these data indicated that the isolated vesicles were exosomes.

To further assess whether 143B cell-derived exosomes could be internalized by macrophages, macrophages were incubated with PKH26-labeled exosomes. After 48 h of incubation, PKH26 fluorescence dye could be observed in macrophages, suggesting that 143B cell-derived exosomes was absorbed by macrophages (**Figure 3C**).

Next, we found that 143B cell-derived exosomes notably upregulated the level of CD206, CD163 and IL-10 (M2 surface markers) in macrophages; whereas 143B cell-derived exosomes had few effect on the expression of iNOS and IL-1β (M1 surface markers) (**Figure 3D**). Meanwhile, the level of ELFN1-AS1, AC100778.3, AL355490.2 and AC021739.4 were upregulated in 143B cell-derived exosomes (**Figure 3E** and **Supplementary Figure 2**). Importantly, macrophages cultured in 143B cell-CM expressed a higher level of ELFN1-AS1; whereas, this increase was abolished when exosomes were depleted (**Figure 3F**). In addition, 143B cells transfected with Cy3 fluorescence dye-labeled ELFN1-AS1 were co-cultured with macrophages, and Cy3 fluorescence dye was observed in macrophages (**Figure 3G**). This data suggested 143B cell-derived exosomal ELFN1-AS1 could be absorbed by macrophages. Moreover, RNase A had few effect on ELFN1-AS1 level in 143B cell-CM, whereas RNase A combined with Triton X-100 treatment remarkably decreased the level of ELFN1-AS1 in 143B cell-CM, indicating that extracellular ELFN1-AS1 was encased within membrane (**Figure 3H**). Meanwhile, the level of ELFN1-AS1 was almost equal in 143B cell-CM and 143B cell-Exo (**Figure 3I**). Collectively, ELFN1-AS1 was able to be transferred from 143B cells to macrophages *via* exosomes.

### Exosomal ELFN1-AS1 Promotes OS Cell Migration and Invasion by Inducing Macrophages M2 Polarization

**Figure 4:**
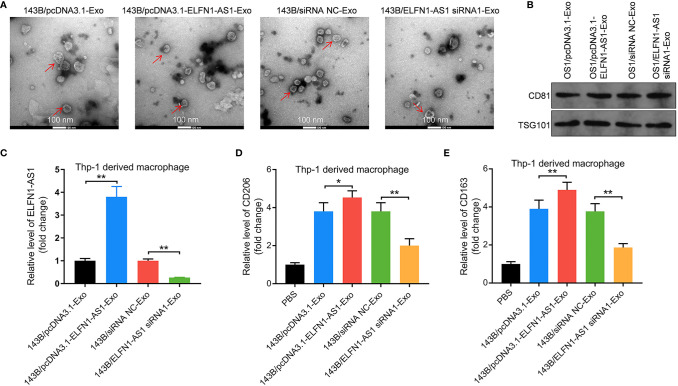
Exosomes with upregulated ELFN1-AS1 promoted macrophages M2 polarization. **(A)** Exosomes were isolated from the CM of 143B cells transfected with pcDNA3.1 ELFN1-AS1 (143B/pcDNA3.1-ELFN1-AS1-Exo) or ELFN1-AS1 siRNA1 (143B/ELFN1-AS1 siRNA1-Exo). The size of exosomes was assessed by NTA assay. The morphology of exosomes was observed by TEM at 100 keV. Red arrow, exosomes. **(B)** The expression of exosome surface markers CD81 and TSG101 were detected with western blot assay (n = 3). **(C)** RT-qPCR analysis of ELFN1-AS1 level in macrophages (n = 3). **(D, E)** RT-qPCR analysis of CD206 and CD163 level in macrophages (n = 3). The significance between groups was analyzed by one-way ANOVA. *P < 0.05; **P < 0.01.

To explore the effect of exosomal ELFN1-AS1 on macrophage polarization, exosomes were isolated from the CM of 143B cells. The result of TEM showed that the isolated vesicles were round and cup-shaped particles (**Figure 4A**). Meanwhile, the vesicles highly expressed CD81 and TSG101, suggesting that the vesicles were exosomes (**Figure 4B**). Additionally, 143B/pcDNA3.1-ELFN1-AS1-Exo significantly increased the level of ELFN1-AS1, CD206 and CD163 were in macrophages, while 143B/ELFN1-AS1 siRNA1-Exo exhibited the opposite effects (**Figures 4C–E**). These data suggested that exosomal ELFN1-AS1 could promote macrophages M2 polarization.

**Figure 5:**
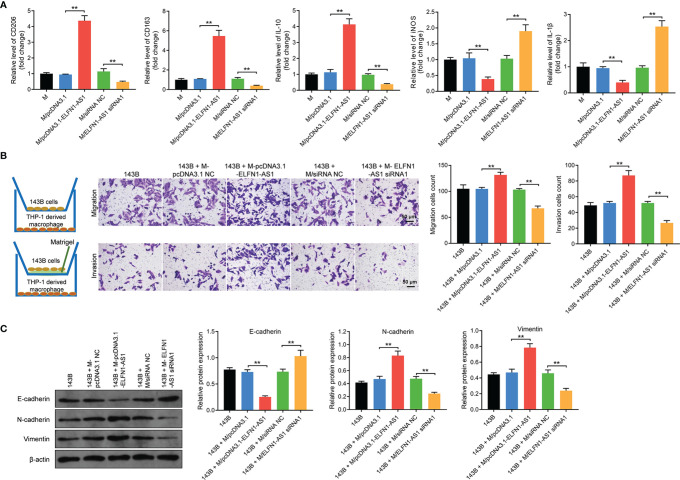
Exosomes with upregulated ELFN1-AS1 promoted migration and invasion in OS cells *via* inducing macrophages M2 polarization *in vitro*. **(A)** Macrophages were transfected with pcDNA3.1 ELFN1-AS1 or ELFN1-AS1 siRNA1; RT-qPCR analysis of CD206, CD163, IL-10, iNOS, IL-1β level (n = 3). **(B)** 143B cells (upper chamber) were co-cultured with transfected macrophages (lower chamber). Transwell migration and invasion assays were applied to determine cell migration and invasion (n = 3). **(C)** Western blot analysis of E-cadherin, N-cadherin and vimentin in 143B cells (n = 3). The significance between groups was analyzed by one-way ANOVA. **P < 0.01.

Next, macrophages were transfected with pcDNA3.1-ELFN1-AS1 or ELFN1-AS1 siRNA1. The result of RT-qPCR indicated overexpression of ELFN1-AS1 dramatically upregulated the level of CD206, CD163 and IL-10 and downregulated the level of iNOS and IL-1β in macrophages, whereas ELFN1-AS1 siRNA1 displayed the opposite effects (**Figure 5A**). Moreover, the migration and invasion abilities of 143B cells were remarkably increased when co-cultured with pcDNA3.1 ELFN1-AS1 transfected macrophages (**Figure 5B**). Consistently, macrophages overexpressing ELFN1-AS1 significantly downregulated the level of E-cadherin and upregulated the expression of vimentin and N-cadherin in 143B cells (**Figure 5C**). Collectively, exosomal ELFN1-AS1 promoted the migration and invasion OS cell by inducing macrophages M2 polarization.

### Exosomal ELFN1-AS1 Promotes Macrophage M2 Polarization *via* Regulating miR-138-5p/CREB1 Axis

**Figure 6:**
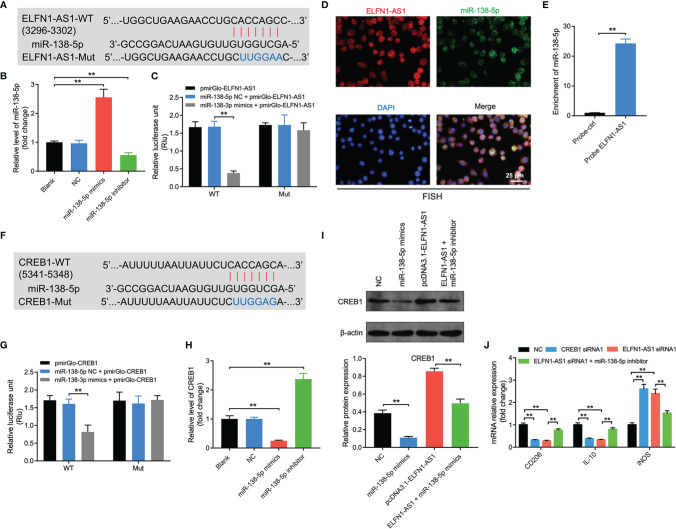
Exosomal ELFN1-AS1 promoted macrophage M2 polarization *via* regulation of miR-138-5p/CREB1 axis. **(A)** Schematic diagram of binding sites between ELFN1-AS1 and miR-138-5p. **(B)** RT-qPCR analysis of miR-138-5p level in macrophages after transfecting with miR-138-5p mimics or miR-138-5p inhibitor (n = 3). **(C)** Luciferase activity in macrophages treated with the ELFN1-AS1-wt/mut plasmids together with miR-138-5p mimics was analyzed (n = 3). **(D)** FISH detection of ELFN1-AS1 and miR-138-5p in macrophages. **(E)** RNA pull-down assays were used to determine the interaction between ELFN1-AS1 and miR-138-5p (n = 3). **(F)** Schematic diagram of binding sites between CREB1 and miR-138-5p. **(G)** Luciferase activity in macrophages treated with the CREB1-wt/mut plasmids together with miR-138-5p mimics was analyzed (n = 3). **(H)** RT-qPCR analysis of CREB1 level in transfected macrophages. **(I)** Western blot analysis of CREB1 expression in transfected macrophages (n = 3). **(J)** RT-qPCR analysis of CD206, IL-10, iNOS level in transfected macrophages (n = 3). The significance between two or more groups was analyzed by Student’s t test or one-way ANOVA respectively. **P < 0.01.

Evidence has shown that lncRNAs may regulate the expression of mRNA indirectly by acting as miRNA sponges (29). Thereby, the potential targets of ELFN1-AS1 were explored by using online tool Starbase (https://starbase.sysu.edu.cn/). As revealed in **Figure 6A**, a binding site was found between ELFN1-AS1 and miR-138-5p. In addition, miR-138-5p mimics markedly increased the level of miR-138-5p in macrophages; whereas, miR-138-5p inhibitor exerted the opposite effects (**Figure 6B**). Meanwhile, the luciferase activity was lower in macrophages co-transfected with ELFN1-AS1‐WT and miR-138-5p mimics (**Figure 6C**). FISH assay showed that ELFN1-AS1 and miR-138-5p were partially co-localized in the cytoplasm (**Figure 6D**). Furthermore, miR-138-5p could be pulled down by biotin-labeled ELFN1-AS1 probe (**Figure 6E**). All these data suggested that ELFN1-AS1 could function as a competing endogenous RNA (ceRNA) of miR-138-5p in OS.

Next, the data from TargetScan database (https://www.targetscan.org/vert_72/) suggested that CREB1 might be a potential target of miR-138-5p (**Figure 6F**). In addition, the luciferase activity was notably lower in macrophages co-transfected with CREB1‐WT and miR-138-5p mimics (**Figure 6G**). Moreover, miR-138-5p mimics markedly downregulated the mRNA and protein expression of CREB1 in macrophages (**Figures 6H, I**). Conversely, overexpression of ELFN1-AS1 significantly upregulated the expression of CREB1 in macrophages; however, that upregulation was reversed by miR-138-5p mimics (**Figure 6I**).

Furthermore, CREB1 siRNA1 decreased the expression of CREB1 in macrophages (**Supplementary Figures 3A, B**). Notably, either ELFN1-AS1 siRNA1 or CREB1 siRNA1 was able to decrease the level of CD206 and IL-10 and increase iNOS expression in macrophages (**Figure 6J**). However, the effects of ELFN1-AS1 siRNA1 on these genes were reversed by miR-138-5p inhibitor (**Figure 6J**). To sum up, exosomal ELFN1-AS1 promoted macrophage M2 polarization *via* regulating miR-138-5p/CREB1 axis.

### Exosomal ELFN1-AS1 Promoted Macrophage M2 Polarization *via* Regulating miR-1291/CREB1 Axis

**Figure 7:**
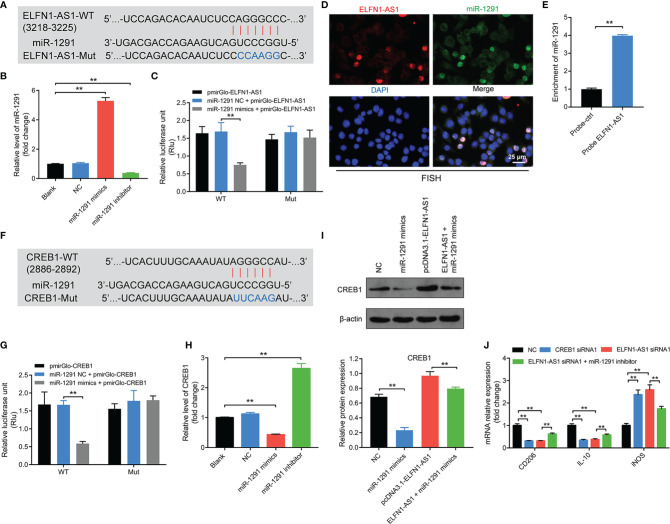
Exosomal ELFN1-AS1 promoted macrophage M2 polarization *via* regulating miR-1291/CREB1 axis. **(A)** Schematic diagram of binding sites between ELFN1-AS1 and miR-1291. **(B)** RT-qPCR analysis of miR-1291 level in macrophages after transfecting with miR-1291 mimics and miR-1291 inhibitor (n = 3). **(C)** Luciferase activity in macrophages treated with the ELFN1-AS1-wt/mut plasmids together with miR-1291 mimics was analyzed (n = 3). **(D)** FISH detection of ELFN1-AS1 and miR-1291 in macrophages. **(E)** RNA pull-down assays were used to determine the interaction between ELFN1-AS1 and miR-1291 (n = 3). **(F)** Schematic diagram of binding sites between CREB1 and miR-1291. **(G)** Luciferase activity in macrophages treated with the CREB1-wt/mut plasmids together with miR-1291 mimics was analyzed (n = 3). **(H)** RT-qPCR analysis of CREB1 level in transfected macrophages (n = 3). **(I)** Western blot analysis of CREB1 expression in transfected macrophages (n = 3). **(J)** RT-qPCR analysis of CD206, IL-10, iNOS level in transfected macrophages (n = 3). The significance between two or more groups was analyzed by Student’s t test or one-way ANOVA respectively. **P < 0.01.

Since lncRNAs may act as a ceRNA to sponge various miRNAs (29, 30), the other potential targets of ELFN1-AS1 were explored as well. Base on the analysis of Starbase, a binding site was also found between ELFN1-AS1 and (**Figure 7A**). In addition, miR-1291 mimics markedly increased the level of miR-1291 in macrophages, whereas miR-1291 inhibitor displayed the opposite effects (**Figure 7B**). Moreover, the luciferase activity was lower in macrophages co-transfected with ELFN1-AS1‐WT and miR-1291 mimics (**Figure 7C**). FISH assay showed that ELFN1-AS1 and miR-1291 were partially co-localized in the cytoplasm (**Figure 7D**). Meanwhile, miR-1291 was pulled down by biotin-labeled ELFN1-AS1 probe (**Figure 7E**). These data suggested that ELFN1-AS1 could function as a ceRNA of miR-1291 in OS.

Next, the data from TargetScan database showed that CREB1 might be a potential target of miR-1291 as well (**Figure 7F**). Additionally, the luciferase activity was notably lower in macrophages co-transfected with CREB1‐WT and miR-1291 mimics (**Figure 7G**). Meanwhile, miR-1291 mimics markedly downregulated mRNA and protein expression of CREB1 in macrophages (**Figures 7H, I**). Similarly, ELFN1-AS1-induced upregulation of CREB1 in macrophages was reversed by miR-138-5p mimics (**Figure 7I**). Importantly, the effects of ELFN1-AS1 siRNA1 on the expression of CD206, IL-10 and iNOS in macrophages were reversed by miR-1291 inhibitor (**Figure 7J**). To sum up, exosomal ELFN1-AS1 promoted macrophage M2 polarization *via* sponging miR-1291, and then activating CREB1.

In additional to miR-1291, a binding site was also found between ELFN1-AS1 and miR-663b (**Supplementary Figure 4A**). In addition, miR-663b mimics markedly upregulated the level of miR-663b in macrophages (**Supplementary Figure 4B**). Meanwhile, luciferase reporter assays validated the binding potential between ELFN1-AS1 and miR-663b in OS (**Supplementary Figure 4C**). Subsequently, the data from TargetScan database suggested that CREB1 might be a potential target of miR-663b, and the interaction between CREB1 and miR-663b was verified by the dual luciferase reporter assay (**Supplementary Figures 4D, E**). Moreover, miR-663 mimics remarkably reduced mRNA expression of CREB1 in macrophages (**Supplementary Figure 4F**).

### Exosomes With ELFN1-AS1 Promotes Tumor Growth *via* Inducing Macrophage M2 Polarization *In Vivo*

**Figure 8:**
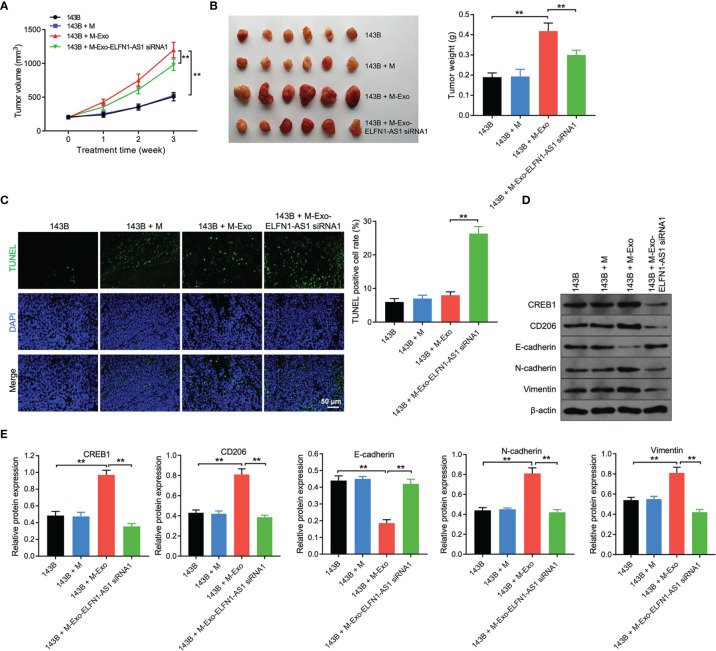
Exosomes with ELFN1-AS1 promotes tumor growth *via* inducing macrophage M2 polarization *in vivo. via in vivo* **(A)** The volume of nude mice was monitored weekly (n = 6). **(B)** Xenograft tumors were collected, and tumor weights were calculated (n = 6). **(C)** Cell apoptosis in tumor tissues were detected using TUNEL assay (n = 3). **(D, E)** Western blot assay analysis of CREB1, CD206, E-cadherin, N-cadherin, and vimentin protein expression in tumor tissues (n = 3). The significance between four groups was analyzed by Student’s t test or one-way ANOVA respectively. **P < 0.01.

We next investigated the role of exosomal ELFN1-AS1 in OS *in vivo*, 143B cells and macrophages were subcutaneously injected into nude mice. As revealed in **Figures 8A, B**, macrophages treated with 143B cell-Exo significantly increased the volume and weight of tumors compared with the control group; however, tumor-promoted effect was impaired when macrophages treated with 143B/ELFN1-AS1 siRNA1-Exo. Moreover, exosomes containing ELFN1-AS1 siRNA1 significantly promoted cell apoptosis in tumor tissues compared with 143B + M^Exo^ group (**Figure 8C**). Meanwhile, exosomes containing ELFN1-AS1 siRNA1 reduced the expression of CREB1, CD206, vimentin and N-cadherin, and increased the level of E-cadherin in tumor tissues, compared with the 143B + M^Exo^ group (**Figures 8D, E**). To sum up, exosomes with ELFN1-AS1 promotes tumor growth *via* inducing macrophage M2 polarization *in vivo*.

## Discussion

Evidences have shown that the aberrant expression of lncRNAs play important roles in the progression of human cancers *via* functioning as tumor suppressors or oncogenes (31, 32). In this study, we found that ELFN1-AS1 is significantly upregulated in OS tissues and in OS cells. In addition, overexpression of ELFN1-AS1 markedly promoted the proliferation, migration and invasion of OS cells. Our finding consistent with a previous study reported that ELFN1-AS1 promoted the proliferation and migration of colorectal cancer cells *via* sponging miR-4644 (15). In addition, zhang et al. indicated that downregulation of ELFN1-AS1 was able to reduce esophageal cancer cell invasion *via* sponging miR-183-5p (14). All these data suggested that ELFN1-AS1 may function as an oncogene in OS, and it may facilitate the tumorgenesis of OS.

**Figure 9:**
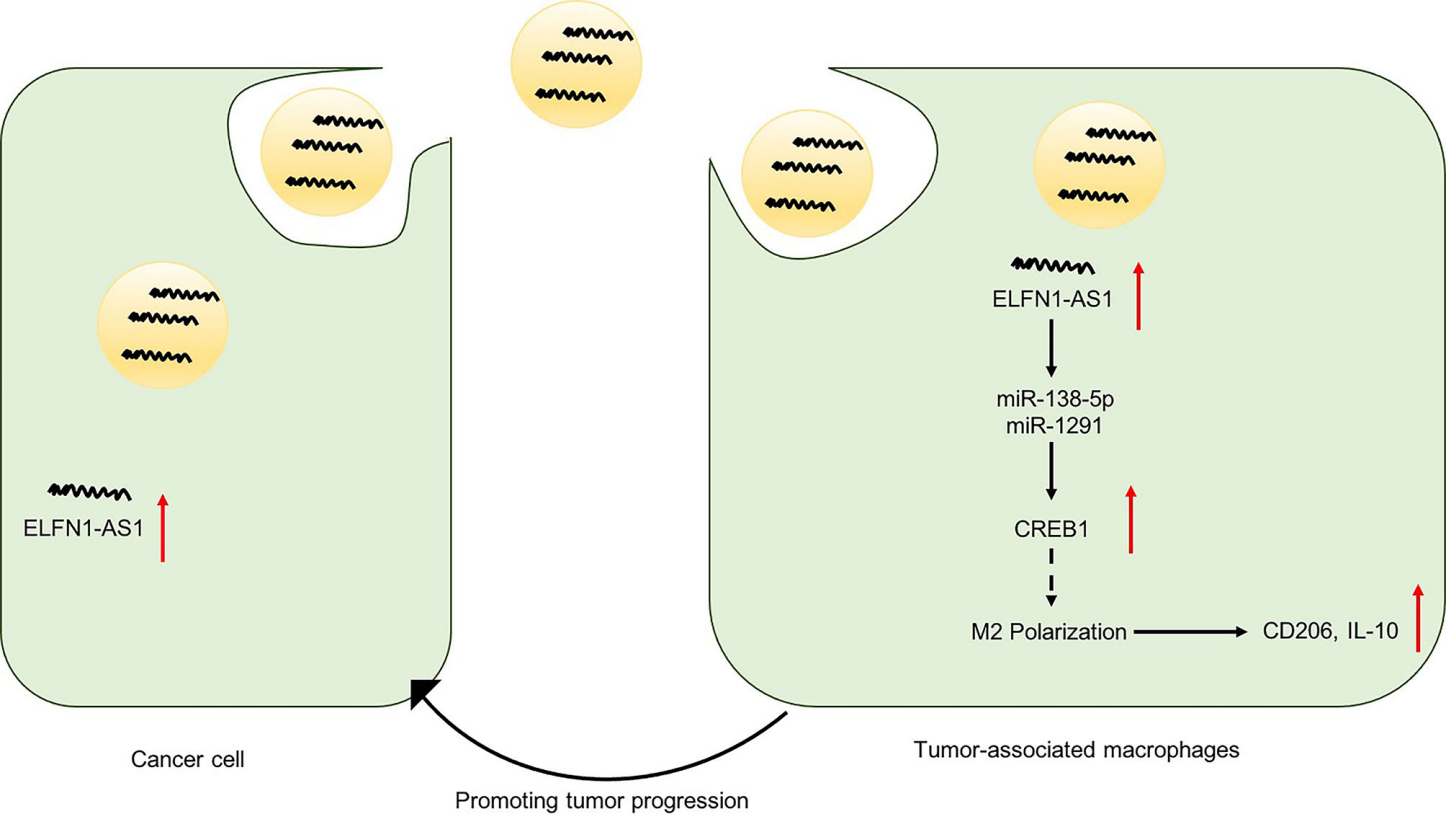
A schematic model of ELFN1-AS1 functions in tumor progression. ELFN1-AS1 could sponge miR-138-5p and miR-1291 to promote macrophage M2 polarization in macrophages *via* CREB1 upregulation, which in return promote OS progression.

Importantly, lncRNAs affect cancer progression not only by functioning within cancer cells but also by affecting the TME (26). It is reported that TME constantly interact with the cancer cells to promote their progress *via* secretion of extracellular vesicles (33, 34). In addition, cancer cells actively produce and release exosomes carrying lncRNAs to interact with various cells in the TME (35, 36). TAMs are an abundant cell type in the TME, which play a vital role during the progression of tumor (19, 37). Immunosuppressive M2-like TAMs, a pro-tumor phenotype TAMs, can foster tumor development and metastasis (38, 39). Liang et found that exosomal lncRNA RPPH1 could promote colorectal cancer metastasis by triggering macrophage M2 polarization (26). In the present study, we found that 143B cell-secreted ELFN1-AS1 could be delivered into macrophages *via* exosomes. Then, exosomal ELFN1-AS1 contributed to OS cell migration and invasion *via* inducing M2 macrophage activation. Furthermore, exosomes containing ELFN1-AS1 promoted tumor growth in an animal experiment through inducing macrophage M2 polarization *in vivo*. All these results illustrated that exosomal ELFN1-AS1 was able to promote macrophage M2 polarization, and M2 macrophage in return promoted OS progression (**Figure 9**).

It has been shown that lncRNAs may act as a ceRNA to interact with various miRNAs (40). Gao et al. found that lncRNA NEAT1 promoted M2 macrophage polarization *via* miR-214/B7-H3 axis (41). Thus, we focus on investigating the functional lncRNA-miRNA-mRNA network in macrophage polarization. In this study, we found that miR-138-5p, miR-1291 and miR-663b might be sponged by ELFN1-AS1, which was verified by luciferase reporter assay and RNA-pull down assay. In addition, CREB1 may be a common downstream target gene of miR-138-5p, miR-1291 and miR-663b. Previous studies showed that activation of CREB1 could induce macrophage M2 polarization *via* upregulation of IL-10 (42, 43). Consistent with previous studies, we found that overexpression of ELFN1-AS1 could increase the expression of CREB1 in macrophages. Meanwhile, either ELFN1-AS1 knockdown or CREB1 knockdown was able to inhibit macrophages M2 polarization *via* downregulation of CD206 and IL-10. These data showed that ELFN1-AS1 could sponge miR-138-5p and miR-1291 to promote macrophage M2 polarization in macrophages *via* CREB1 upregulation. Indeed, the interaction between ELFN1-AS1 and miR-663b in OS remains unclear and needed more investigation.

## Conclusion

ELFN1-AS1 could serve as an oncogene in OS. In addition, OS cell-derived exosomal ELFN1-AS1 could induce macrophages M2 polarization *via* sponging miR-138-5p and miR-1291, and M2 macrophages promoted the migration and invasion of OS cells. Our study may provide some theoretical support for exploring novel effective therapies for patient with OS.

## Data Availability Statement

The raw data supporting the conclusions of this article will be made available by the authors, without undue reservation.

## Ethics Statement

The ethical approval was approved by the ethics committee of the Affiliated Cancer Hospital of Zhengzhou University. Written informed consent was obtained from all patients. All animal experiments were approved by the Ethics Committee of the Affiliated Cancer Hospital of Zhengzhou University and performed following the recommended procedures of National Institutes of Health guide for the care and use of laboratory animals.

## Author Contributions

BW made major contributions to the conception, design and manuscript drafting of this study. XW, PL, XN, XL, GL, and ZL were responsible for data acquisition, data analysis, data interpretation and manuscript revision. HG made substantial contributions to conception and design of the study and revised the manuscript critically for important intellectual content. All authors agreed to be accountable for all aspects of the work. All authors read and approved the final manuscript.

## Conflict of Interest

The authors declare that the research was conducted in the absence of any commercial or financial relationships that could be construed as a potential conflict of interest.

## Publisher’s Note

All claims expressed in this article are solely those of the authors and do not necessarily represent those of their affiliated organizations, or those of the publisher, the editors and the reviewers. Any product that may be evaluated in this article, or claim that may be made by its manufacturer, is not guaranteed or endorsed by the publisher.

## Supplementary Material

Supplementary Figure 1 The level of ELFN1-AS1 is upregulated in SARC tissues.

Supplementary Figure 2 The levels of AC100778.3, AL355490.2, AC021739.4 were upregulated in OS cell-derived exosomes.

Supplementary Figure 3 CREB1 siRNA reduced the expression of CREB1 in macrophages.

Supplementary Figure 4 ELFN1-AS1 promoted macrophage M2 polarization via regulating the miR-663b/CREB1 axis.
