# Supplementary material for: Osteosarcoma Cell-Derived Exosomal ELFN1-AS1 Mediates Macrophage M2 Polarization via Sponging miR-138-5p and miR-1291 to Promote the Tumorgenesis of Osteosarcoma
Source: Front Oncol. 2022 Jun 17;12:881022. doi: 10.3389/fonc.2022.881022 (PMC9248260; doi:10.3389/fonc.2022.881022)
Supplement: Supplementary Figure 1 — The level of ELFN1-AS1 is upregulated in SARC tissues. [file DataSheet_1.zip › Figure 3/A/143B-Exo.pdf]

Electrophoresis & Brownian Motion
Video Analysis
Laser Scattering Microscopy

Operator (Report): ZetaView
Video Operator: ZetaView

**Sample Parameters**
Sample Name: WJN_A2
Comment: ZP PS100nm, Sample Remarks0:
Sample Remarks1:
Sample Remarks2:
Electrolyte: BI PBS
Temperature: 24.70 °C sensed
pH 7.0 entered
Conductivity: 15000.00 µS/cm sensed

**Result (sizes in nm)**

| | Number | Concentration | Volume |
|---|---|---|---|
| Median (X50) | 157.0 | 157.0 | 224.3 |
| Span | 61.1 | 61.1 | 79.2 |

Concentration: 2.5E+7 Particles / mL
Dilution Factor: 1000
Original Concentration: 2.5E+10 Particles / mL

**Quality**
Average Counted Particles per Frame: 71
Number of Traced Particles: 1344

**Measurement Parameters**
Cell S/N: CA16-122-0096

**Measurement Mode: Size Distribution 1 Cycles**
11 Positions, 1 Removed for Analysis

**Analysis Parameters**
Max Area: 1000, Min Area: 10, Min Brightness: 30

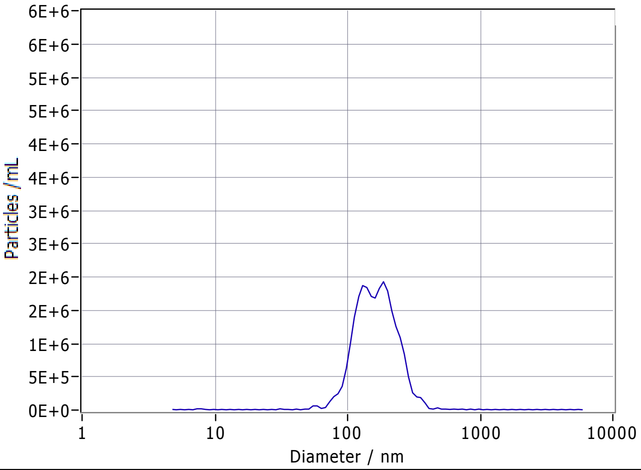


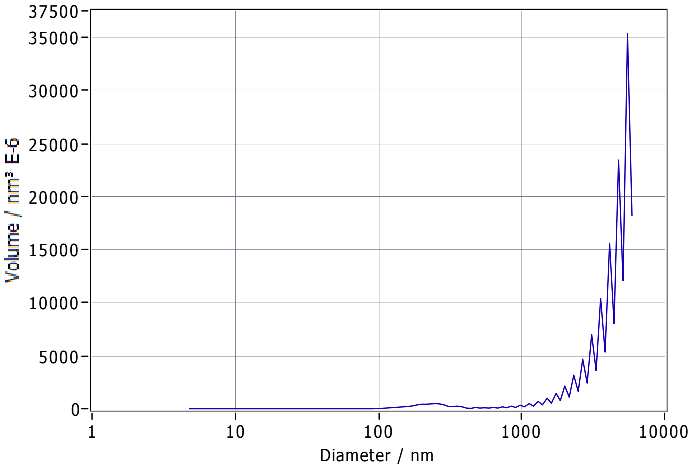


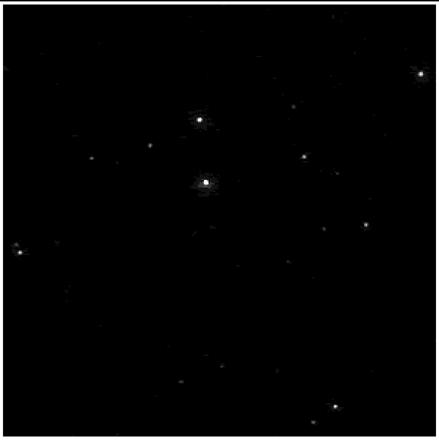

**Peak Analysis (Concentration)**

| Diameter / nm | Particles/mL | FWHM / nm | Percentage |
|---|---|---|---|
| 171.0 | 1.8E+6 | 153.4 | 97.6 |
| 54.9 | 6.1E+4 | 11.4 | 0.2 |
| 7.5 | 1.9E+4 | 1.2 | 0.4 |
| 512.0 | 1.7E+4 | 47.6 | 0.5 |
| 32.8 | 1.3E+4 | 5.1 | 0.2 |

**X Values**

| | Number | Concentration | Volume |
|---|---|---|---|
| X10 | 102.8 | 102.8 | 142.3 |
| X50 | 157.0 | 157.0 | 224.3 |
| X90 | 244.2 | 244.2 | 341.6 |
| Span | 0.9 | 0.9 | 0.9 |
| Mean | 172.4 | 172.4 | 242.0 |
| StdDev | 61.1 | 61.1 | 79.2 |

**Comment**

(Signature)

Analyzed Video: Z:\ZetaViewResults\20210304_WJN_A2_size.avi

ZetaVIEW S/N 252, Software ZetaView 8.04.02 SP2, Camera 0.703 µm/px

Experiment: 2021-03-04 10:58, Report: 2021-03-04 11:00
