# Supplementary material for: Osteosarcoma Cell-Derived Exosomal ELFN1-AS1 Mediates Macrophage M2 Polarization via Sponging miR-138-5p and miR-1291 to Promote the Tumorgenesis of Osteosarcoma
Source: Front Oncol. 2022 Jun 17;12:881022. doi: 10.3389/fonc.2022.881022 (PMC9248260; doi:10.3389/fonc.2022.881022)
Supplement: Supplementary Figure 1 — The level of ELFN1-AS1 is upregulated in SARC tissues. [file DataSheet_1.zip › Figure 3/A/hFOB-Exo.pdf]

Electrophoresis & Brownian Motion
Video Analysis
Laser Scattering Microscopy

www.particle-metrix.de

Operator (Report): ZetaView

Video Operator: ZetaView

Sample Parameters

Sample Name: WJN_B2
Comment: ZP PS100nm, Sample Remarks0:
Sample Remarks1:
Sample Remarks2:
Electrolyte: BI PBS
Temperature: 27.52 °C sensed
pH 7.0 entered
Conductivity: 15000.00 µS/cm sensed

Measurement Parameters

Cell S/N: CA16-122-0096

Measurement Mode: Size Distribution 1 Cycles

11 Positions, 1 Removed for Analysis

Result (sizes in nm)

	Number	Concentration	Volume
Median (X50)	154.5	154.5	231.3
Span	67.4	67.4	124.0

Concentration: 5.5E+7 Particles / mL
Dilution Factor: 2000
Original Concentration: 1.1E+11 Particles / mL

Quality

Average Counted Particles per Frame: 161
Number of Traced Particles: 2416

Analysis Parameters

Max Area: 1000, Min Area: 10, Min Brightness: 30

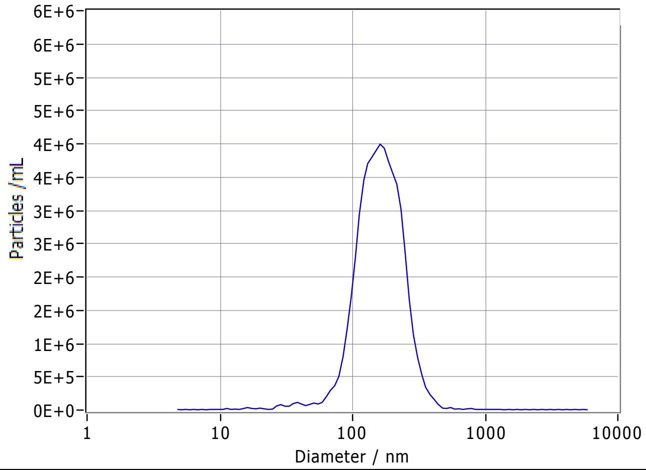

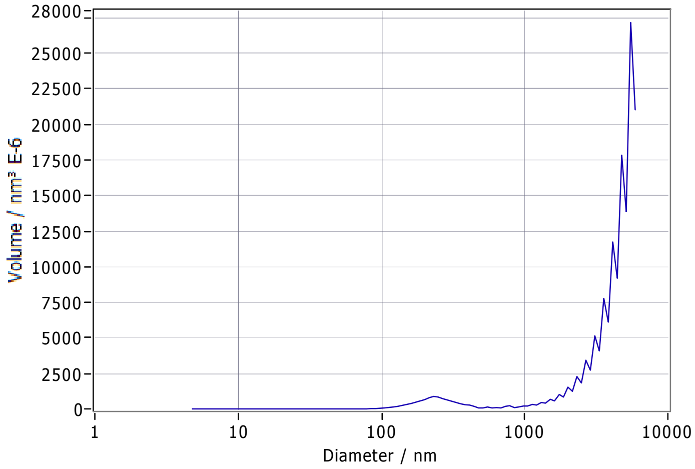

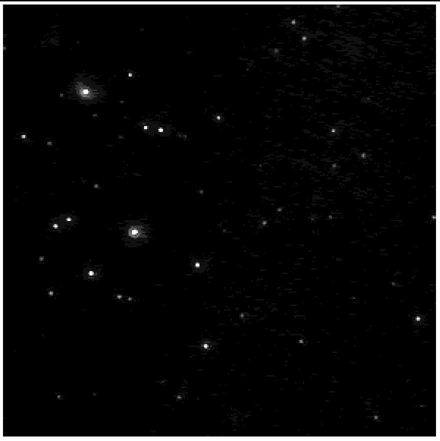

Peak Analysis (Concentration)

Diameter / nm	Particles/mL	FWHM / nm	Percentage
161.1	4.0E+6	157.6	99.1
16.1	3.5E+4	2.6	0.3
11.4	2.1E+4	1.2	0.1
784.3	2.0E+4	174.0	0.1
8.4	9.4E+3	0.7	0.1

X Values

	Number	Concentration	Volume
X10	94.8	94.8	145.4
X50	154.5	154.5	231.3
X90	246.1	246.1	385.5
Span	1.0	1.0	1.0
Mean	170.1	170.1	266.6
StdDev	67.4	67.4	124.0

Comment

(Signature)

Analyzed Video: Z:\ZetaViewResults\20210304\20210304_WJN_B2_size.avi

ZetaVIEW S/N 252, Software ZetaView 8.04.02 SP2, Camera 0.703 µm/px

Experiment: 2021-03-04 14:44, Report: 2021-03-04 14:46
