# Supplementary figures and images for: Osteosarcoma Cell-Derived Exosomal ELFN1-AS1 Mediates Macrophage M2 Polarization via Sponging miR-138-5p and miR-1291 to Promote the Tumorgenesis of Osteosarcoma (part 2 of 2)
Source: Front Oncol. 2022 Jun 17;12:881022. doi: 10.3389/fonc.2022.881022 (PMC9248260; doi:10.3389/fonc.2022.881022)

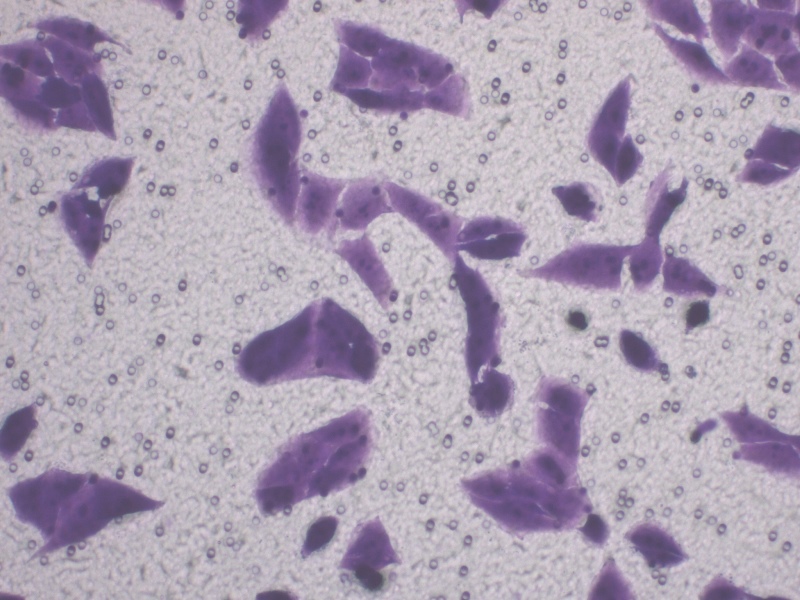

Supplement: Supplementary Figure 1 — The level of ELFN1-AS1 is upregulated in SARC tissues. [file DataSheet_1.zip › Figure 2/H/MG63 invasion/X1/1.jpg]

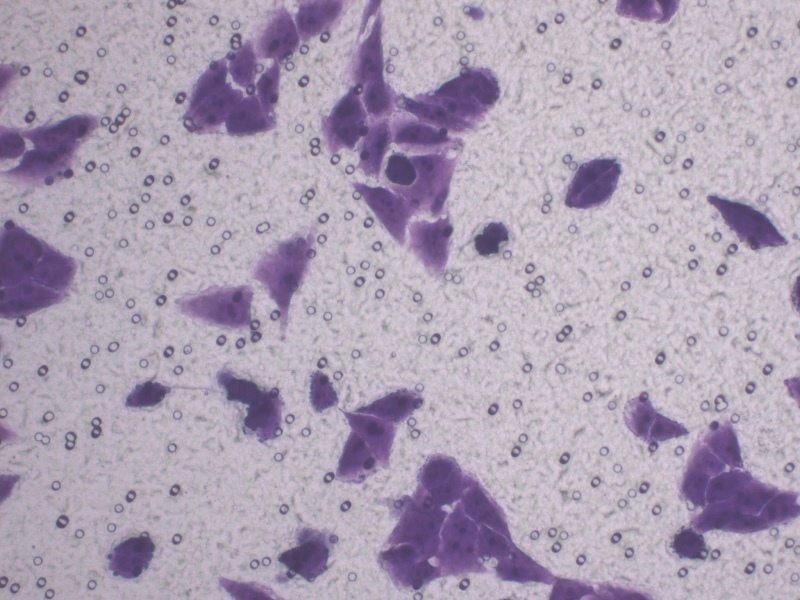

Supplement: Supplementary Figure 1 — The level of ELFN1-AS1 is upregulated in SARC tissues. [file DataSheet_1.zip › Figure 2/H/MG63 invasion/X1/2.jpg]

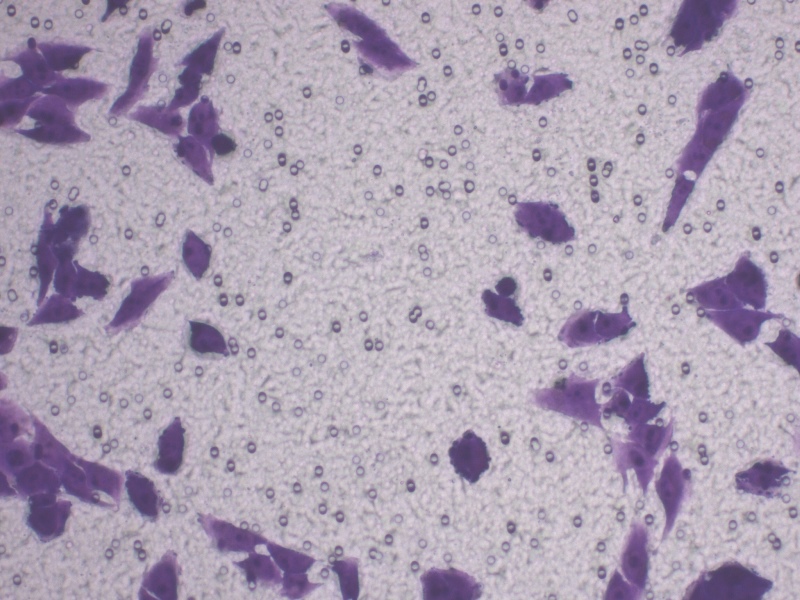

Supplement: Supplementary Figure 1 — The level of ELFN1-AS1 is upregulated in SARC tissues. [file DataSheet_1.zip › Figure 2/H/MG63 invasion/X1/3.jpg]

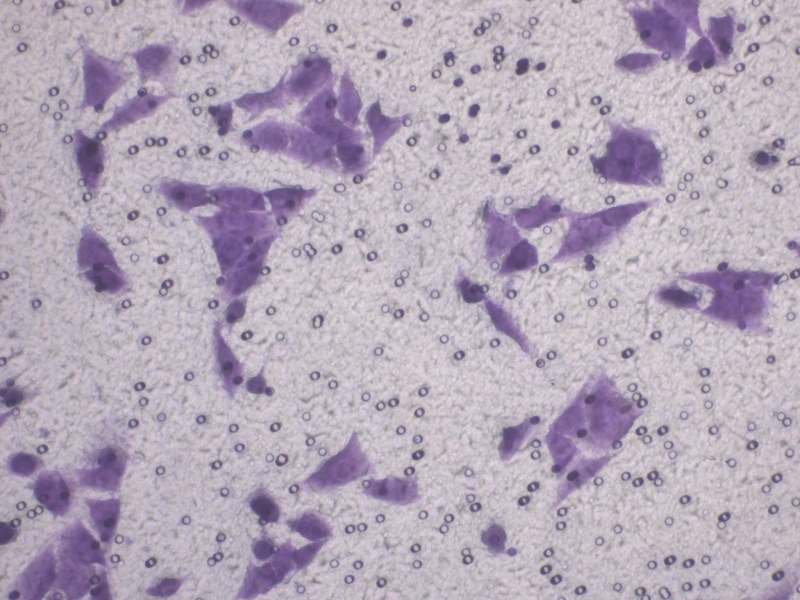

Supplement: Supplementary Figure 1 — The level of ELFN1-AS1 is upregulated in SARC tissues. [file DataSheet_1.zip › Figure 2/H/MG63 invasion/Y1/1.jpg]

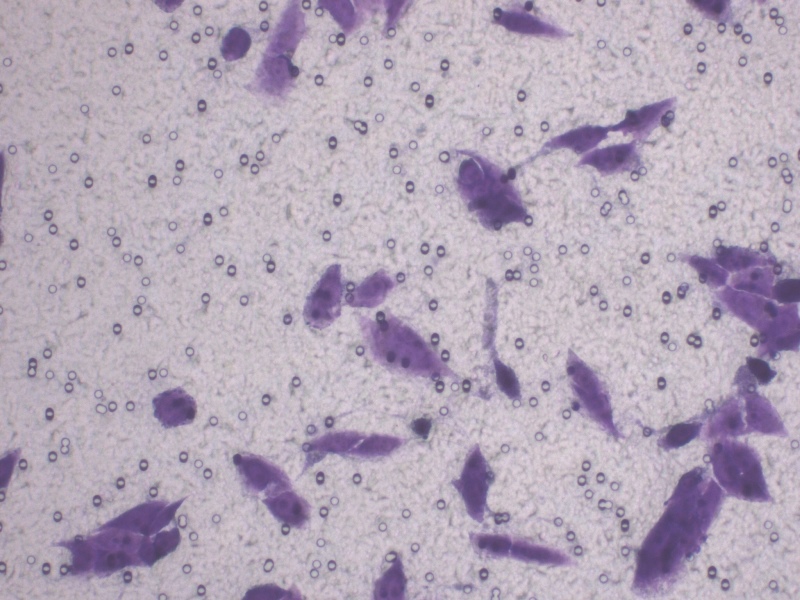

Supplement: Supplementary Figure 1 — The level of ELFN1-AS1 is upregulated in SARC tissues. [file DataSheet_1.zip › Figure 2/H/MG63 invasion/Y1/2.jpg]

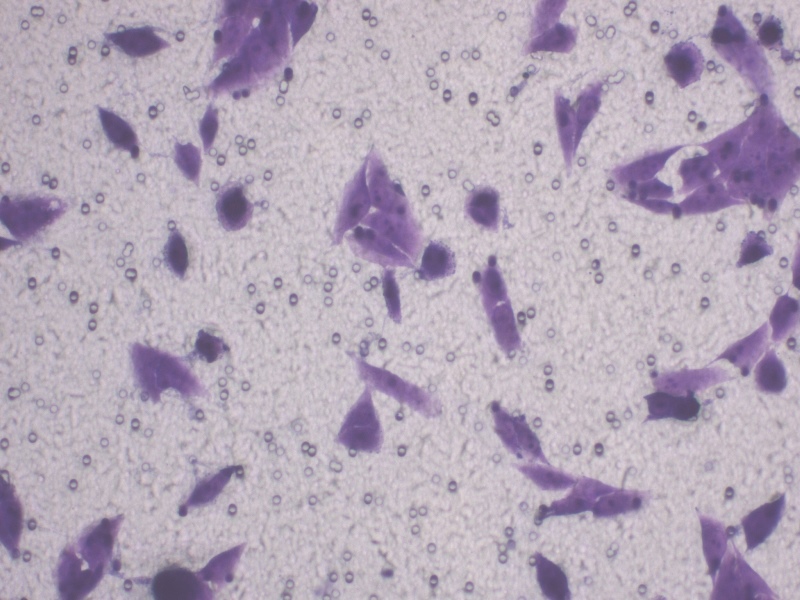

Supplement: Supplementary Figure 1 — The level of ELFN1-AS1 is upregulated in SARC tissues. [file DataSheet_1.zip › Figure 2/H/MG63 invasion/Y1/3.jpg]

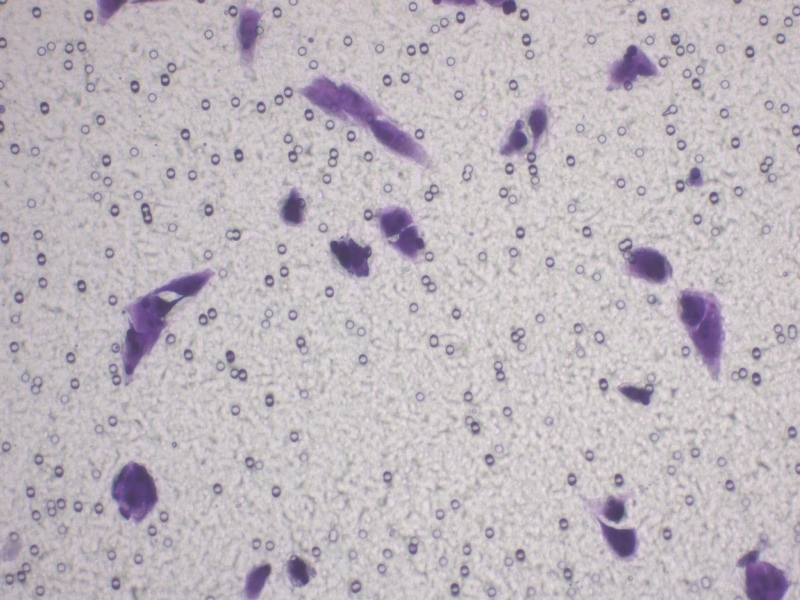

Supplement: Supplementary Figure 1 — The level of ELFN1-AS1 is upregulated in SARC tissues. [file DataSheet_1.zip › Figure 2/H/MG63 invasion/Z1/1.jpg]

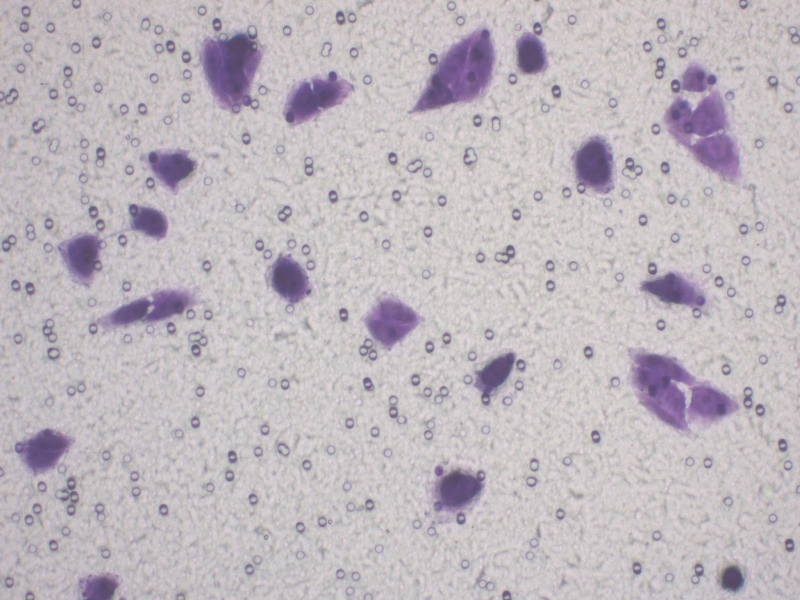

Supplement: Supplementary Figure 1 — The level of ELFN1-AS1 is upregulated in SARC tissues. [file DataSheet_1.zip › Figure 2/H/MG63 invasion/Z1/2.jpg]

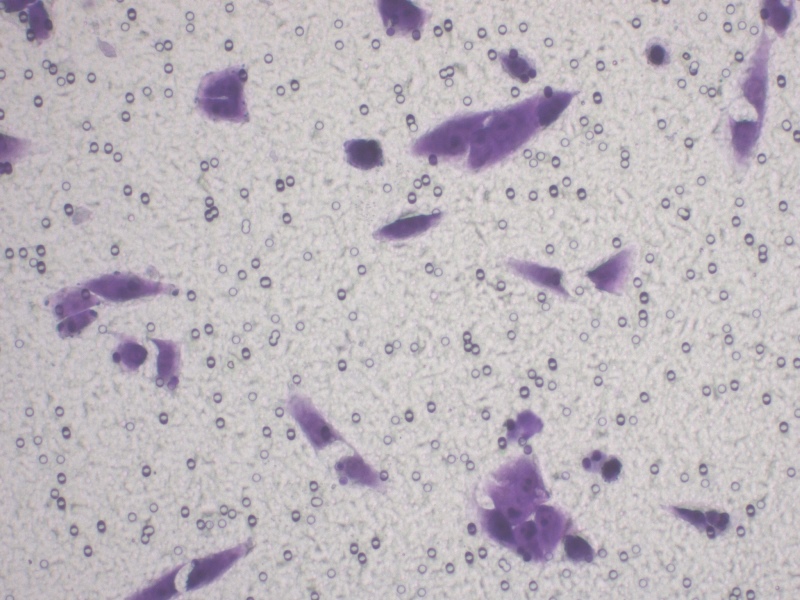

Supplement: Supplementary Figure 1 — The level of ELFN1-AS1 is upregulated in SARC tissues. [file DataSheet_1.zip › Figure 2/H/MG63 invasion/Z1/3.jpg]

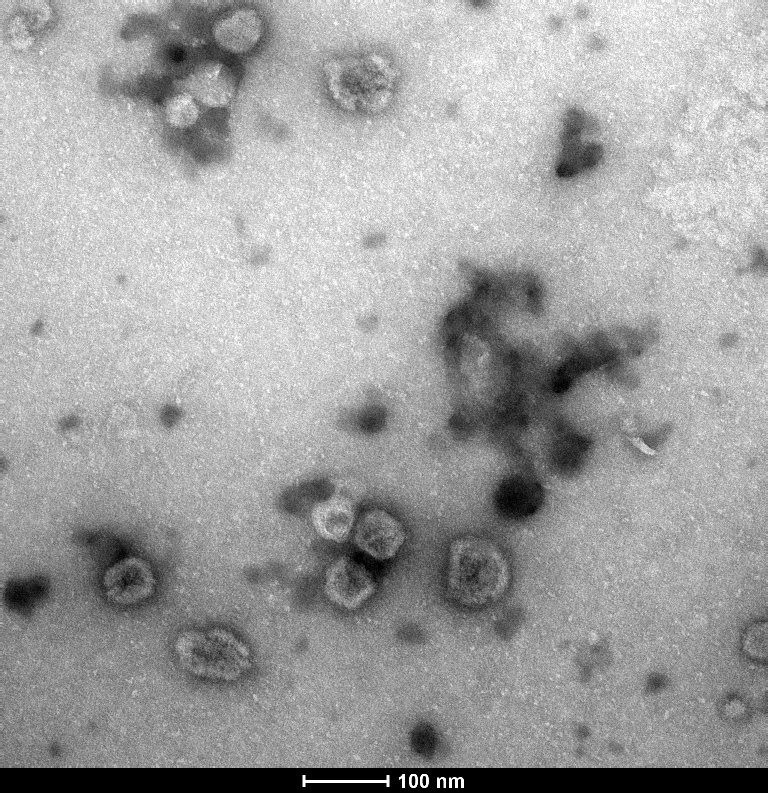

Supplement: Supplementary Figure 1 — The level of ELFN1-AS1 is upregulated in SARC tissues. [file DataSheet_1.zip › Figure 3/A/143B-Exo.tif]

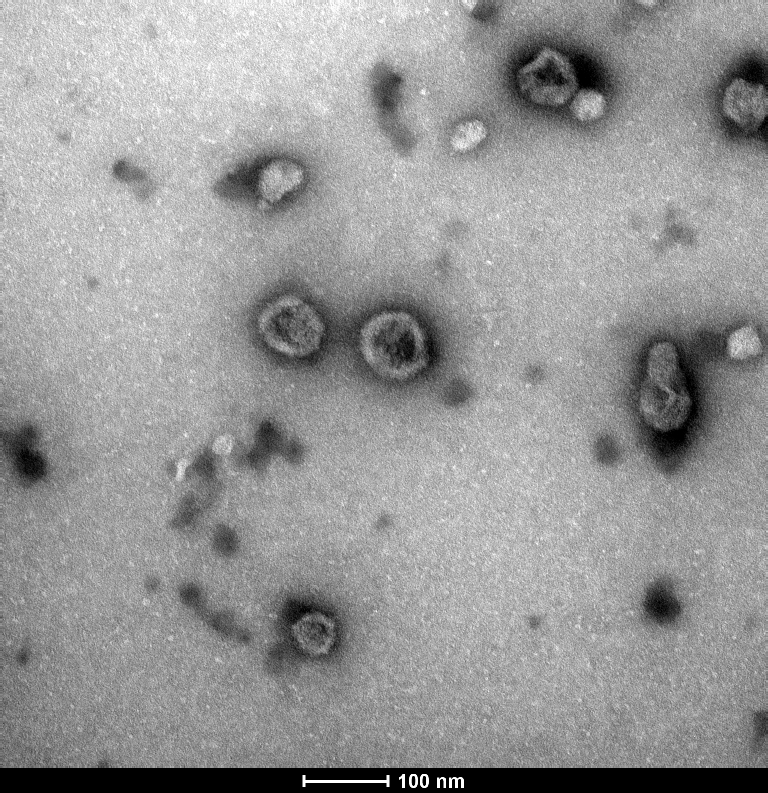

Supplement: Supplementary Figure 1 — The level of ELFN1-AS1 is upregulated in SARC tissues. [file DataSheet_1.zip › Figure 3/A/hFOB-Exo.tif]

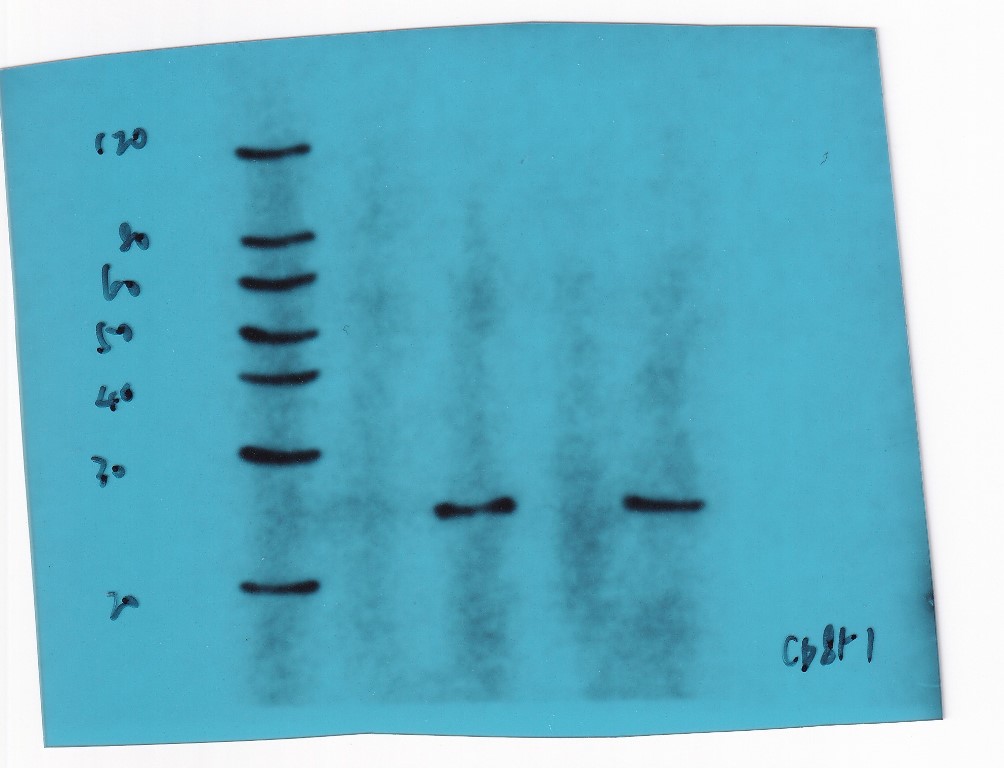

Supplement: Supplementary Figure 1 — The level of ELFN1-AS1 is upregulated in SARC tissues. [file DataSheet_1.zip › Figure 3/B/Figure 3B-CD81.jpg]

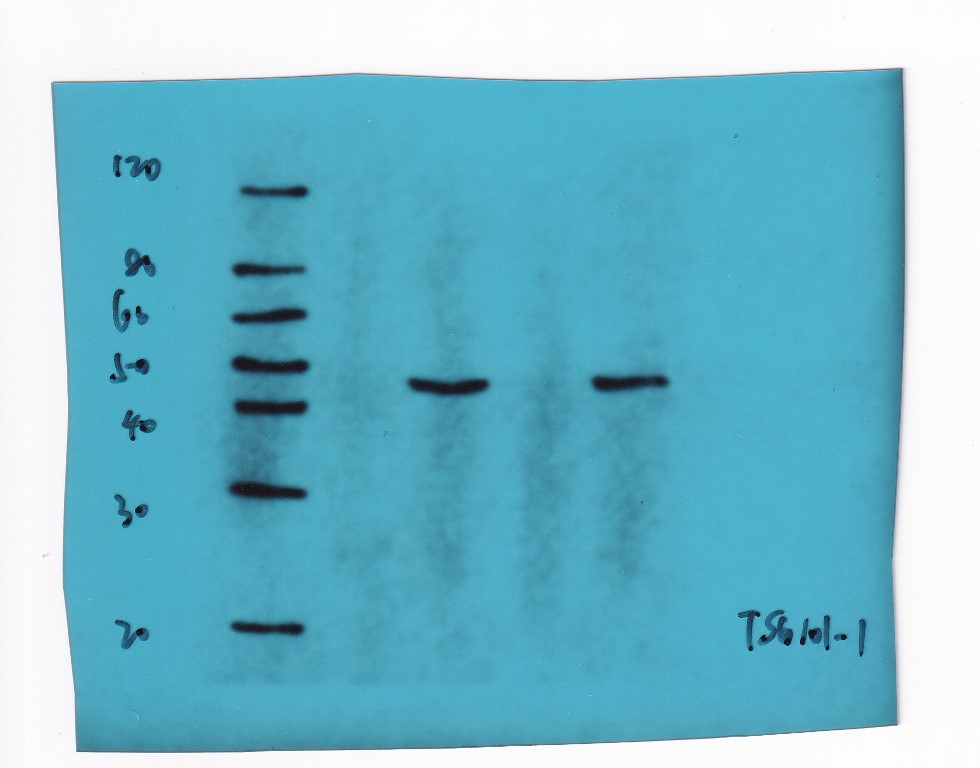

Supplement: Supplementary Figure 1 — The level of ELFN1-AS1 is upregulated in SARC tissues. [file DataSheet_1.zip › Figure 3/B/Figure 3B-TSG101.jpg]

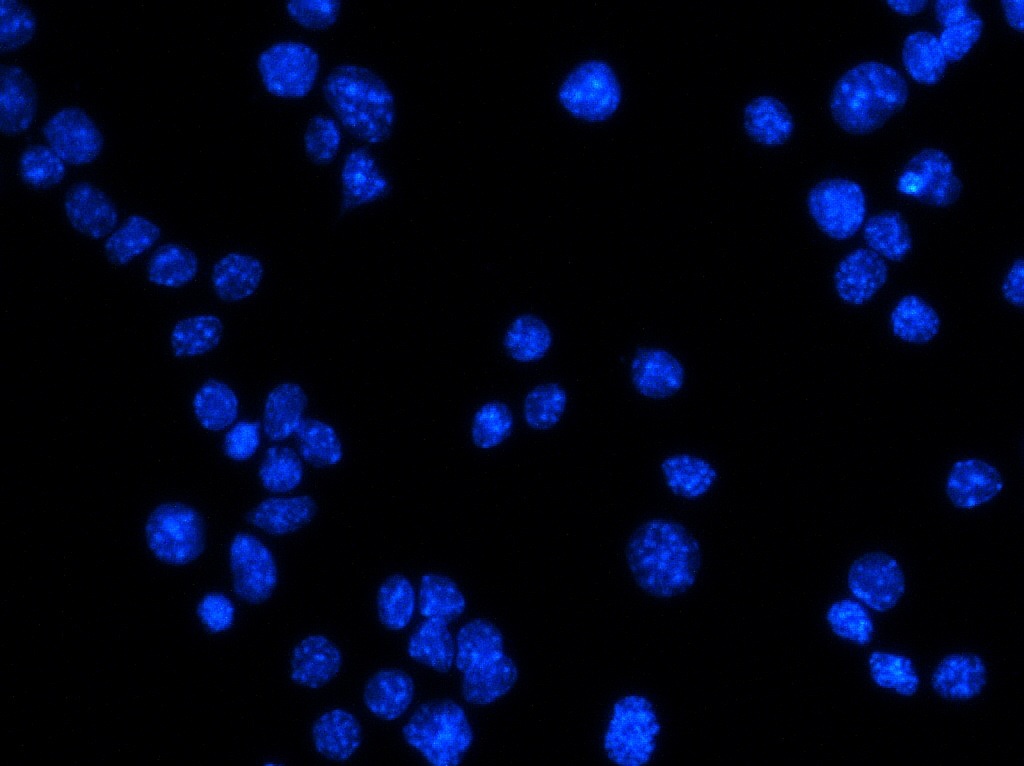

Supplement: Supplementary Figure 1 — The level of ELFN1-AS1 is upregulated in SARC tissues. [file DataSheet_1.zip › Figure 3/C/DAPI.jpg]

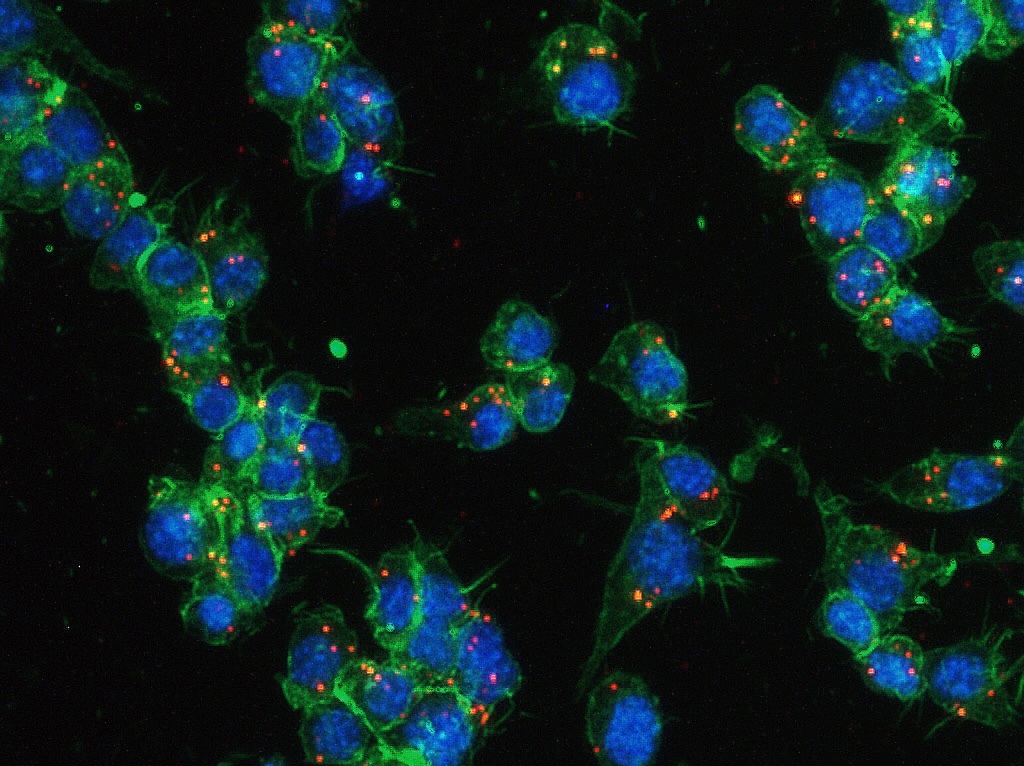

Supplement: Supplementary Figure 1 — The level of ELFN1-AS1 is upregulated in SARC tissues. [file DataSheet_1.zip › Figure 3/C/Merge.jpg]

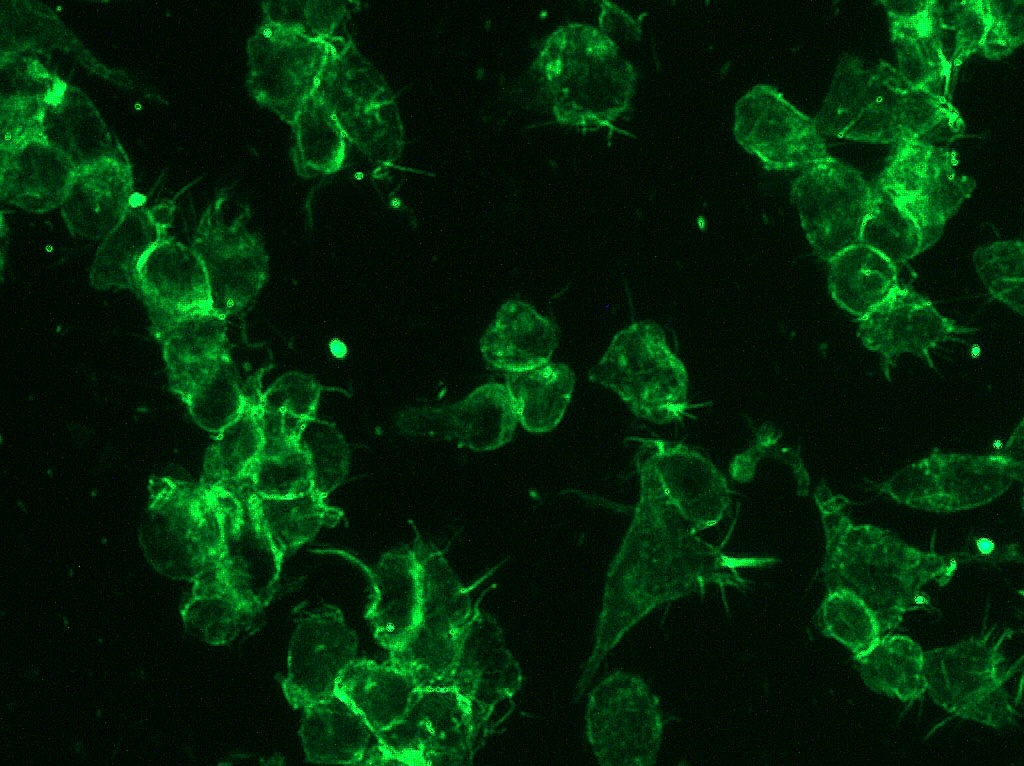

Supplement: Supplementary Figure 1 — The level of ELFN1-AS1 is upregulated in SARC tissues. [file DataSheet_1.zip › Figure 3/C/Phalloidin.jpg]

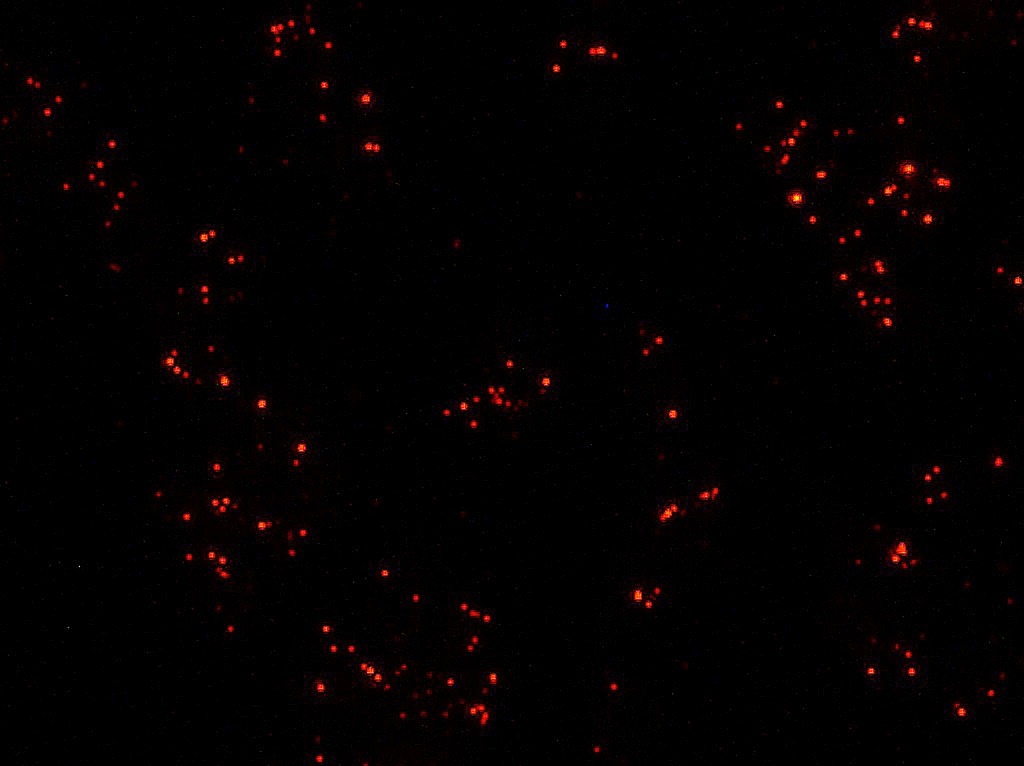

Supplement: Supplementary Figure 1 — The level of ELFN1-AS1 is upregulated in SARC tissues. [file DataSheet_1.zip › Figure 3/C/PKH26.jpg]

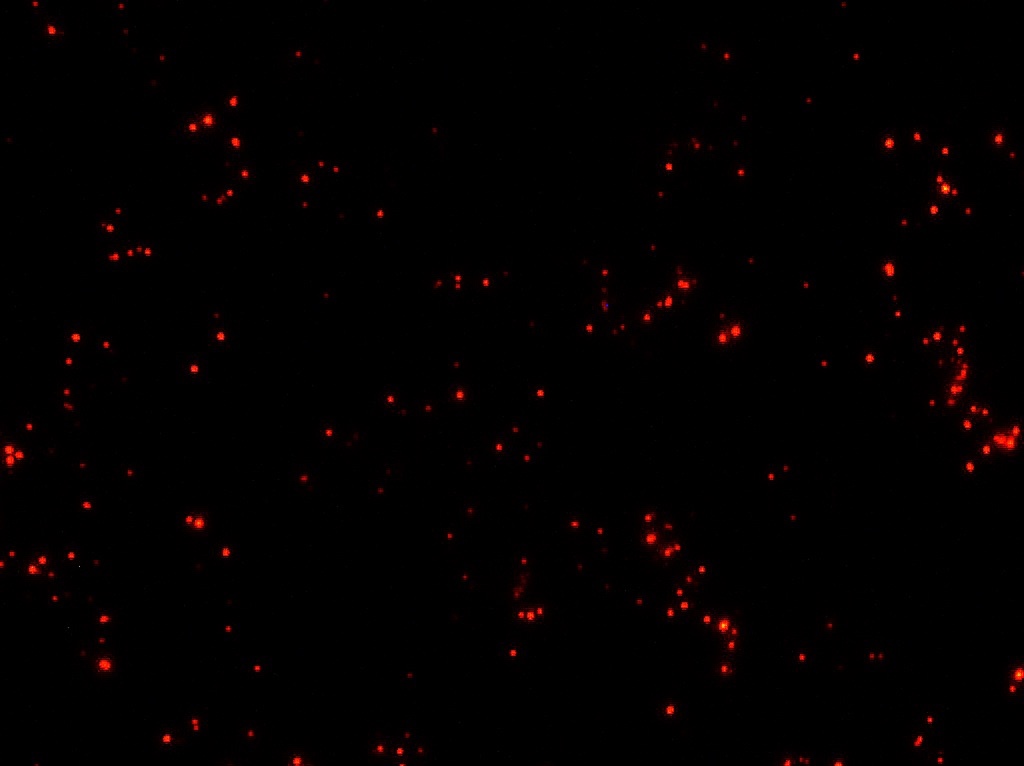

Supplement: Supplementary Figure 1 — The level of ELFN1-AS1 is upregulated in SARC tissues. [file DataSheet_1.zip › Figure 3/G/cy3.jpg]

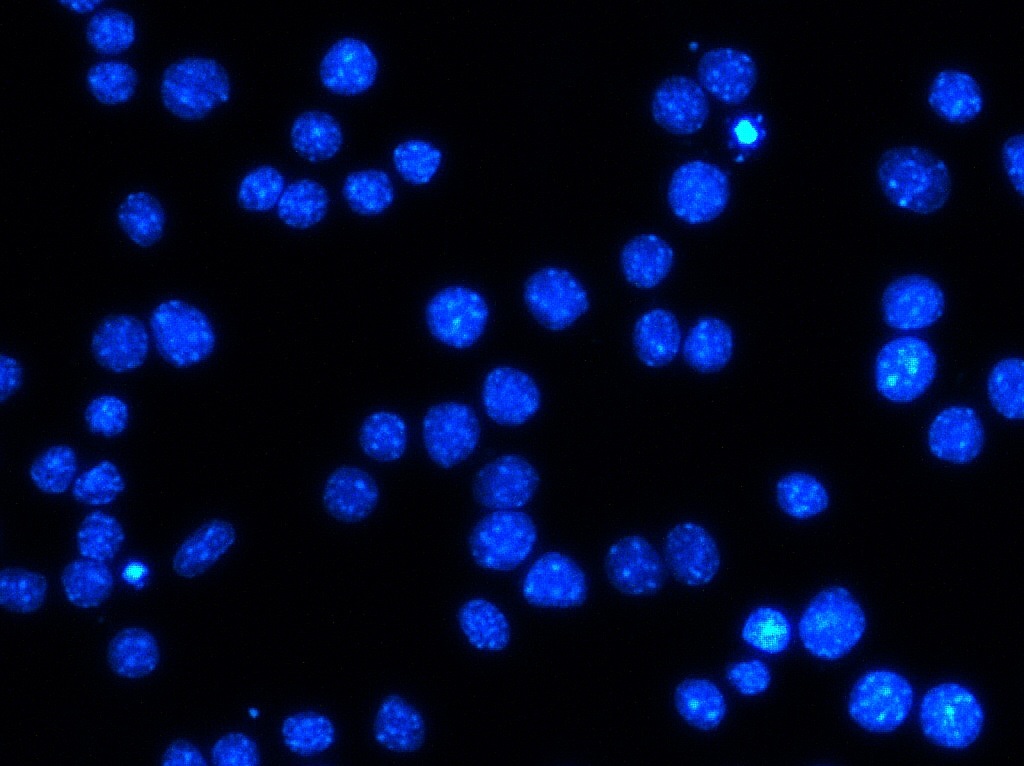

Supplement: Supplementary Figure 1 — The level of ELFN1-AS1 is upregulated in SARC tissues. [file DataSheet_1.zip › Figure 3/G/DAPI.jpg]

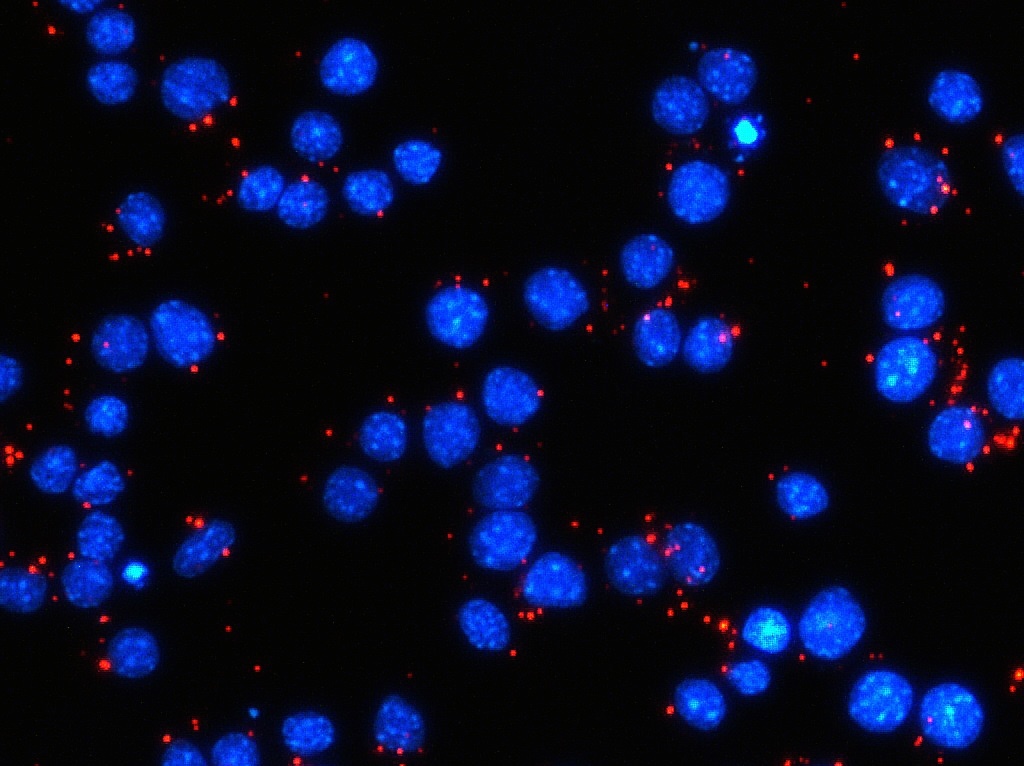

Supplement: Supplementary Figure 1 — The level of ELFN1-AS1 is upregulated in SARC tissues. [file DataSheet_1.zip › Figure 3/G/merge.JPG]

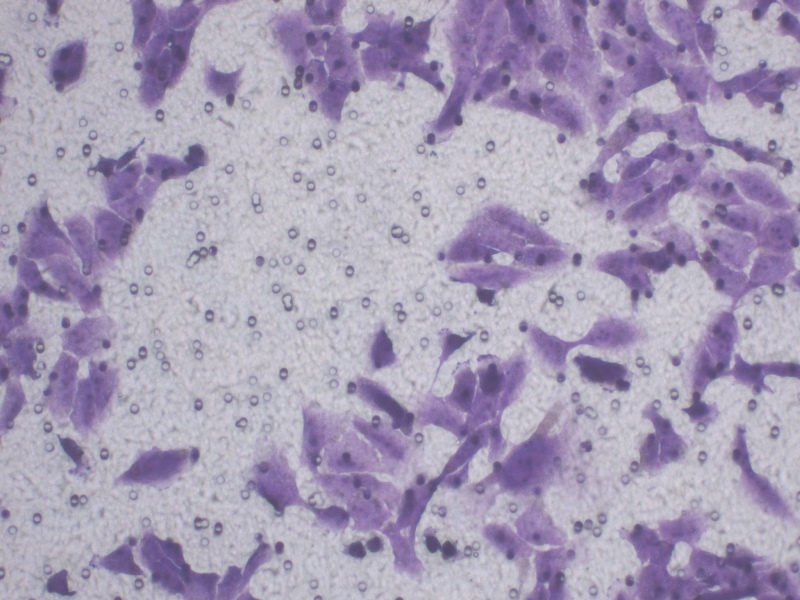

Supplement: Supplementary file 2 [file DataSheet_2.zip › data source-2/Figure 5/B/invasion/143B/1.jpg]

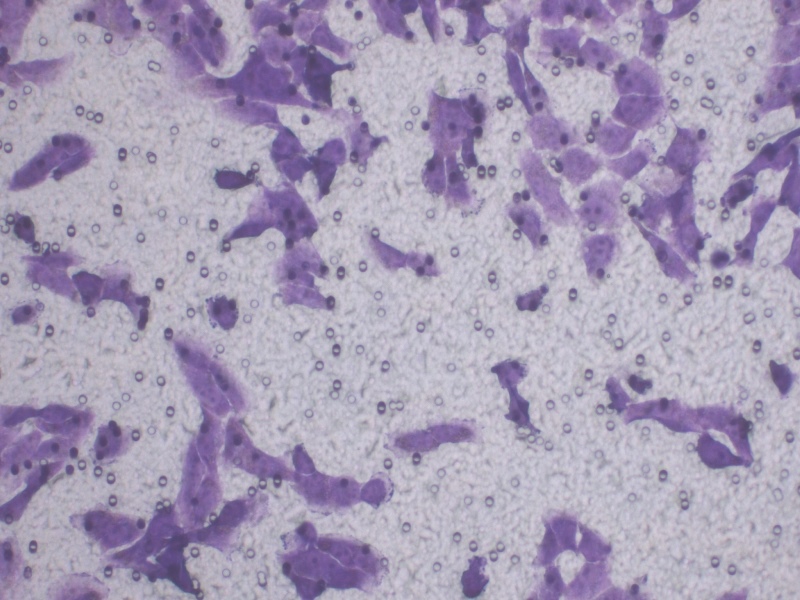

Supplement: Supplementary file 2 [file DataSheet_2.zip › data source-2/Figure 5/B/invasion/143B/2.jpg]

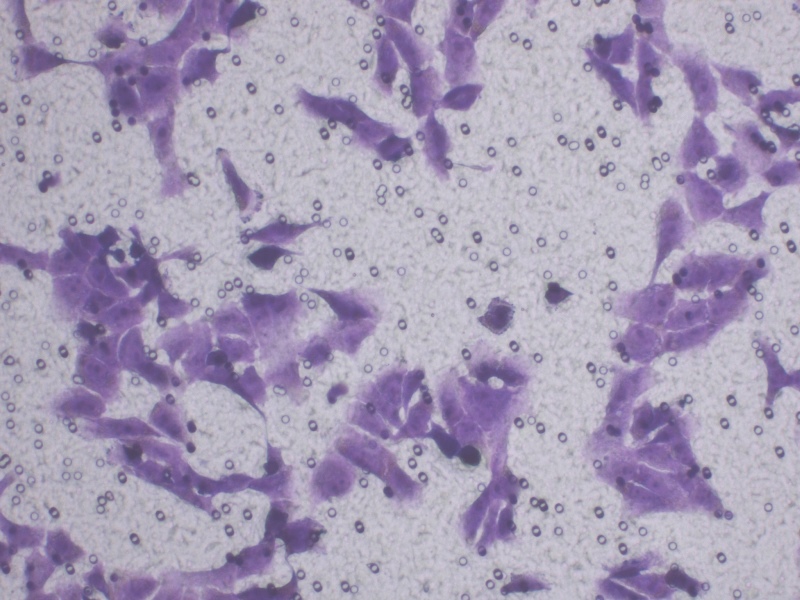

Supplement: Supplementary file 2 [file DataSheet_2.zip › data source-2/Figure 5/B/invasion/143B/3.jpg]

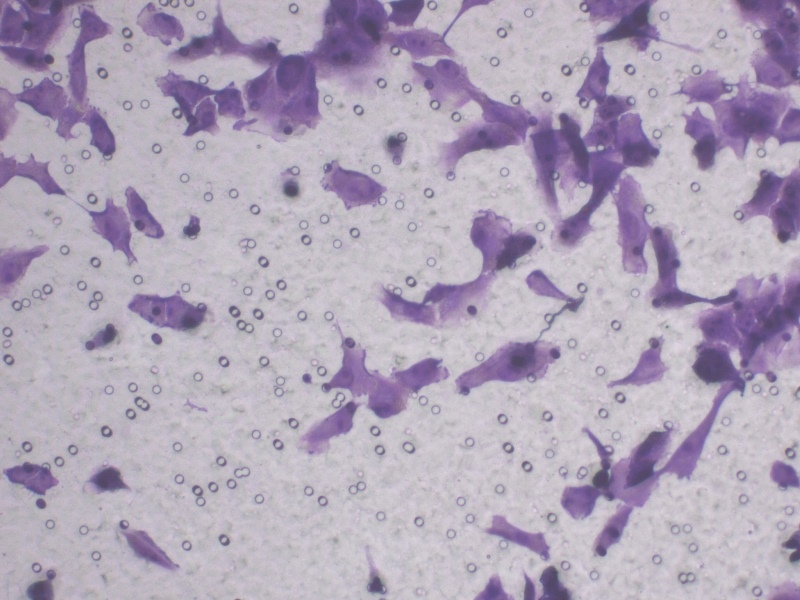

Supplement: Supplementary file 2 [file DataSheet_2.zip › data source-2/Figure 5/B/invasion/143B + M- ELFN1-AS1 siRNA/1.jpg]

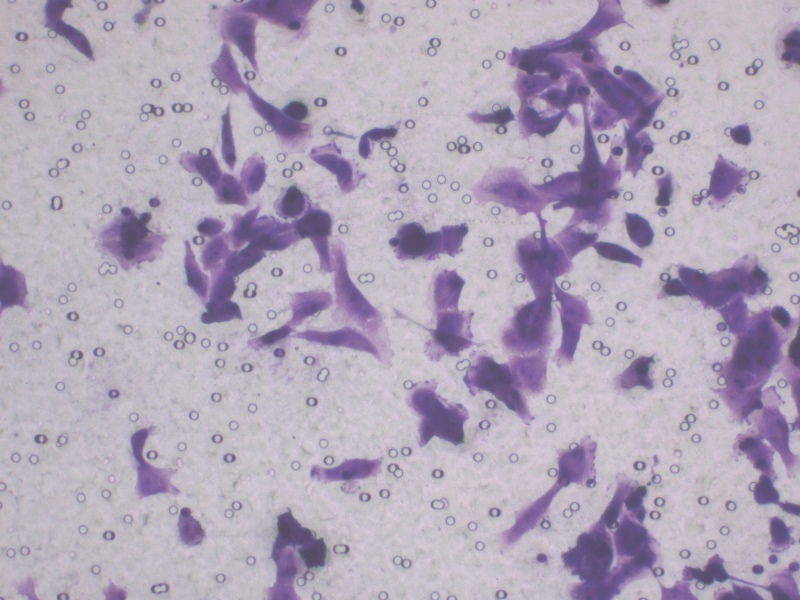

Supplement: Supplementary file 2 [file DataSheet_2.zip › data source-2/Figure 5/B/invasion/143B + M- ELFN1-AS1 siRNA/2.jpg]

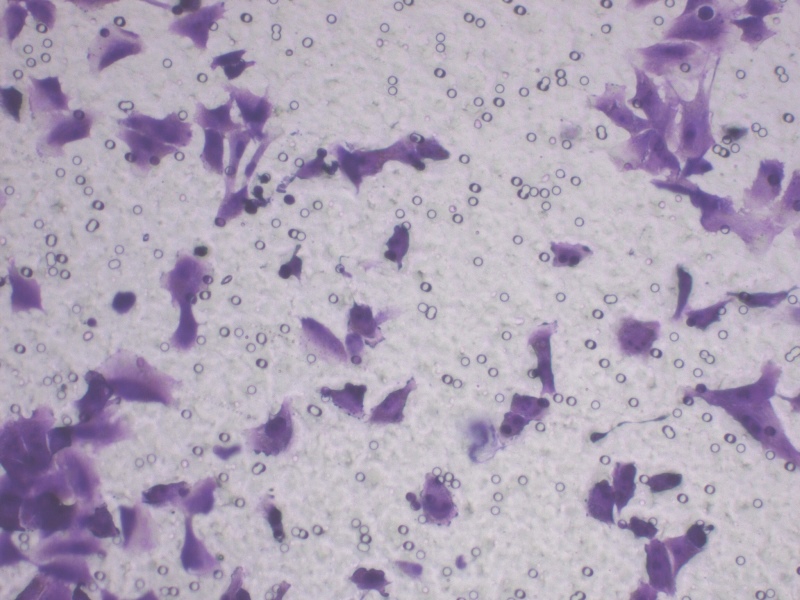

Supplement: Supplementary file 2 [file DataSheet_2.zip › data source-2/Figure 5/B/invasion/143B + M- ELFN1-AS1 siRNA/3.jpg]

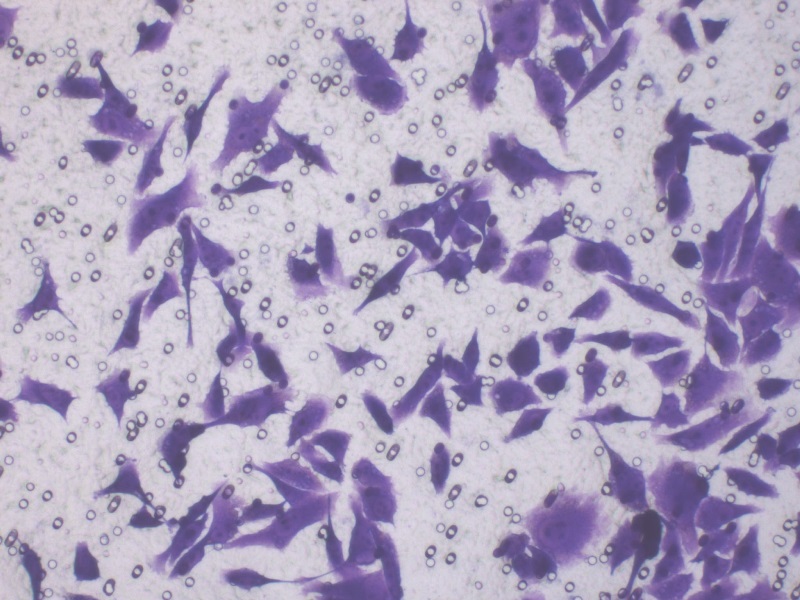

Supplement: Supplementary file 2 [file DataSheet_2.zip › data source-2/Figure 5/B/invasion/143B + M-pcDNA3.1 ELFN1-AS1/1.jpg]

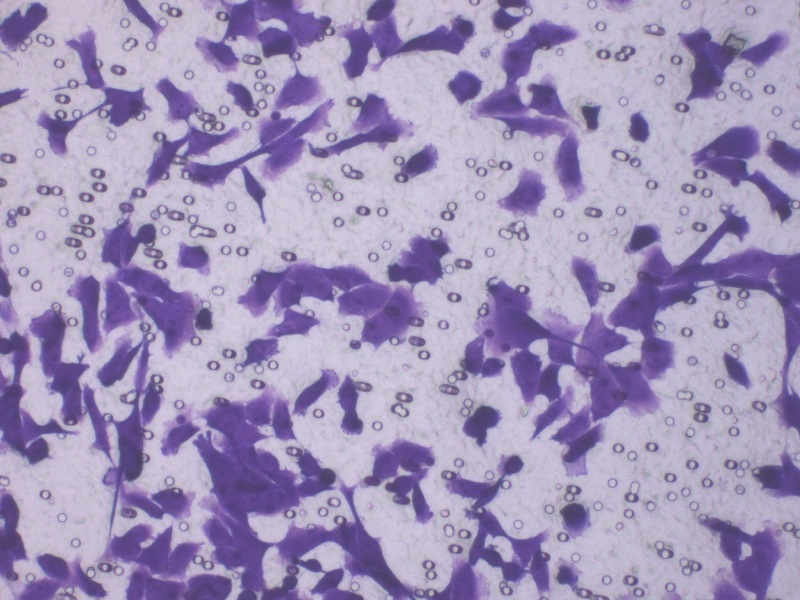

Supplement: Supplementary file 2 [file DataSheet_2.zip › data source-2/Figure 5/B/invasion/143B + M-pcDNA3.1 ELFN1-AS1/2.jpg]

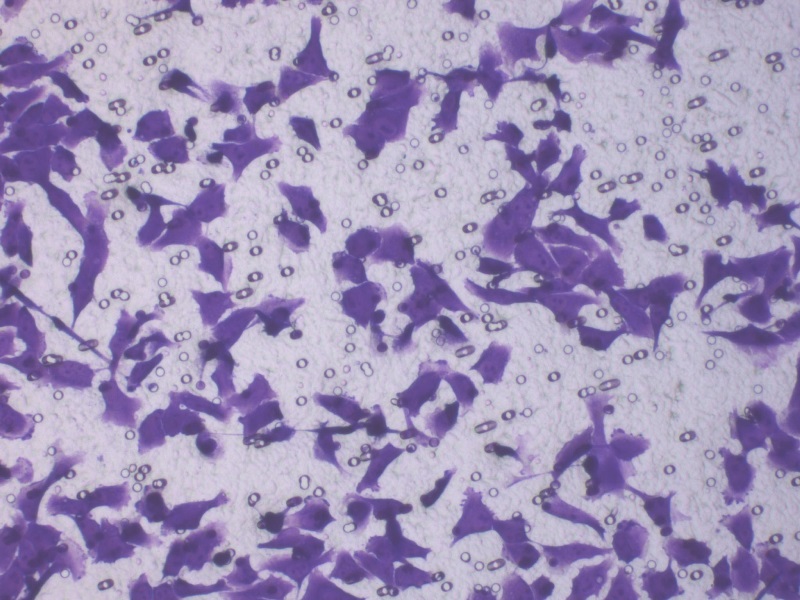

Supplement: Supplementary file 2 [file DataSheet_2.zip › data source-2/Figure 5/B/invasion/143B + M-pcDNA3.1 ELFN1-AS1/3.jpg]

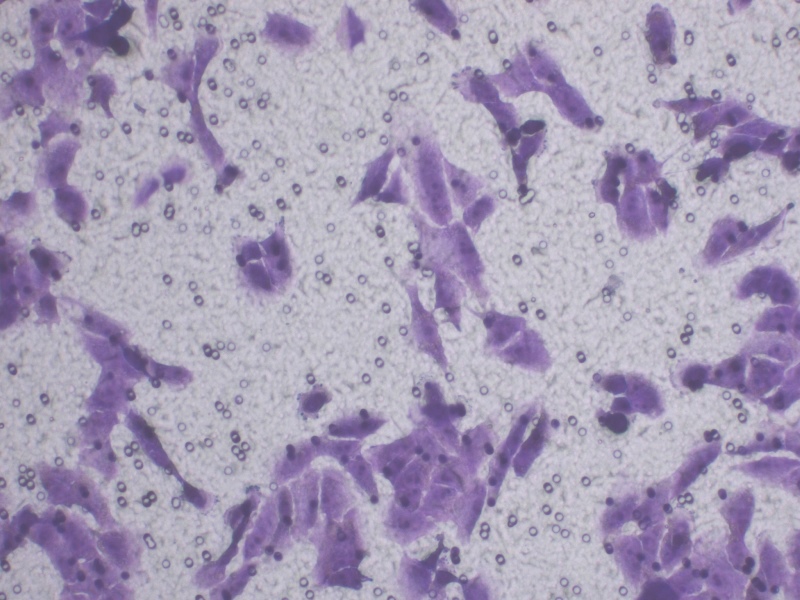

Supplement: Supplementary file 2 [file DataSheet_2.zip › data source-2/Figure 5/B/invasion/143B + M-pcDNA3.1 NC/1.jpg]

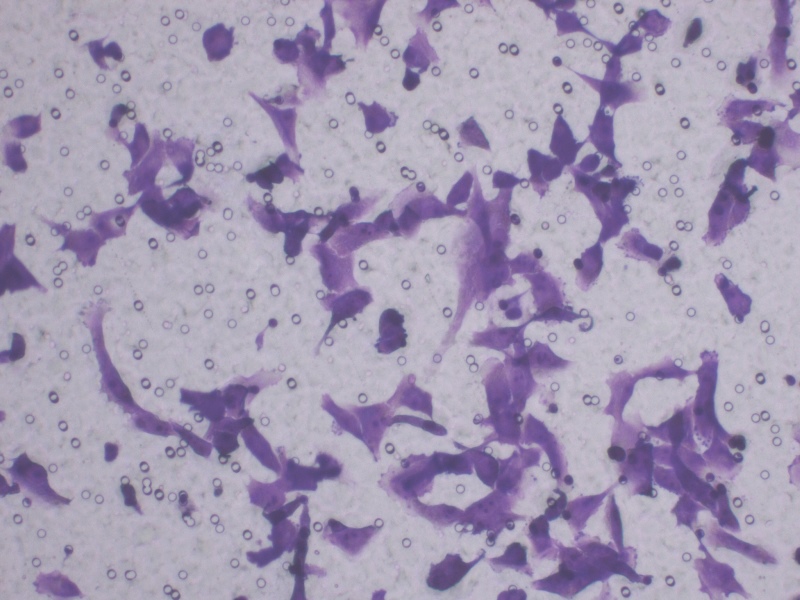

Supplement: Supplementary file 2 [file DataSheet_2.zip › data source-2/Figure 5/B/invasion/143B + M-pcDNA3.1 NC/2.jpg]

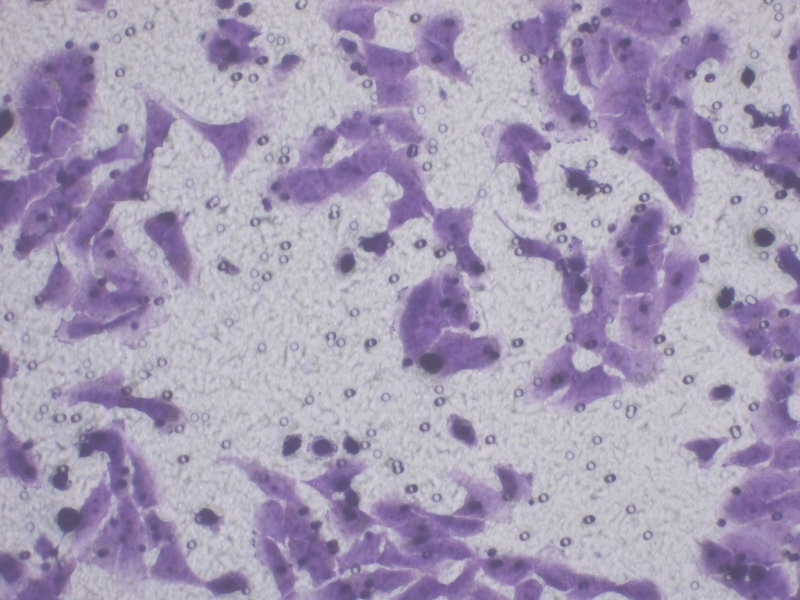

Supplement: Supplementary file 2 [file DataSheet_2.zip › data source-2/Figure 5/B/invasion/143B + M-pcDNA3.1 NC/3.jpg]

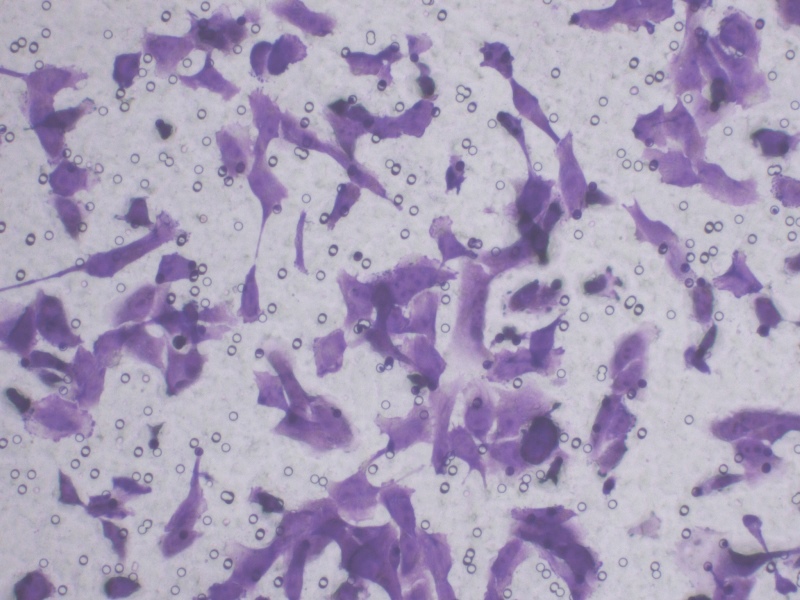

Supplement: Supplementary file 2 [file DataSheet_2.zip › data source-2/Figure 5/B/invasion/143B + MsiRNA NC/1.jpg]

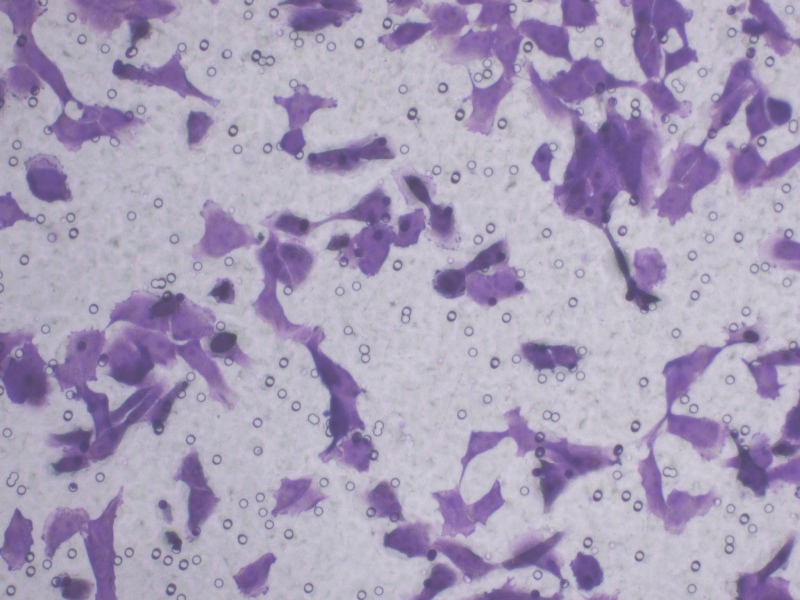

Supplement: Supplementary file 2 [file DataSheet_2.zip › data source-2/Figure 5/B/invasion/143B + MsiRNA NC/2.jpg]

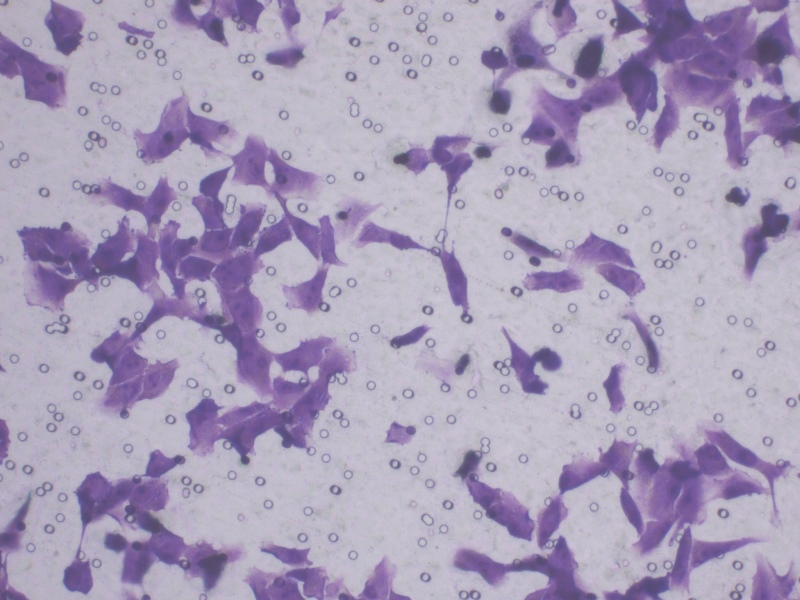

Supplement: Supplementary file 2 [file DataSheet_2.zip › data source-2/Figure 5/B/invasion/143B + MsiRNA NC/3.jpg]

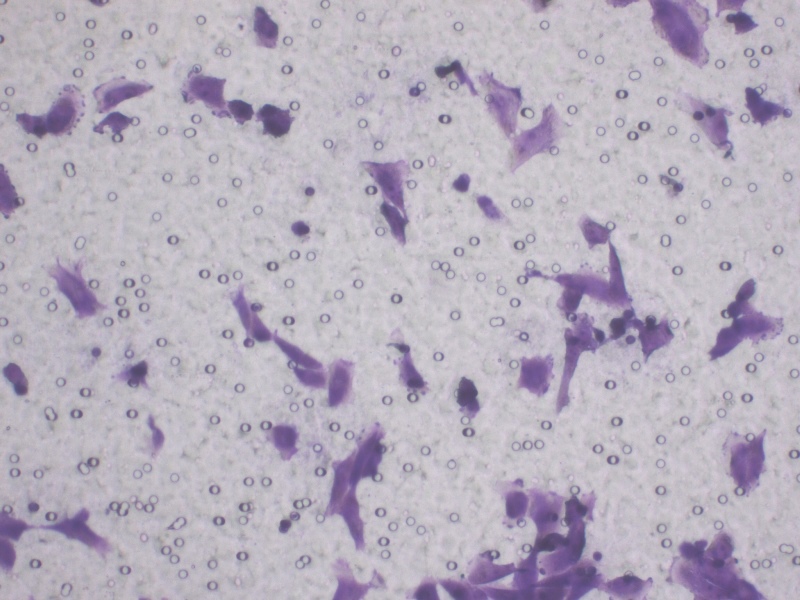

Supplement: Supplementary file 2 [file DataSheet_2.zip › data source-2/Figure 5/B/migration/143B/1.jpg]

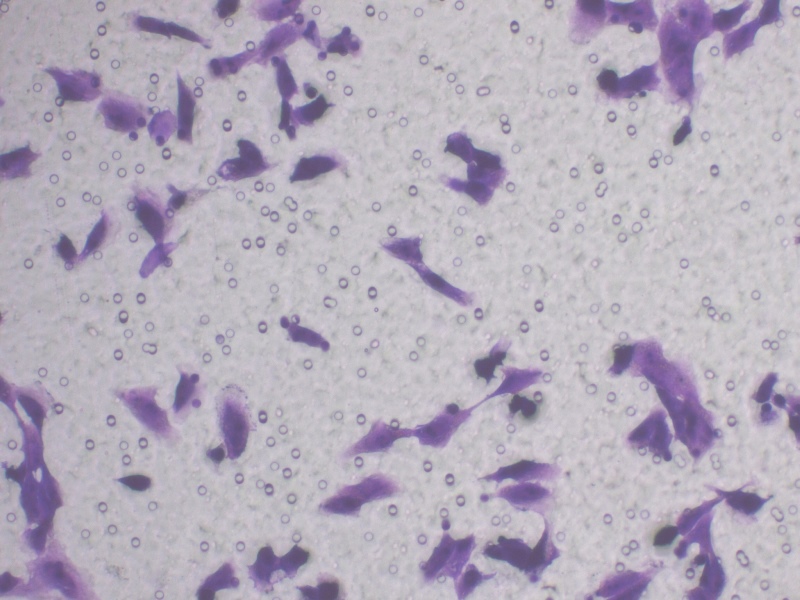

Supplement: Supplementary file 2 [file DataSheet_2.zip › data source-2/Figure 5/B/migration/143B/2.jpg]

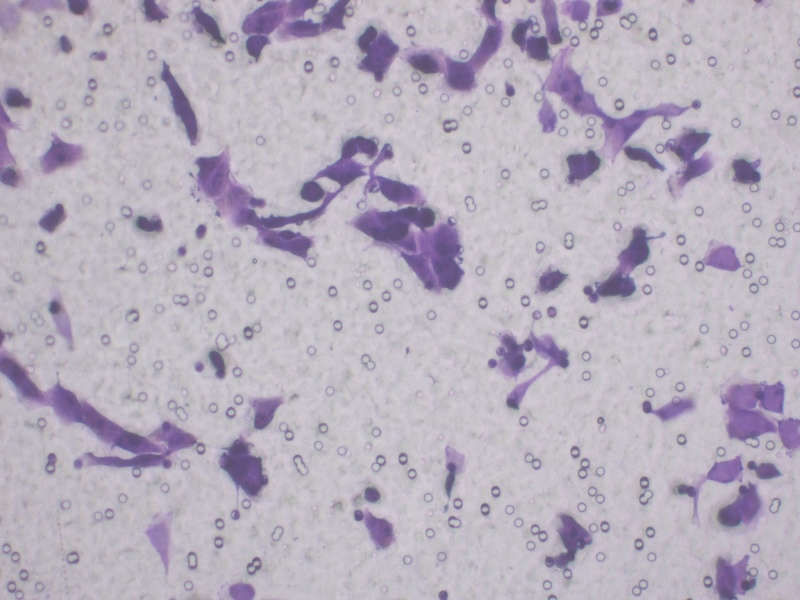

Supplement: Supplementary file 2 [file DataSheet_2.zip › data source-2/Figure 5/B/migration/143B/3.jpg]

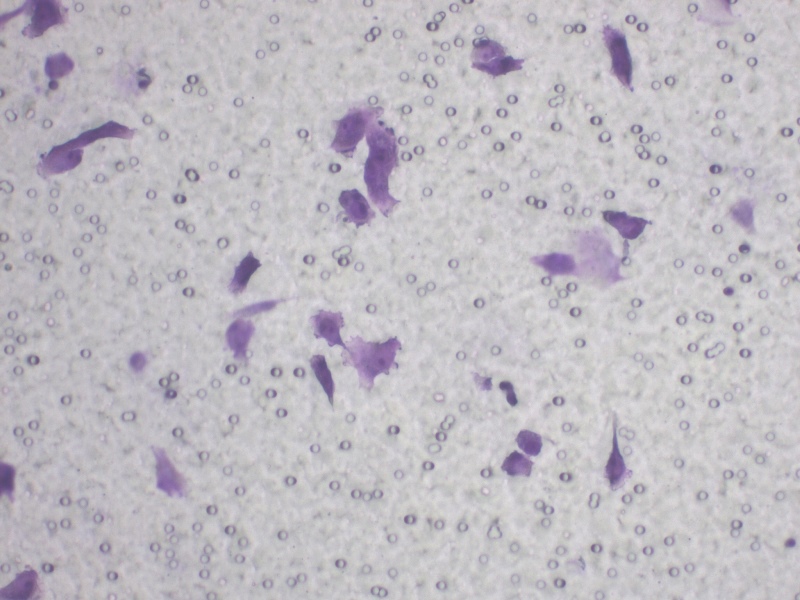

Supplement: Supplementary file 2 [file DataSheet_2.zip › data source-2/Figure 5/B/migration/143B + M- ELFN1-AS1 siRNA/1.jpg]

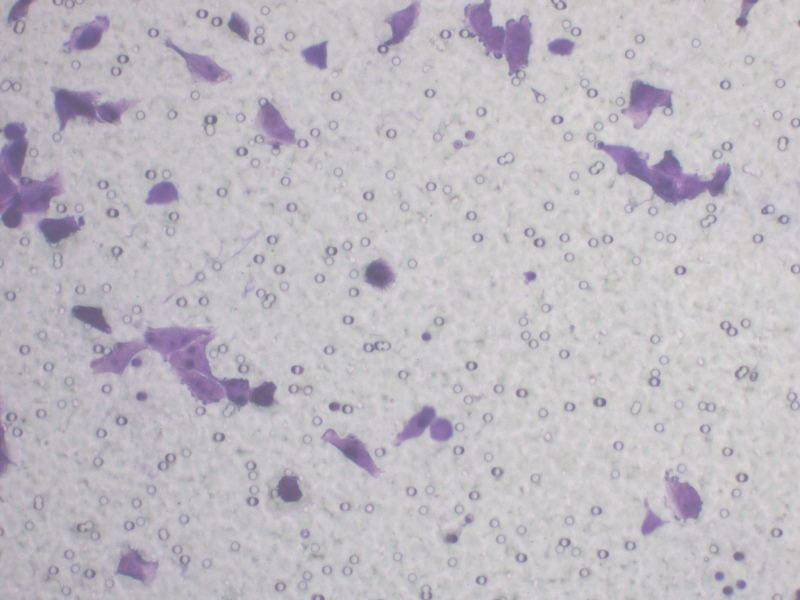

Supplement: Supplementary file 2 [file DataSheet_2.zip › data source-2/Figure 5/B/migration/143B + M- ELFN1-AS1 siRNA/2.jpg]

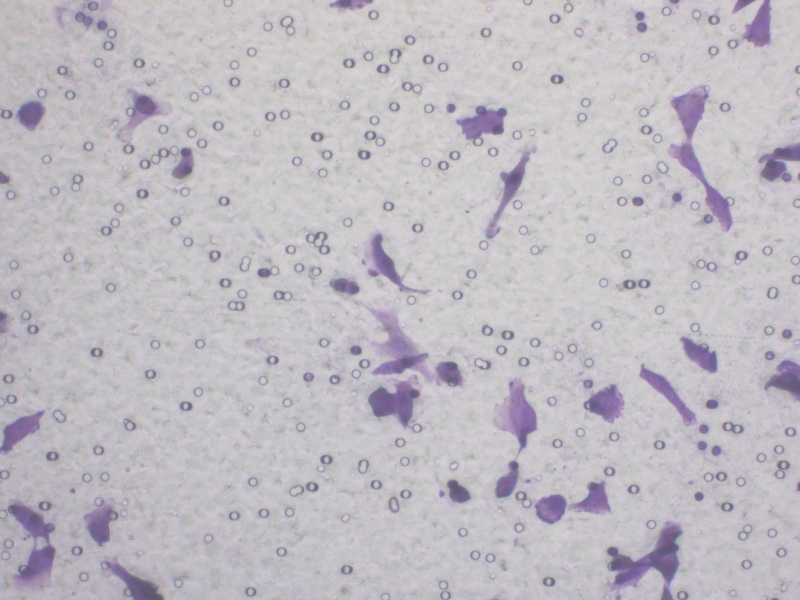

Supplement: Supplementary file 2 [file DataSheet_2.zip › data source-2/Figure 5/B/migration/143B + M- ELFN1-AS1 siRNA/3.jpg]

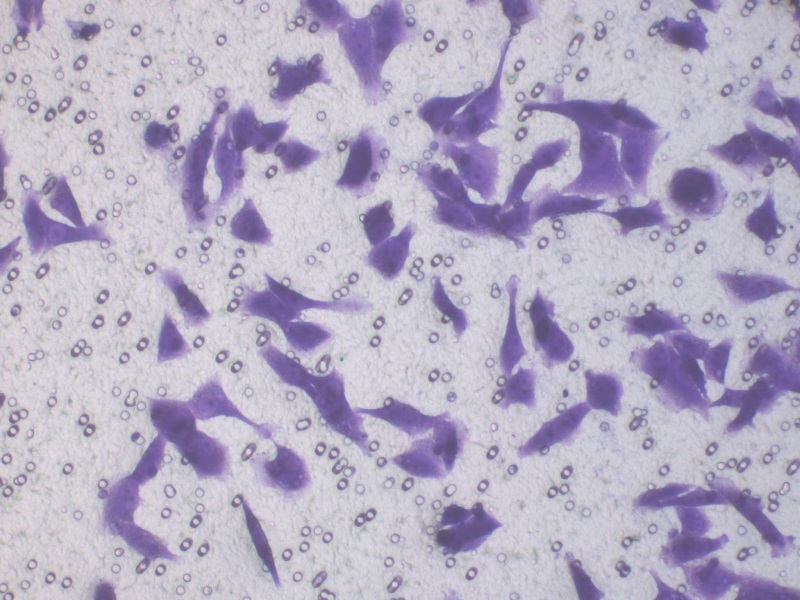

Supplement: Supplementary file 2 [file DataSheet_2.zip › data source-2/Figure 5/B/migration/143B + M-pcDNA3.1 ELFN1-AS1/1.jpg]

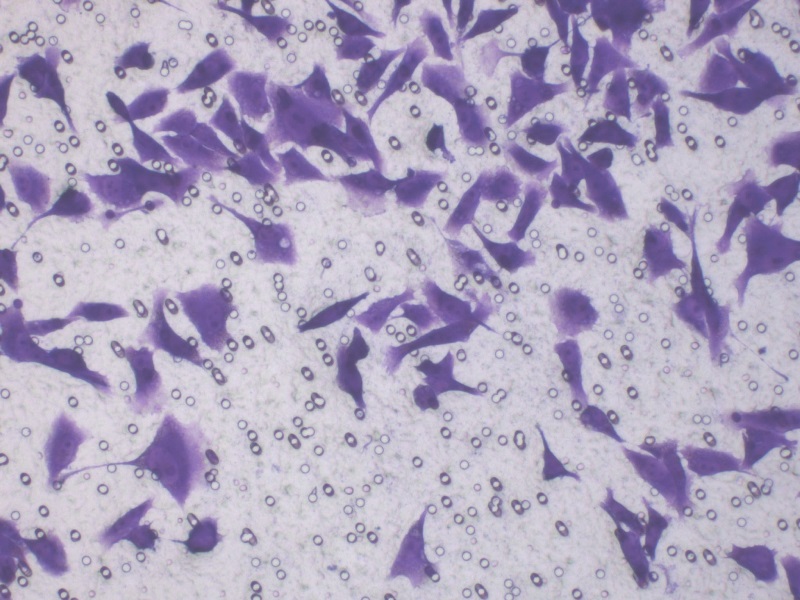

Supplement: Supplementary file 2 [file DataSheet_2.zip › data source-2/Figure 5/B/migration/143B + M-pcDNA3.1 ELFN1-AS1/2.jpg]

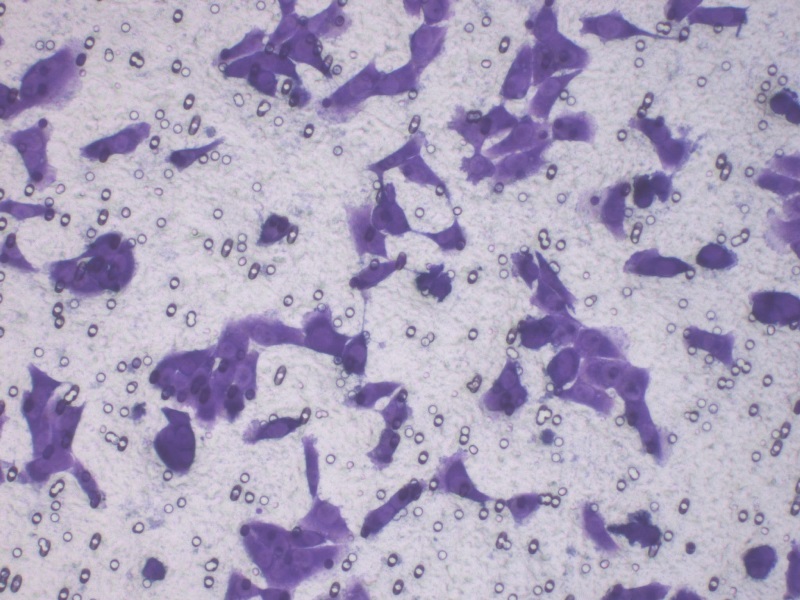

Supplement: Supplementary file 2 [file DataSheet_2.zip › data source-2/Figure 5/B/migration/143B + M-pcDNA3.1 ELFN1-AS1/3.jpg]

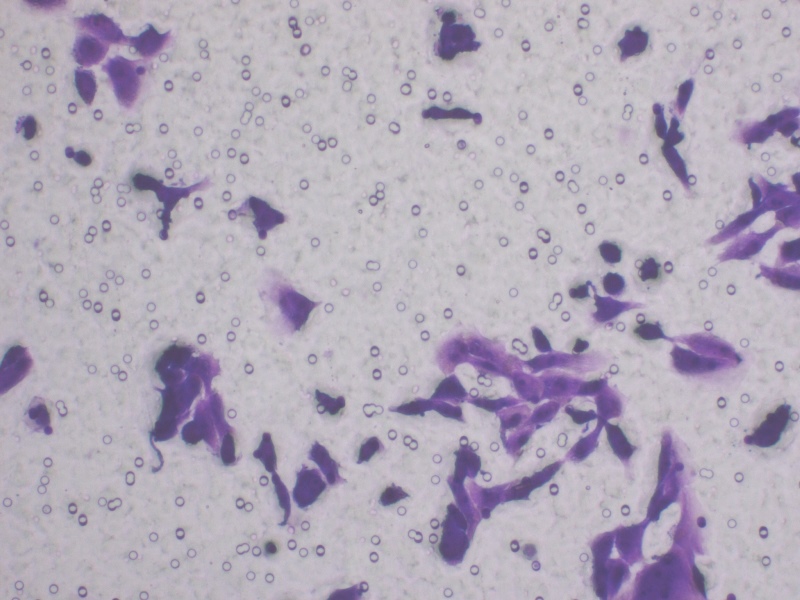

Supplement: Supplementary file 2 [file DataSheet_2.zip › data source-2/Figure 5/B/migration/143B + M-pcDNA3.1 NC/1.jpg]

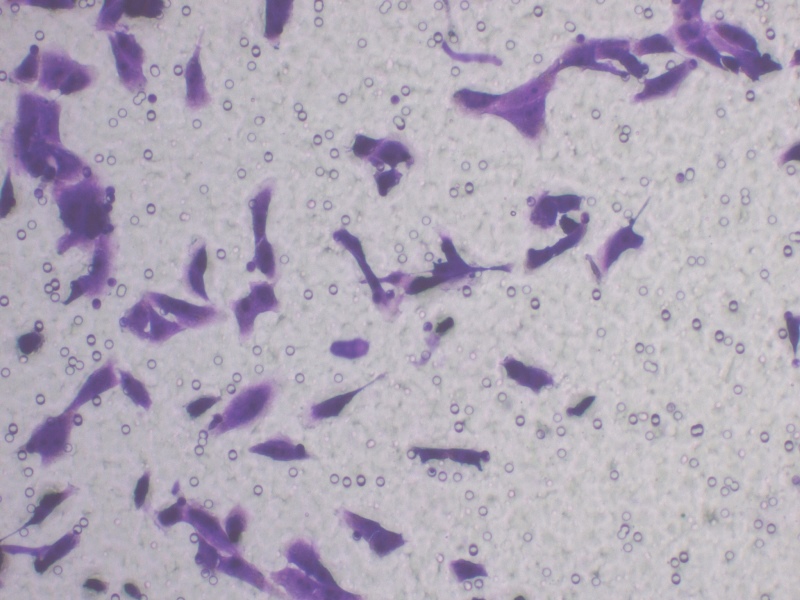

Supplement: Supplementary file 2 [file DataSheet_2.zip › data source-2/Figure 5/B/migration/143B + M-pcDNA3.1 NC/2.jpg]

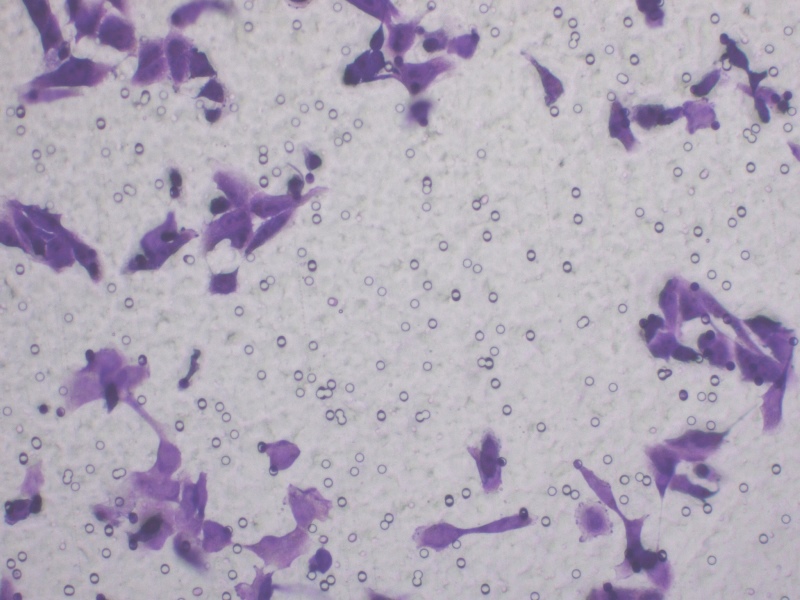

Supplement: Supplementary file 2 [file DataSheet_2.zip › data source-2/Figure 5/B/migration/143B + M-pcDNA3.1 NC/3.jpg]

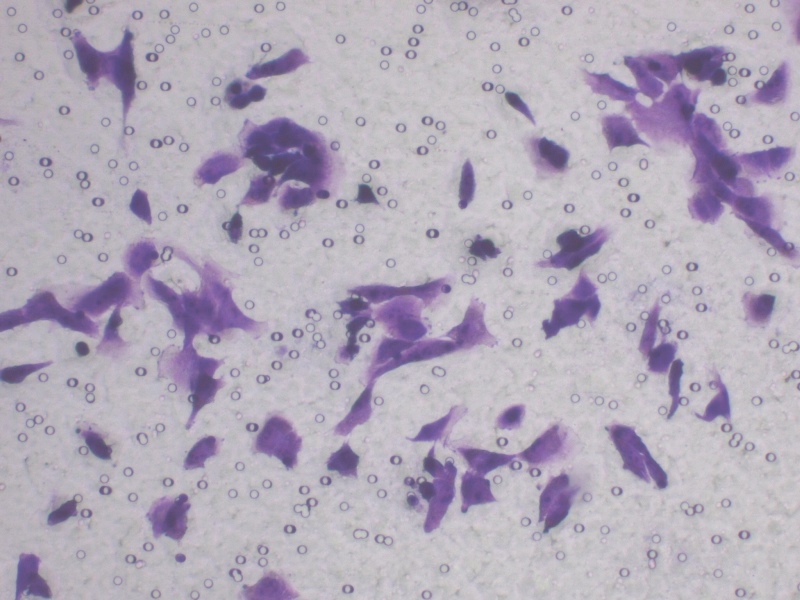

Supplement: Supplementary file 2 [file DataSheet_2.zip › data source-2/Figure 5/B/migration/143B + MsiRNA NC/1.jpg]

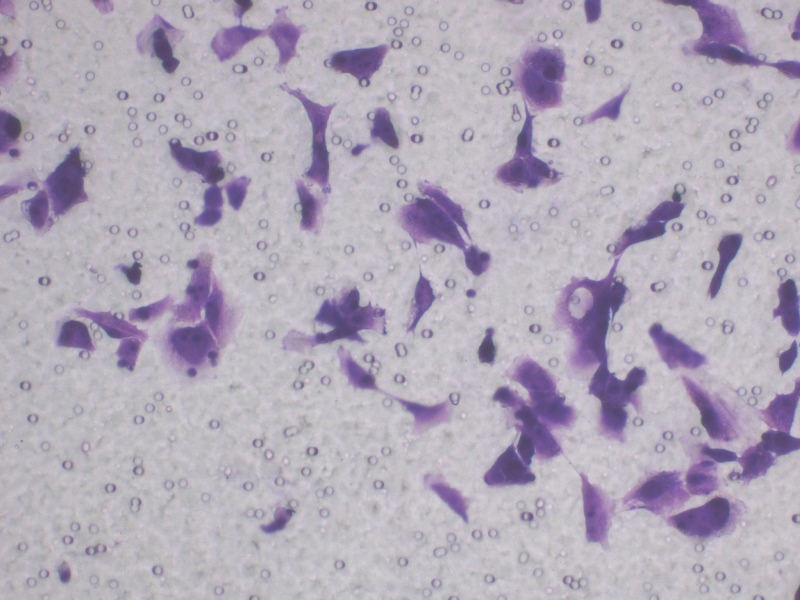

Supplement: Supplementary file 2 [file DataSheet_2.zip › data source-2/Figure 5/B/migration/143B + MsiRNA NC/2.jpg]

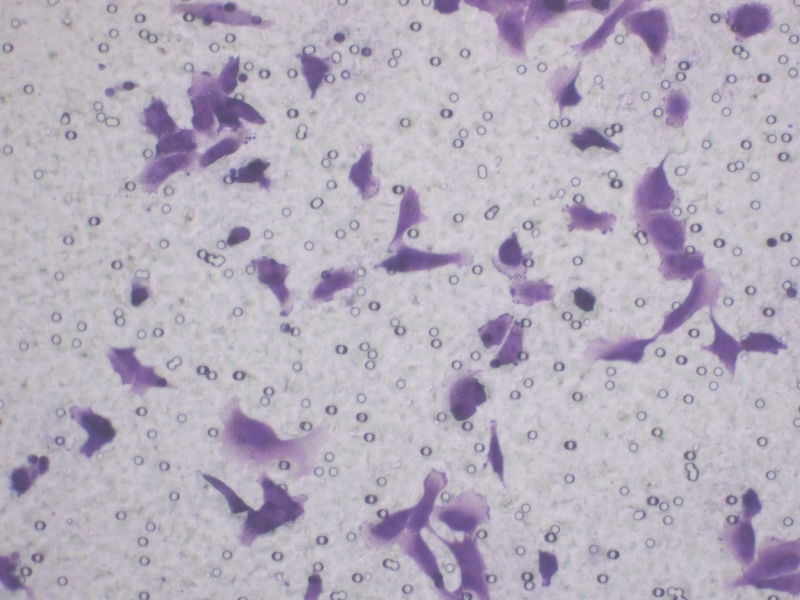

Supplement: Supplementary file 2 [file DataSheet_2.zip › data source-2/Figure 5/B/migration/143B + MsiRNA NC/3.jpg]

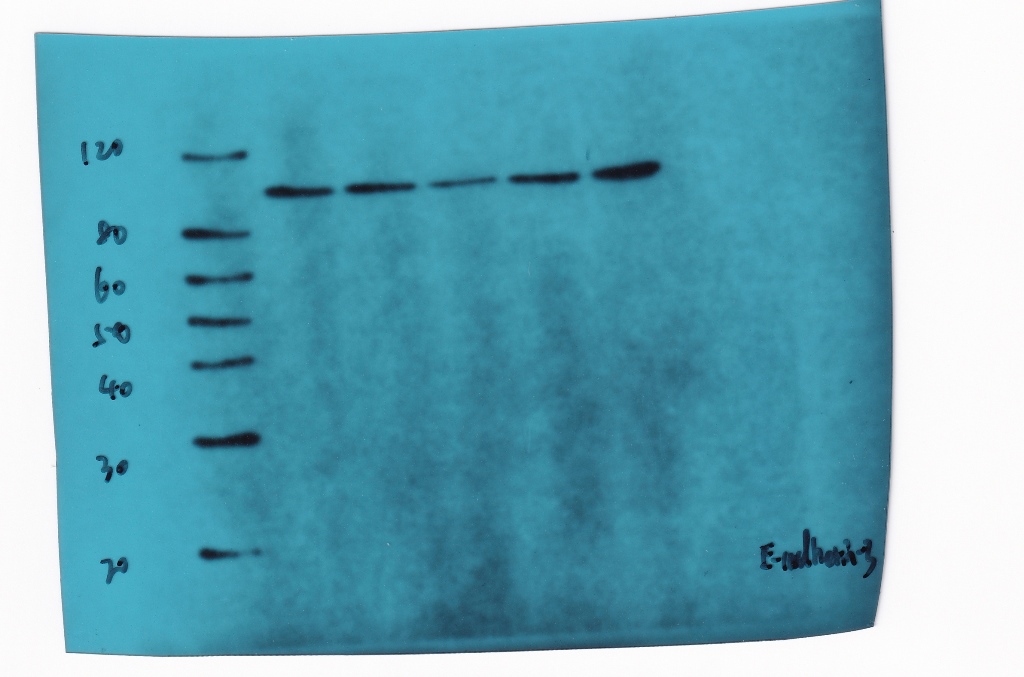

Supplement: Supplementary file 2 [file DataSheet_2.zip › data source-2/Figure 5/C/Figure 5C-E-cadherin.jpg]

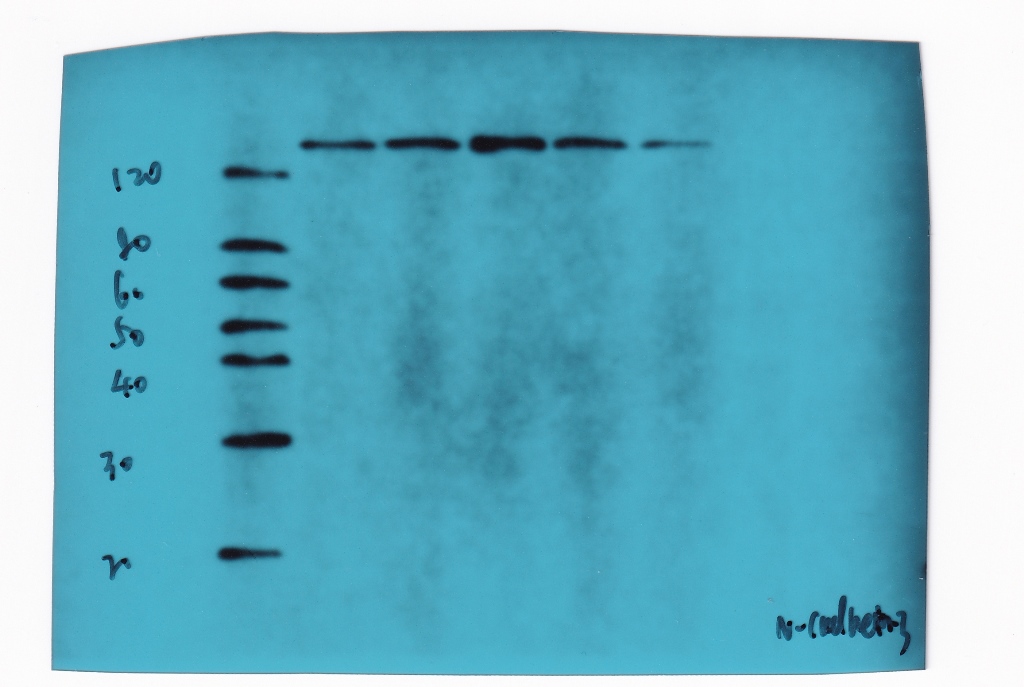

Supplement: Supplementary file 2 [file DataSheet_2.zip › data source-2/Figure 5/C/Figure 5C-N-cadherin.jpg]

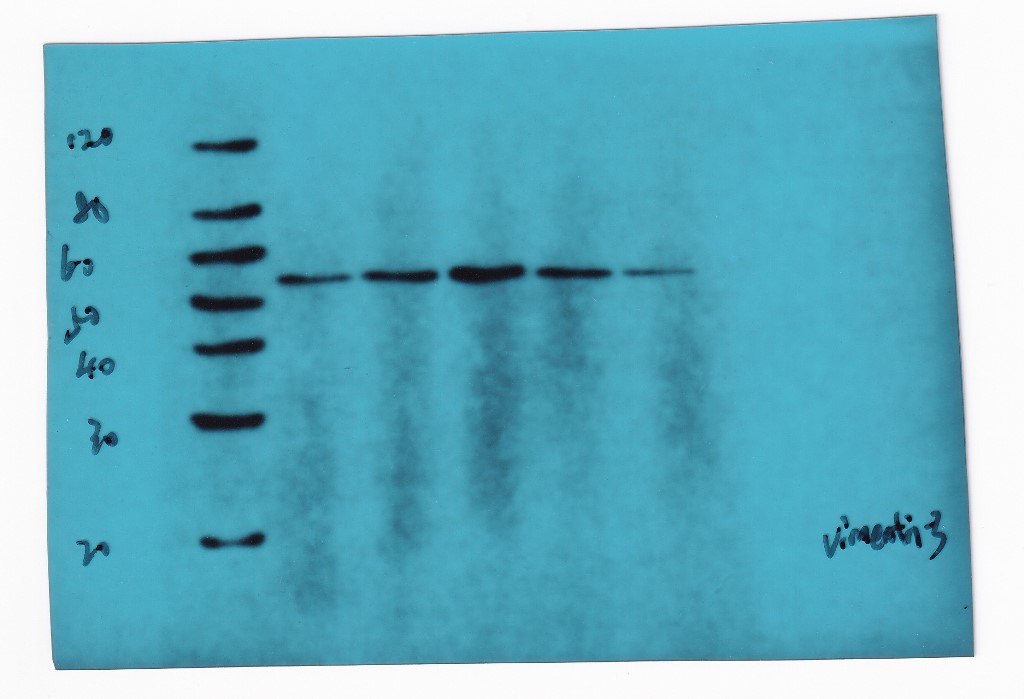

Supplement: Supplementary file 2 [file DataSheet_2.zip › data source-2/Figure 5/C/Figure 5C-Vimentin .jpg]

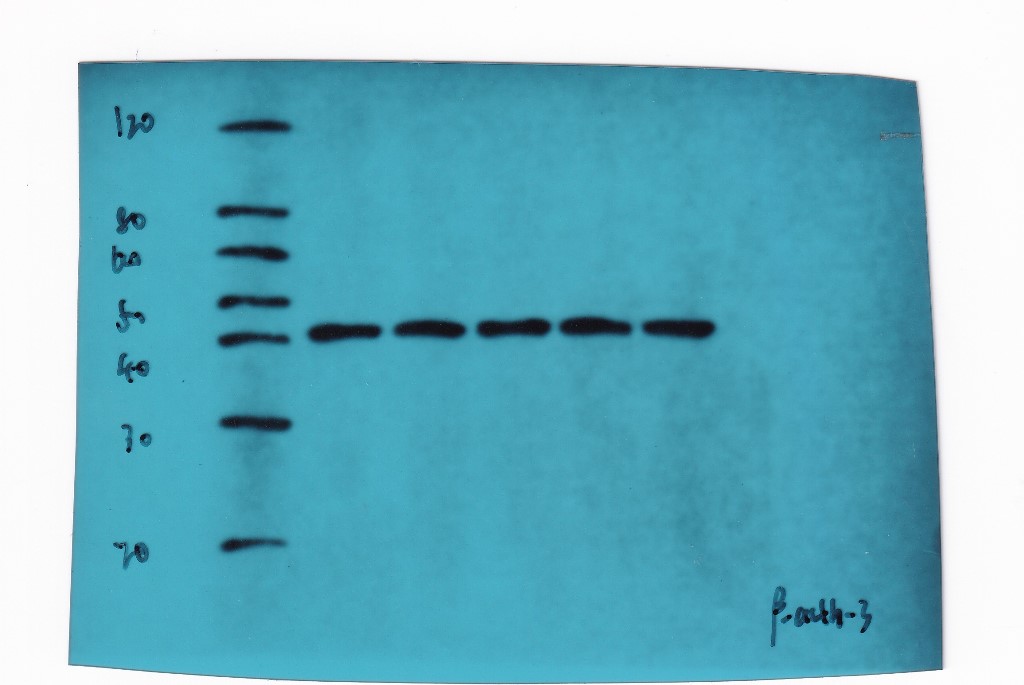

Supplement: Supplementary file 2 [file DataSheet_2.zip › data source-2/Figure 5/C/Figure 5C-a┬-actin.jpg]

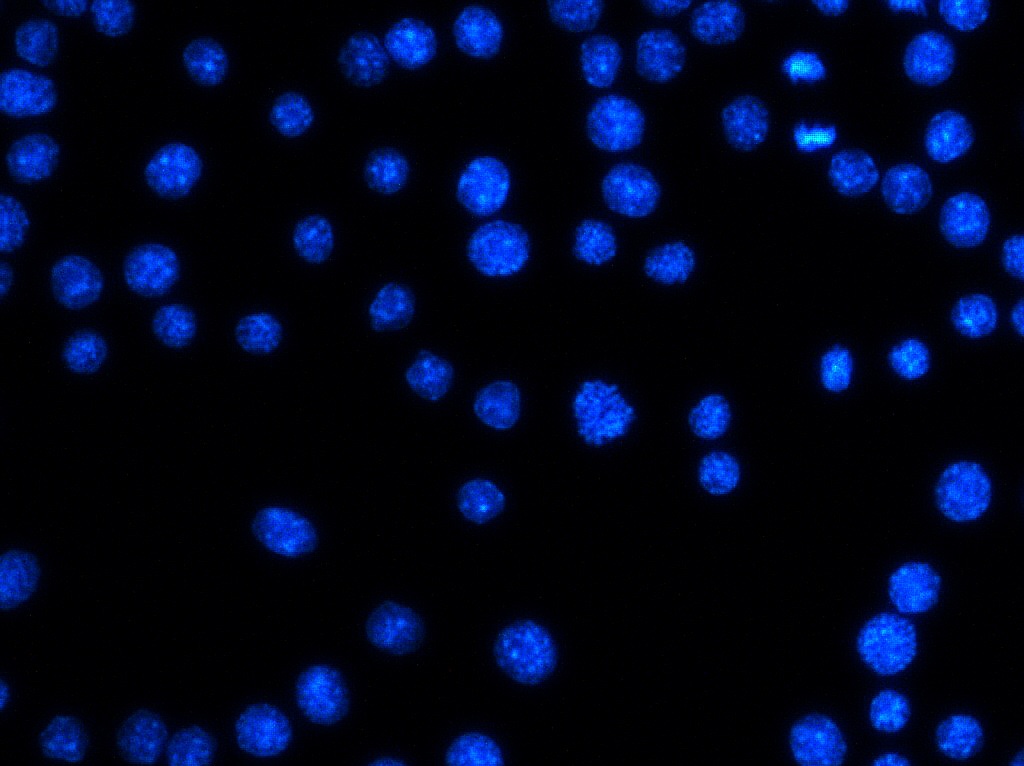

Supplement: Supplementary file 2 [file DataSheet_2.zip › data source-2/Figure 6/D/DAPI.jpg]

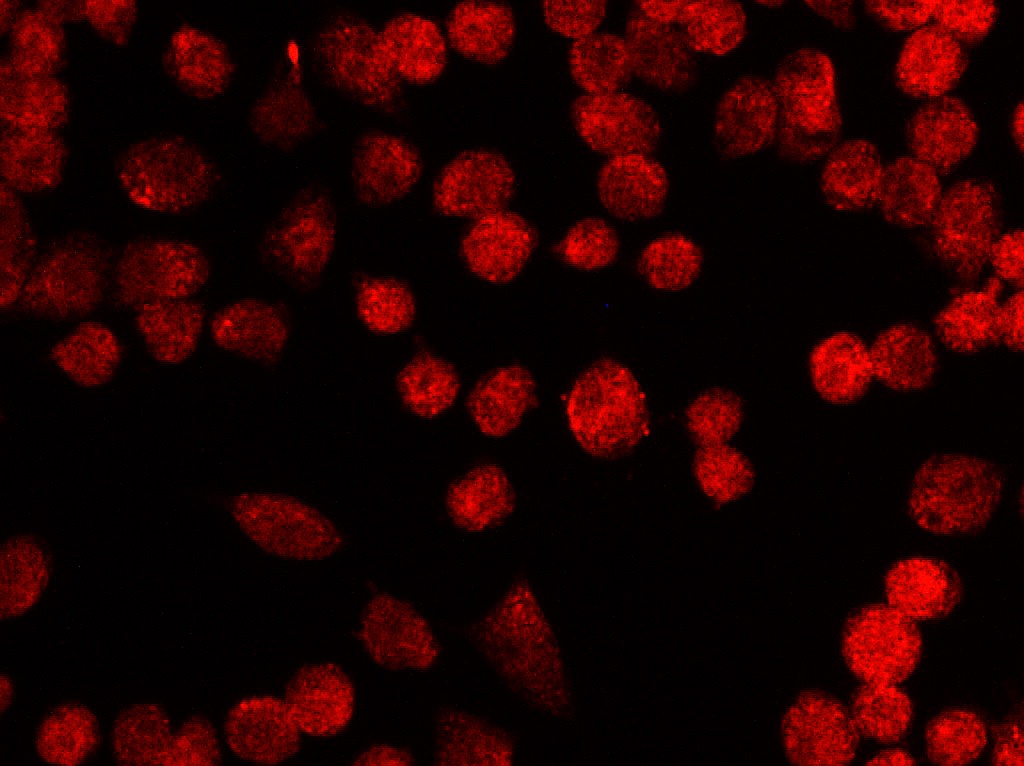

Supplement: Supplementary file 2 [file DataSheet_2.zip › data source-2/Figure 6/D/ELFN1-AS1.jpg]

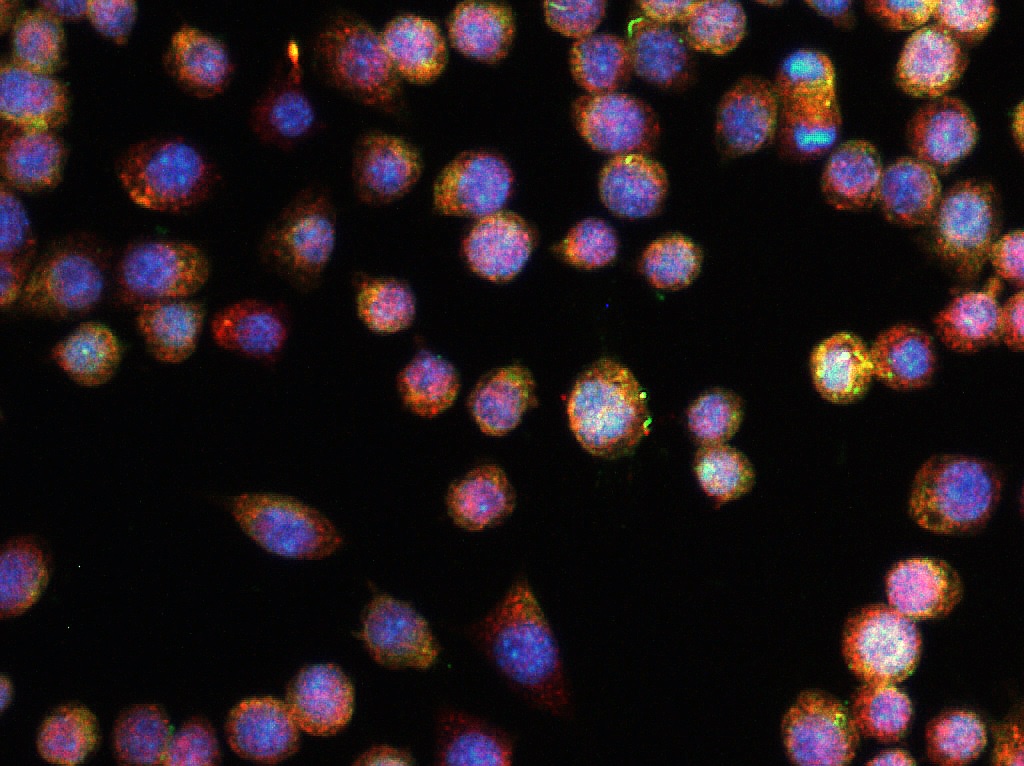

Supplement: Supplementary file 2 [file DataSheet_2.zip › data source-2/Figure 6/D/Merge.jpg]

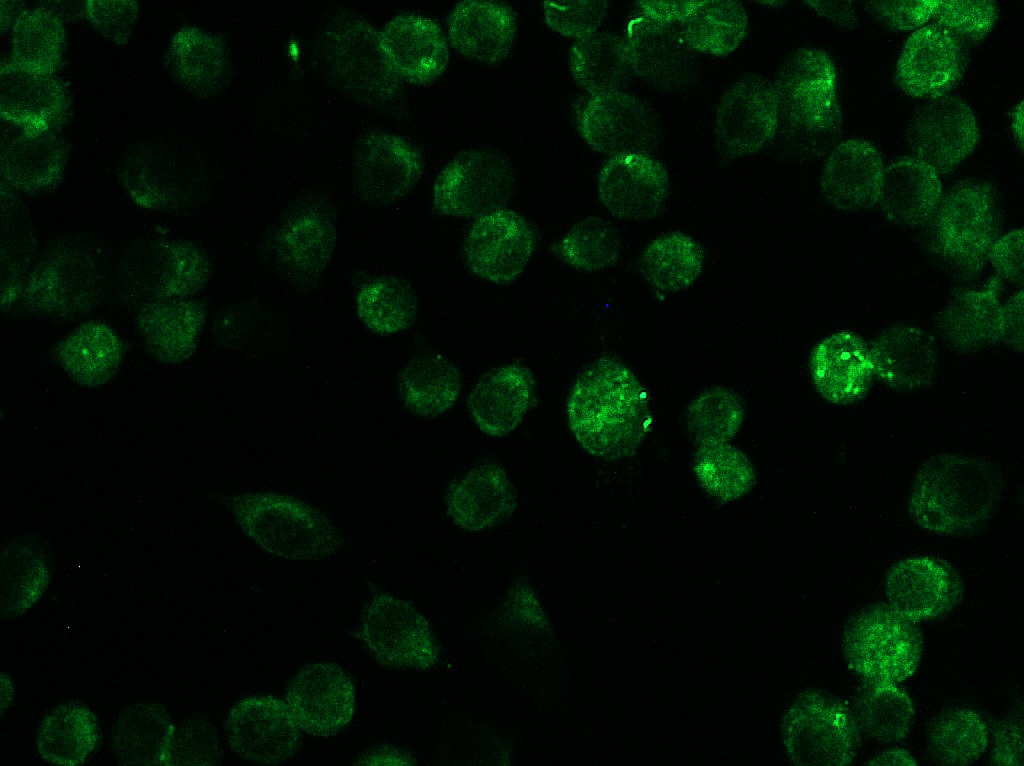

Supplement: Supplementary file 2 [file DataSheet_2.zip › data source-2/Figure 6/D/miR-138-5p.jpg]

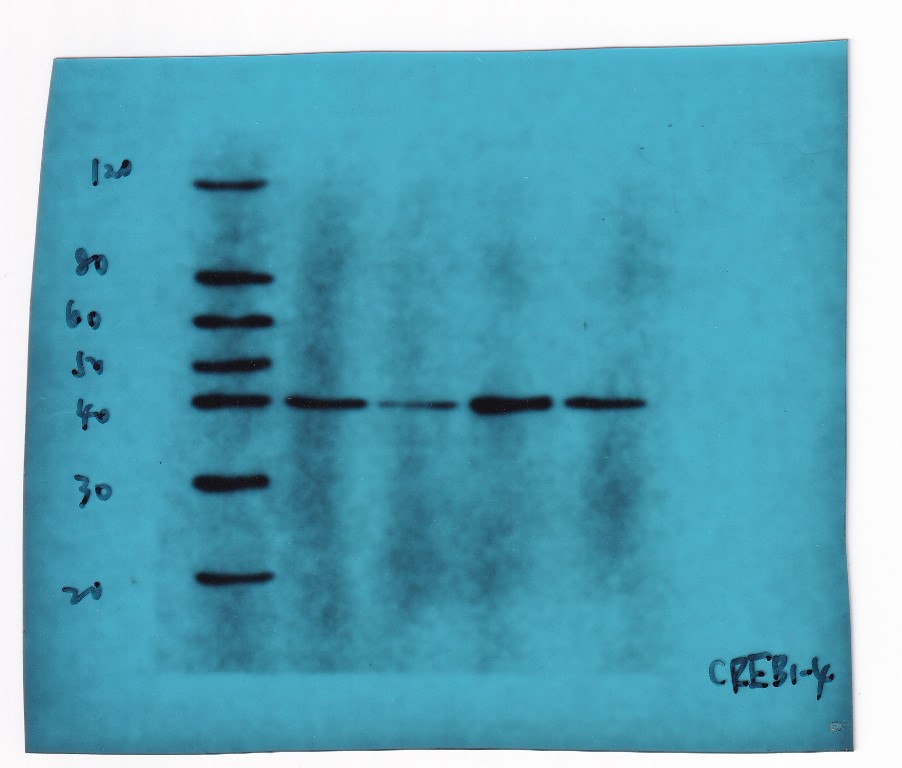

Supplement: Supplementary file 2 [file DataSheet_2.zip › data source-2/Figure 6/I/Figure 6I-CREB1.jpg]

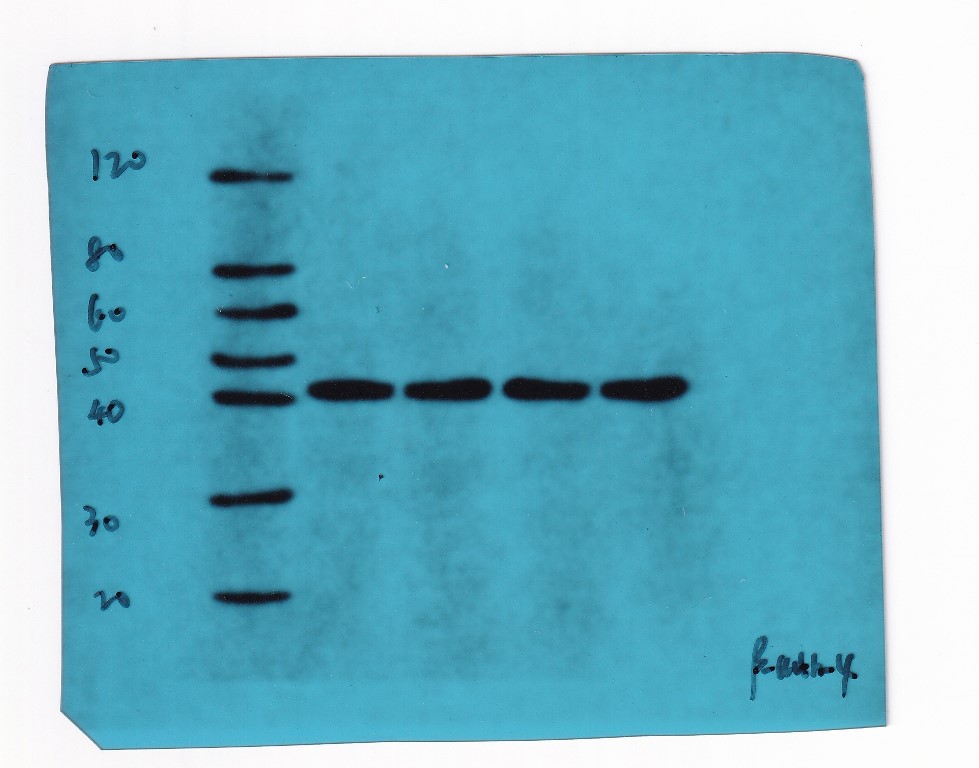

Supplement: Supplementary file 2 [file DataSheet_2.zip › data source-2/Figure 6/I/Figure 6I-a┬-actin.jpg]

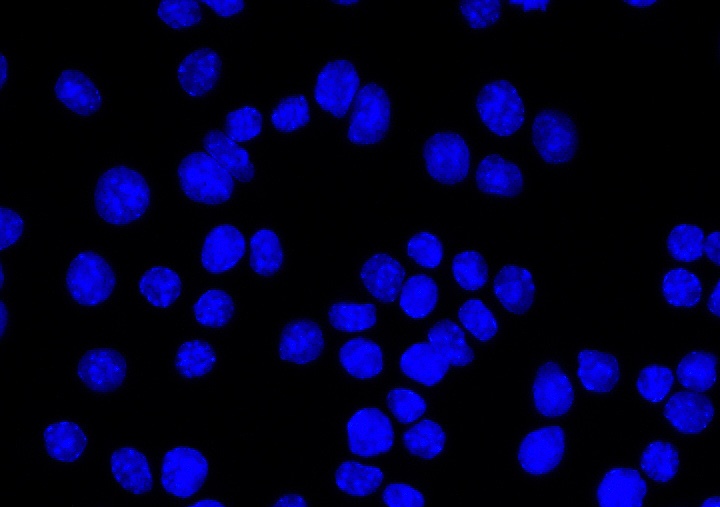

Supplement: Supplementary file 3 [file DataSheet_3.zip › data source-3/Figure 7/D/DAPI.jpg]

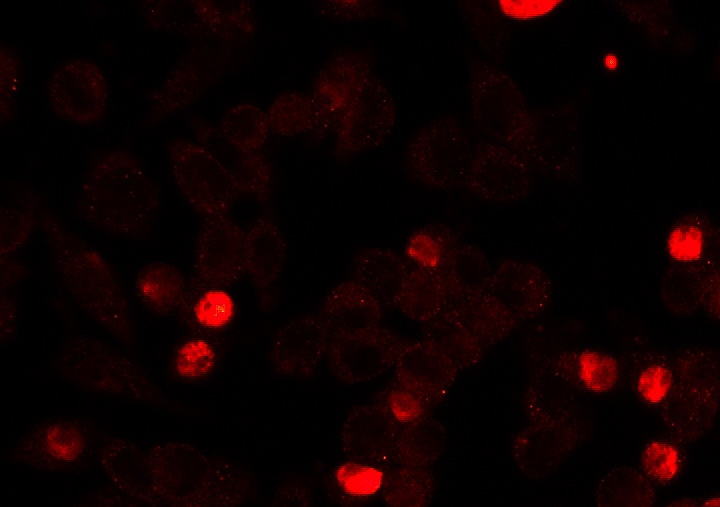

Supplement: Supplementary file 3 [file DataSheet_3.zip › data source-3/Figure 7/D/ELFN1-AS1.jpg]

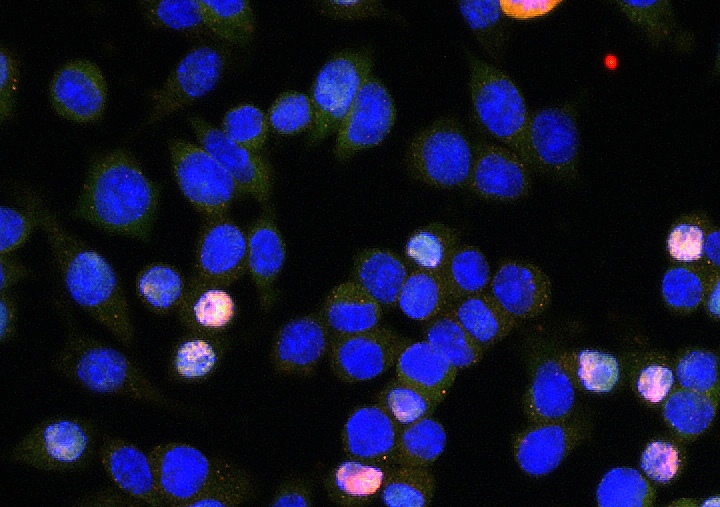

Supplement: Supplementary file 3 [file DataSheet_3.zip › data source-3/Figure 7/D/Merge.jpg]

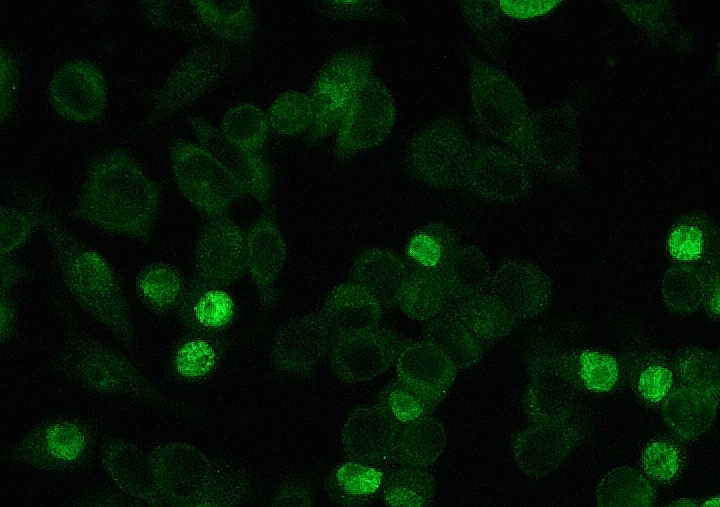

Supplement: Supplementary file 3 [file DataSheet_3.zip › data source-3/Figure 7/D/miR-1291.jpg]

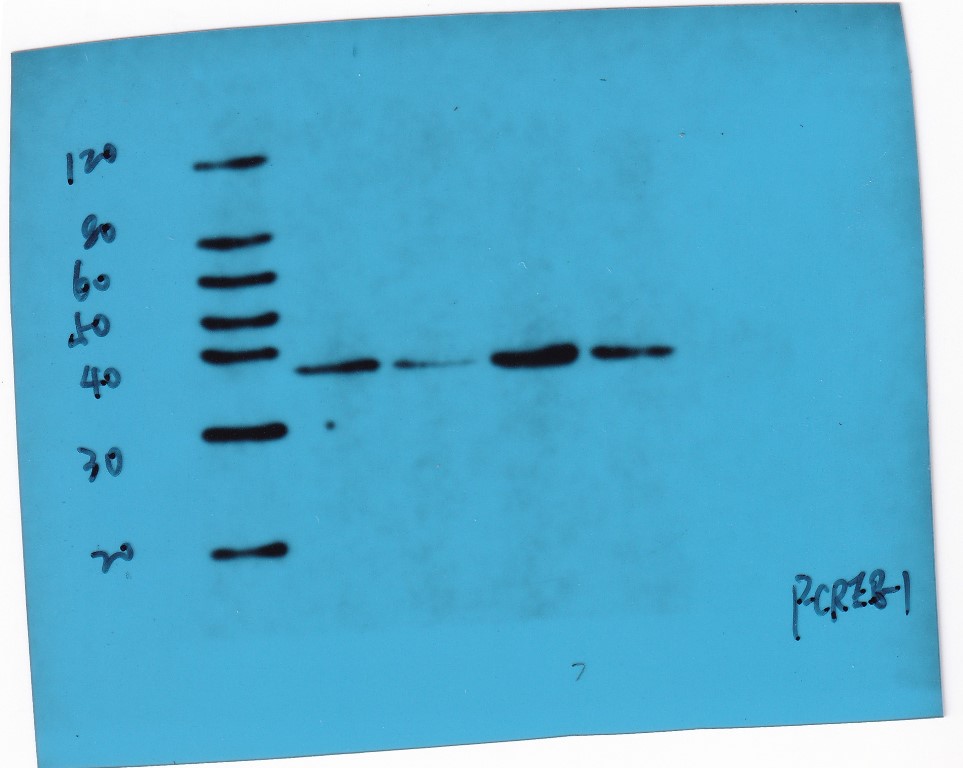

Supplement: Supplementary file 3 [file DataSheet_3.zip › data source-3/Figure 7/I/Figure 7I-CREB1.jpg]

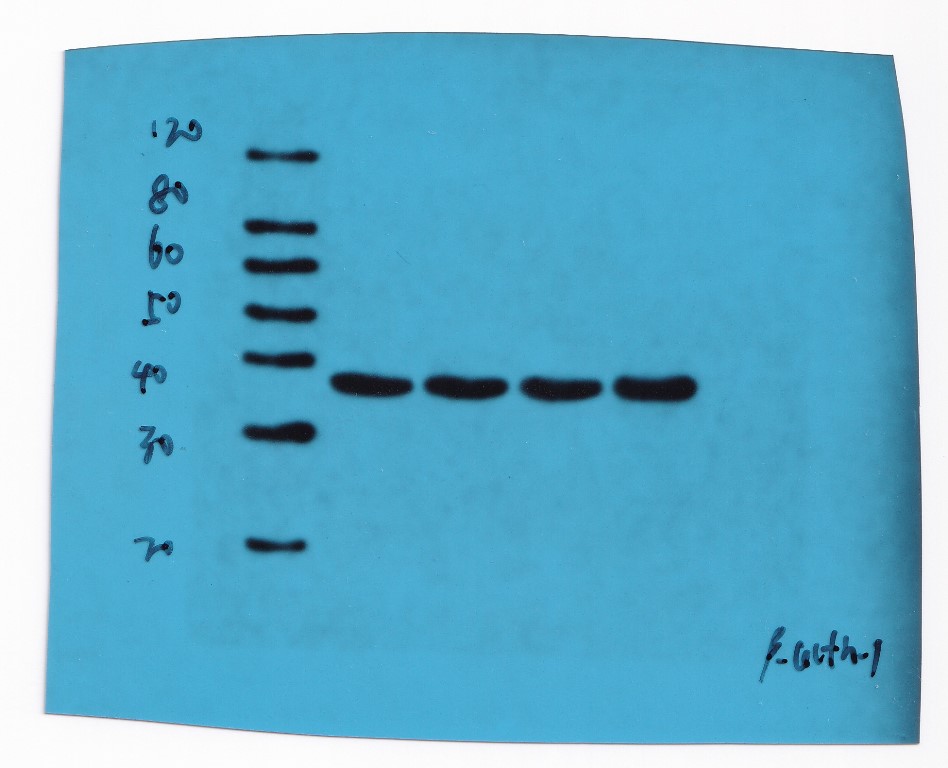

Supplement: Supplementary file 3 [file DataSheet_3.zip › data source-3/Figure 7/I/Figure 7I-a┬-actin.jpg]

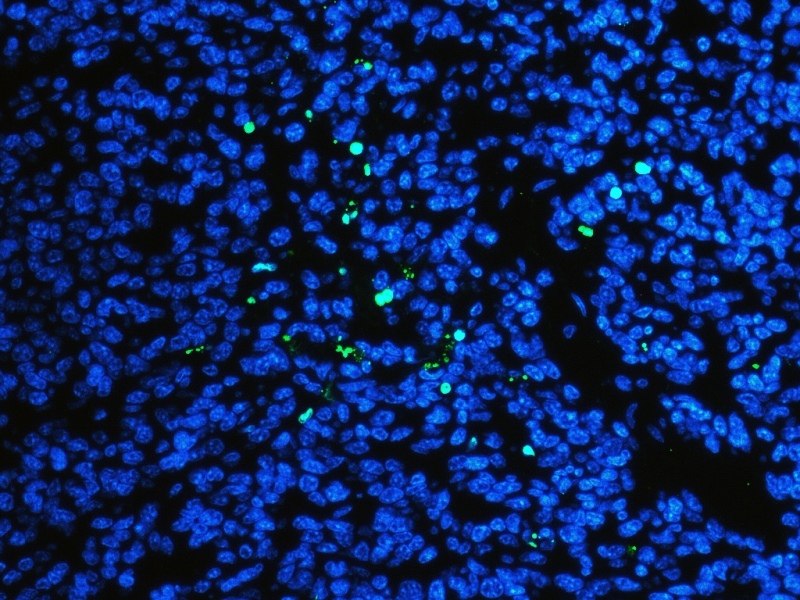

Supplement: Supplementary file 3 [file DataSheet_3.zip › data source-3/Figure 8/C/A6 400-1+2.JPG]

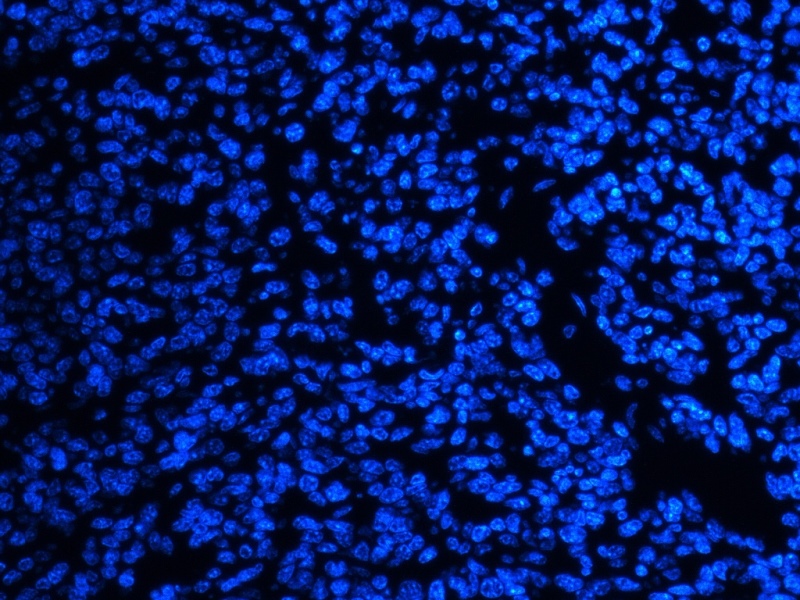

Supplement: Supplementary file 3 [file DataSheet_3.zip › data source-3/Figure 8/C/A6 400-1.jpg]

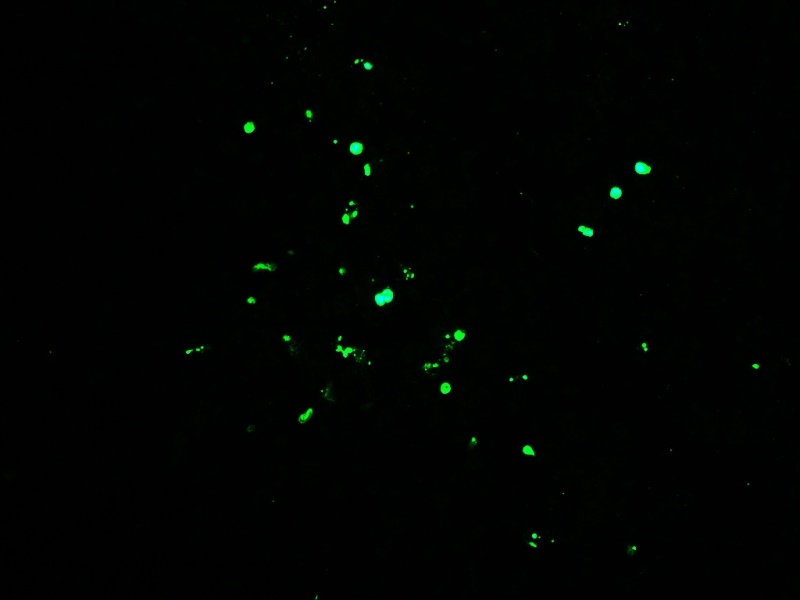

Supplement: Supplementary file 3 [file DataSheet_3.zip › data source-3/Figure 8/C/A6 400-2.jpg]

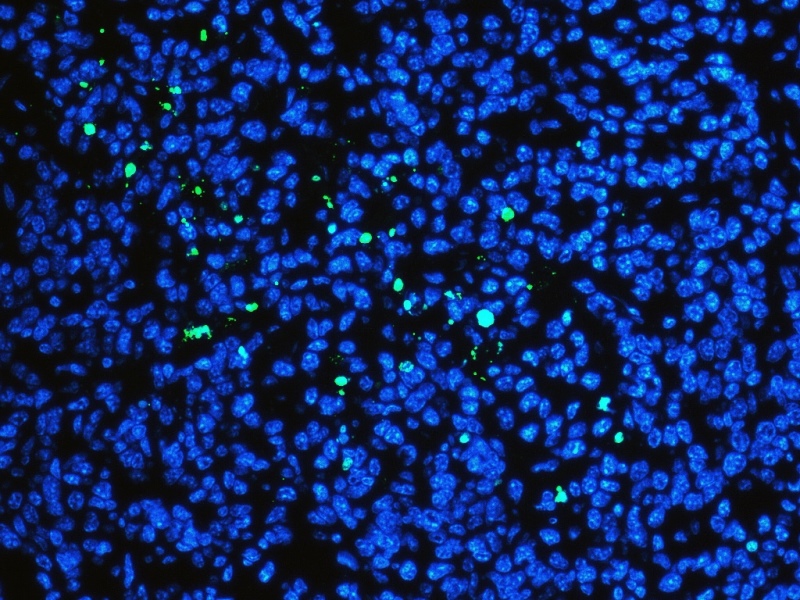

Supplement: Supplementary file 3 [file DataSheet_3.zip › data source-3/Figure 8/C/B6 400-1+2.JPG]

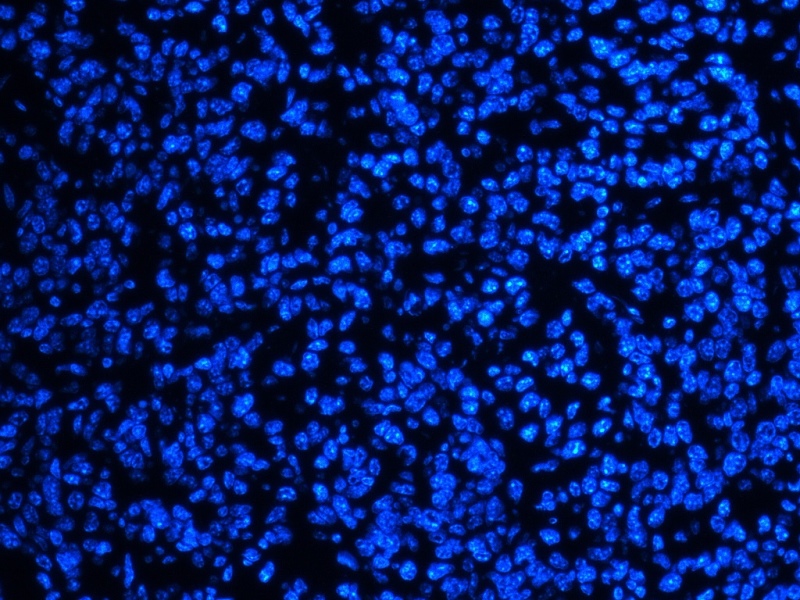

Supplement: Supplementary file 3 [file DataSheet_3.zip › data source-3/Figure 8/C/B6 400-1.jpg]

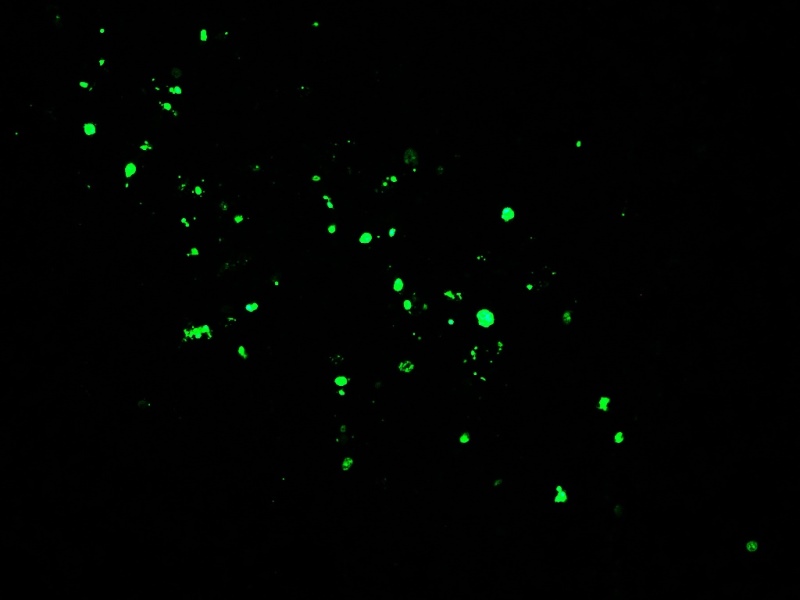

Supplement: Supplementary file 3 [file DataSheet_3.zip › data source-3/Figure 8/C/B6 400-2.jpg]

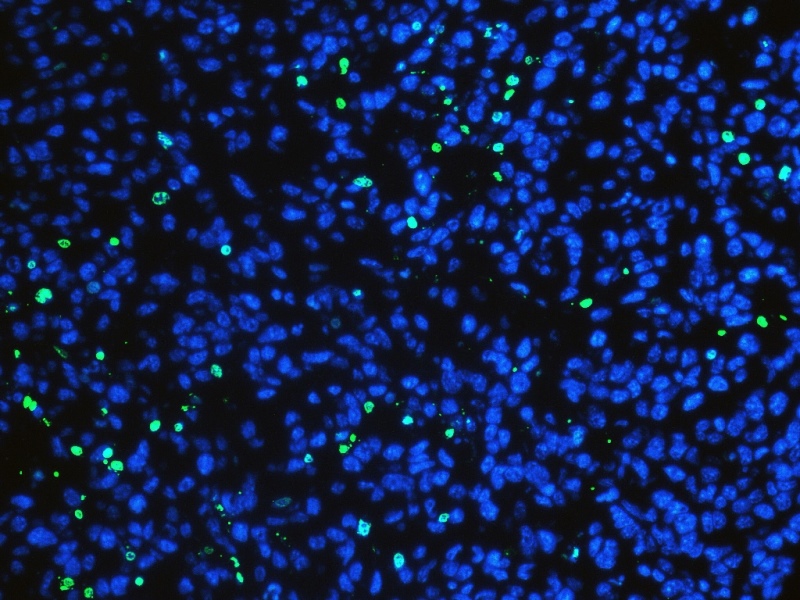

Supplement: Supplementary file 3 [file DataSheet_3.zip › data source-3/Figure 8/C/C6 400-1+2.JPG]

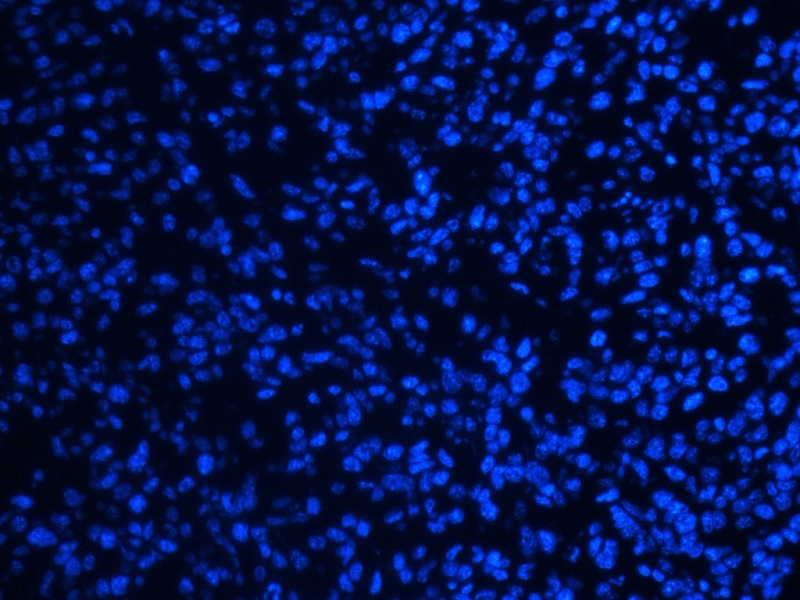

Supplement: Supplementary file 3 [file DataSheet_3.zip › data source-3/Figure 8/C/C6 400-1.jpg]

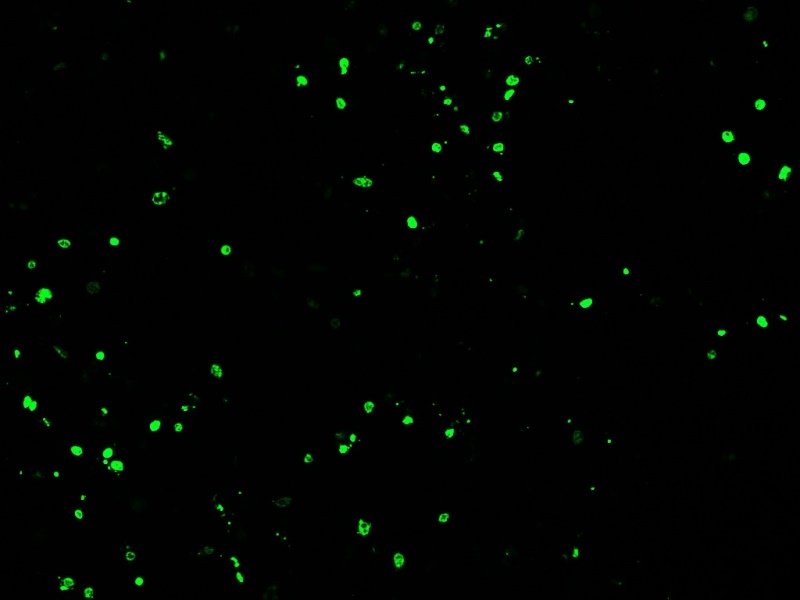

Supplement: Supplementary file 3 [file DataSheet_3.zip › data source-3/Figure 8/C/C6 400-2.jpg]

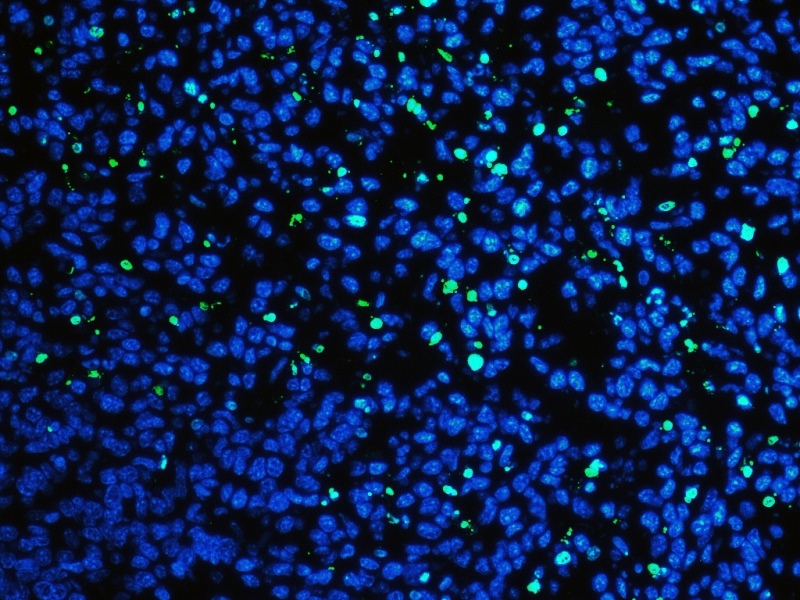

Supplement: Supplementary file 3 [file DataSheet_3.zip › data source-3/Figure 8/C/D6 400-1+2.JPG]

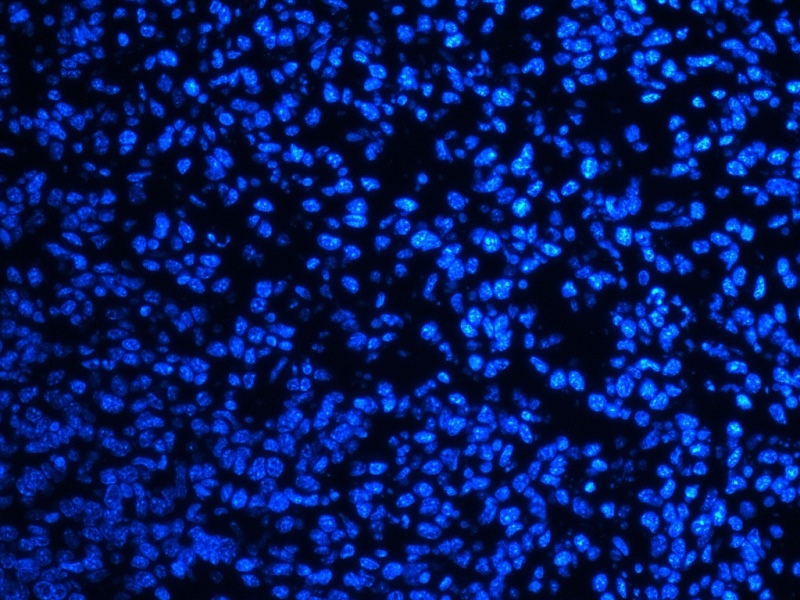

Supplement: Supplementary file 3 [file DataSheet_3.zip › data source-3/Figure 8/C/D6 400-1.jpg]

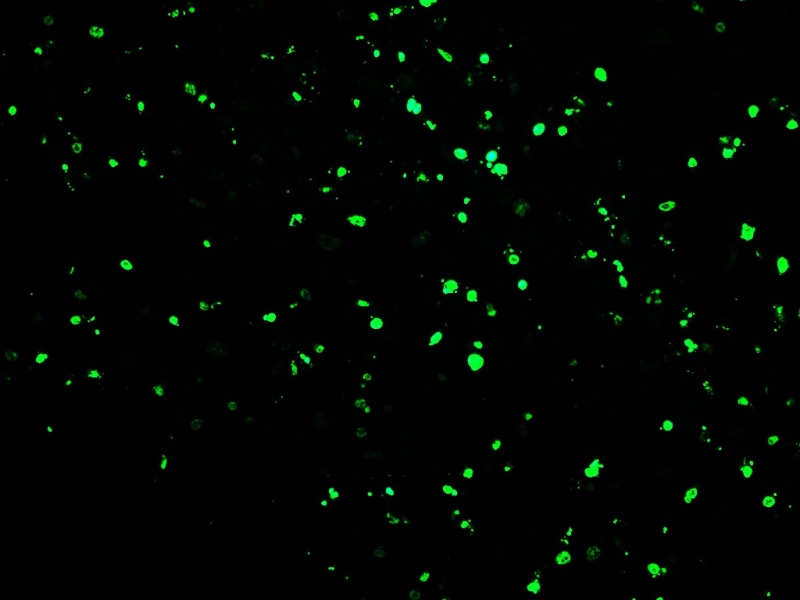

Supplement: Supplementary file 3 [file DataSheet_3.zip › data source-3/Figure 8/C/D6 400-2.jpg]

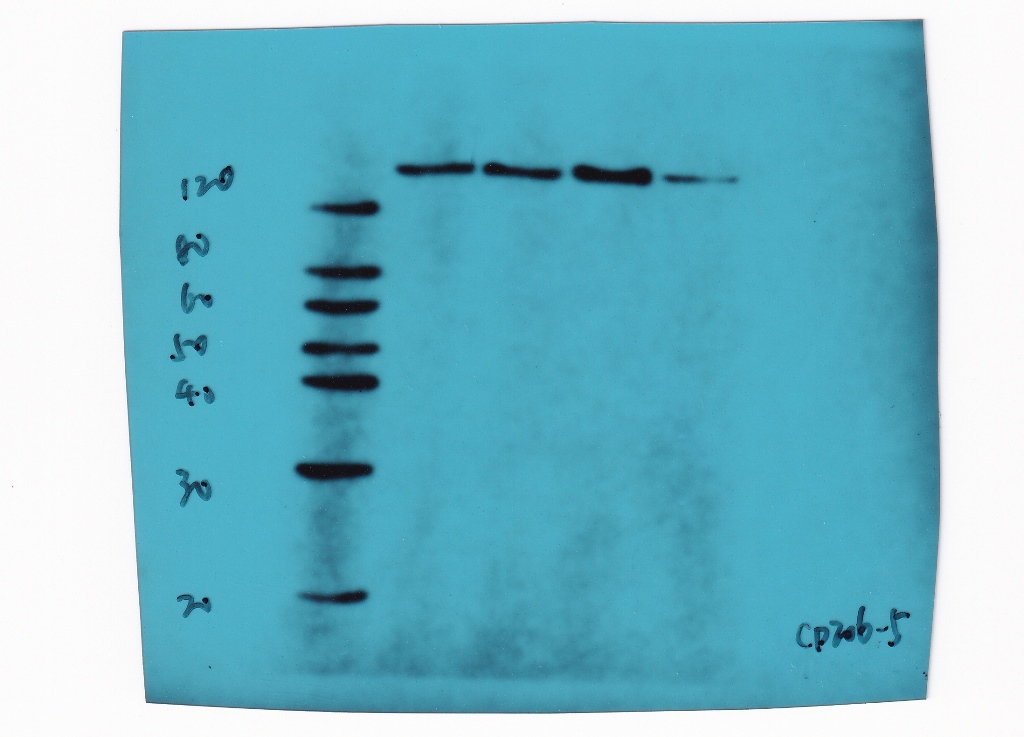

Supplement: Supplementary file 3 [file DataSheet_3.zip › data source-3/Figure 8/D/Figure 8D-CD206.jpg]

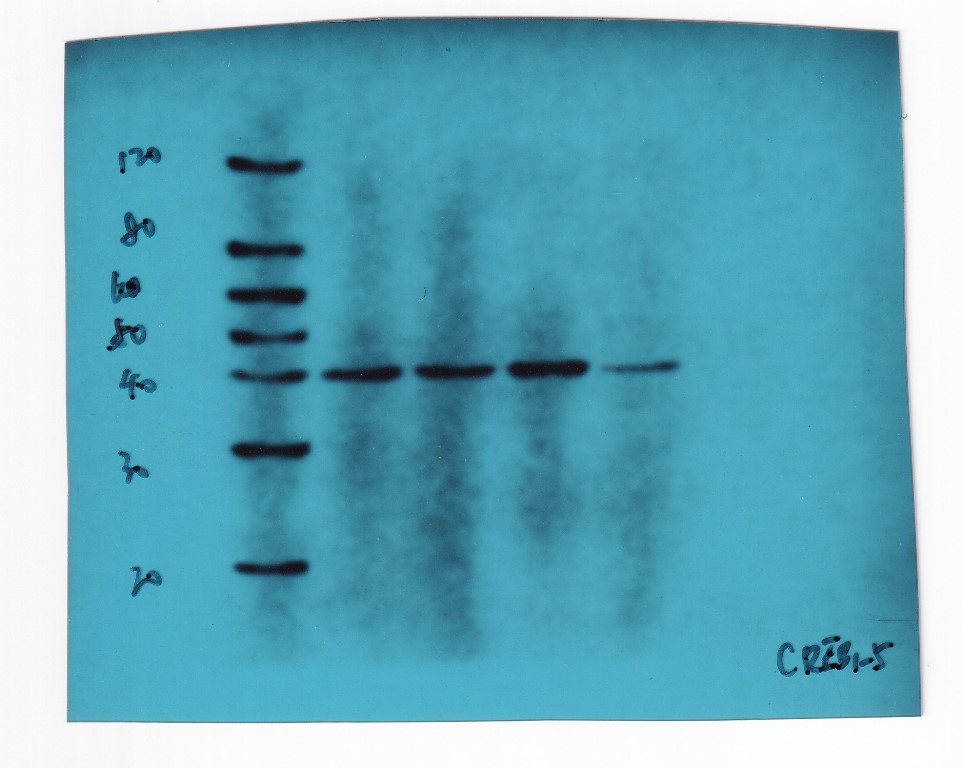

Supplement: Supplementary file 3 [file DataSheet_3.zip › data source-3/Figure 8/D/Figure 8D-CREB1.jpg]

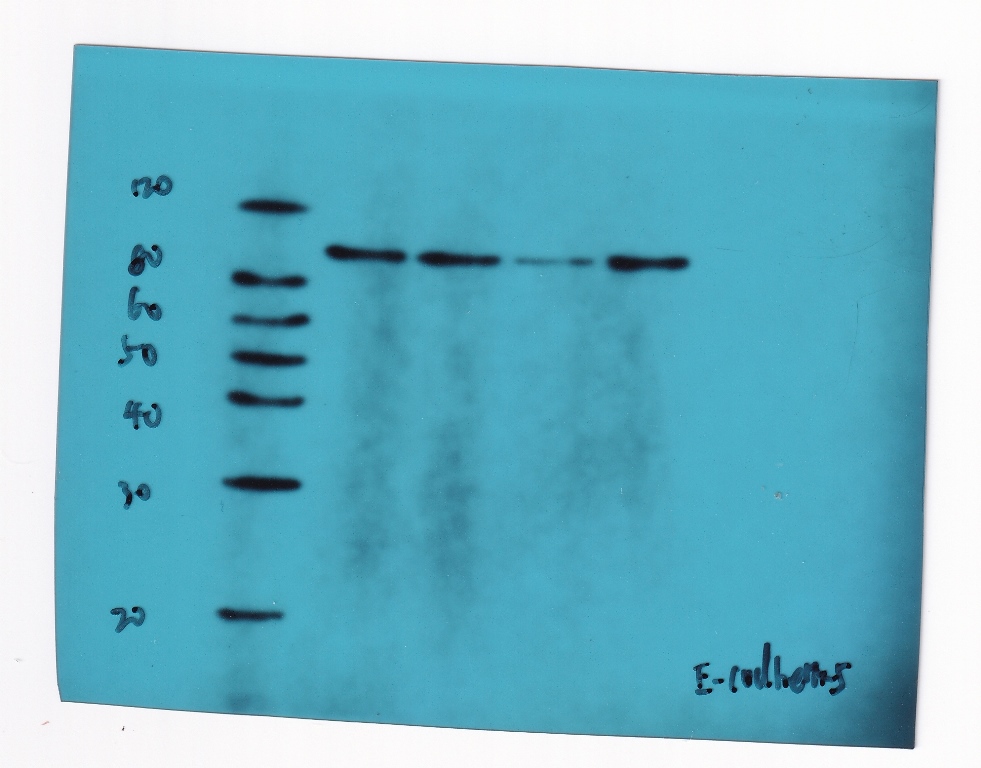

Supplement: Supplementary file 3 [file DataSheet_3.zip › data source-3/Figure 8/D/Figure 8D-E-cadherin.jpg]

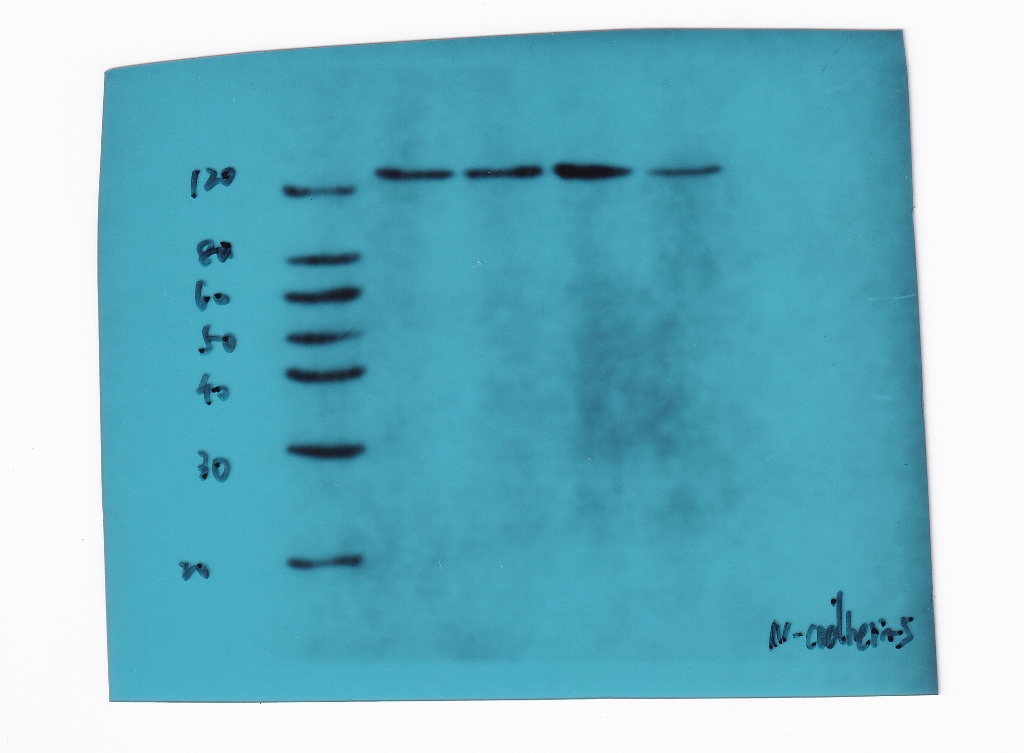

Supplement: Supplementary file 3 [file DataSheet_3.zip › data source-3/Figure 8/D/Figure 8D-N-cadherin.jpg]

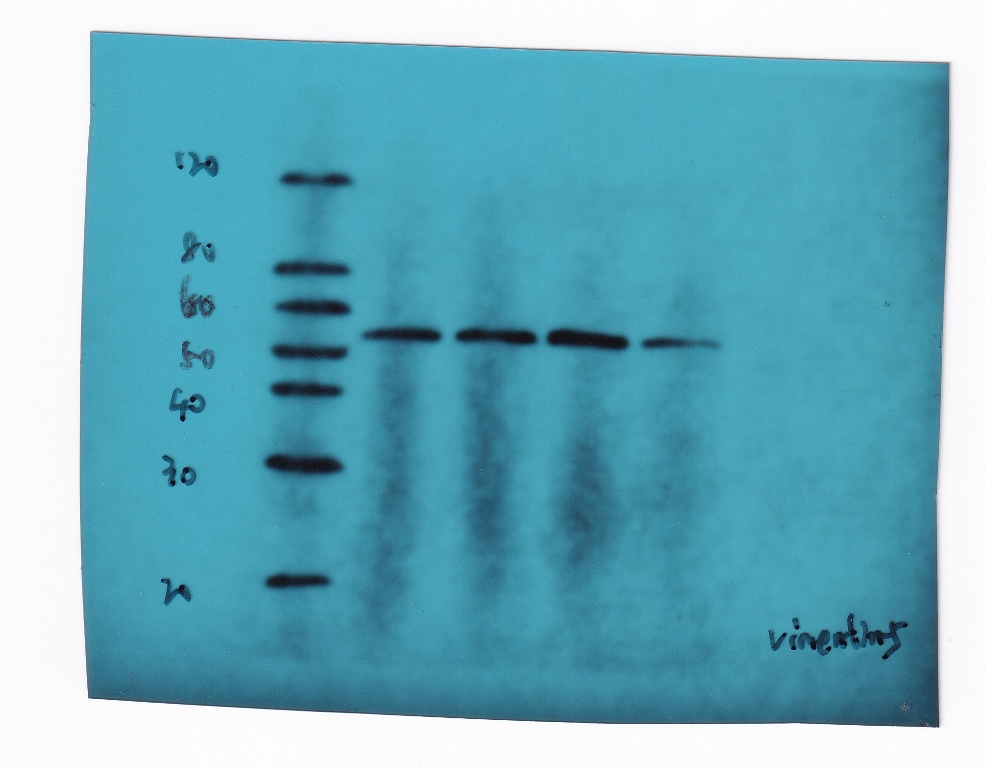

Supplement: Supplementary file 3 [file DataSheet_3.zip › data source-3/Figure 8/D/Figure 8D-Vimentin.jpg]

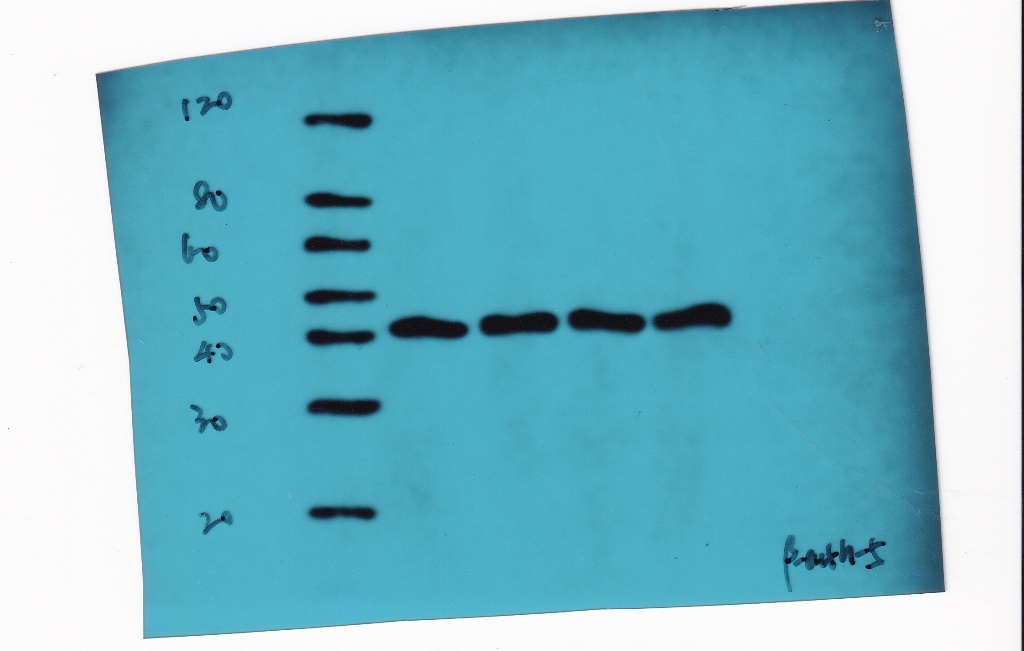

Supplement: Supplementary file 3 [file DataSheet_3.zip › data source-3/Figure 8/D/Figure 8D-a┬-actin.jpg]

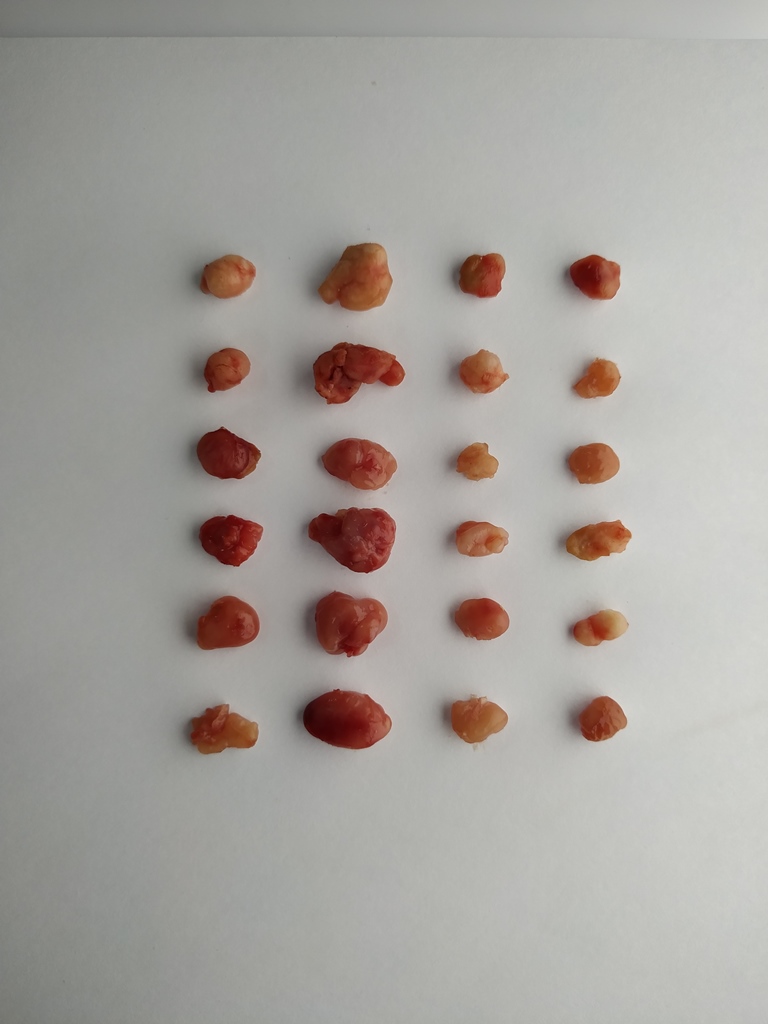

Supplement: Supplementary file 3 [file DataSheet_3.zip › data source-3/Figure 8/Tumor picture.jpg]
