# Supplementary figures and images for: Osteosarcoma Cell-Derived Exosomal ELFN1-AS1 Mediates Macrophage M2 Polarization via Sponging miR-138-5p and miR-1291 to Promote the Tumorgenesis of Osteosarcoma (part 1 of 2)
Source: Front Oncol. 2022 Jun 17;12:881022. doi: 10.3389/fonc.2022.881022 (PMC9248260; doi:10.3389/fonc.2022.881022)

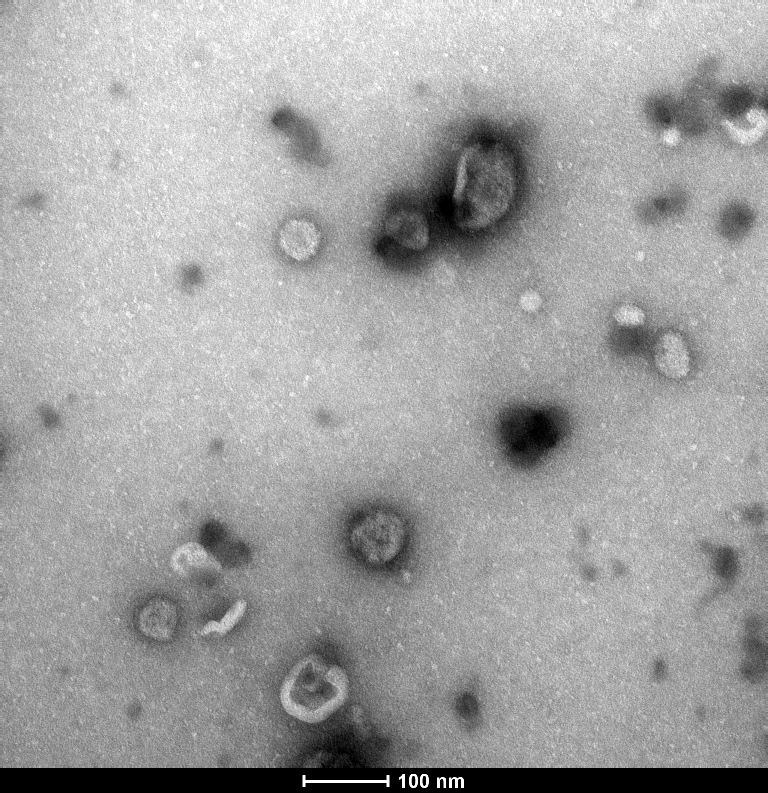

Supplement: Supplementary Figure 1 — The level of ELFN1-AS1 is upregulated in SARC tissues. [file DataSheet_1.zip › Figure 4/A/143BELFN1-AS1 siRNA1-Exo.tif]

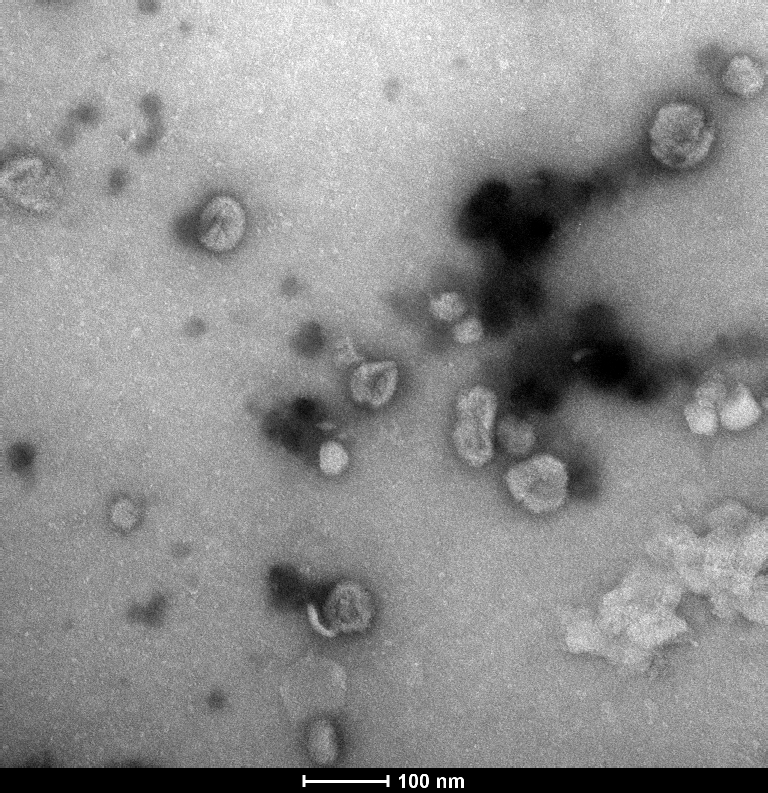

Supplement: Supplementary Figure 1 — The level of ELFN1-AS1 is upregulated in SARC tissues. [file DataSheet_1.zip › Figure 4/A/143BpcDNA3.1-ELFN1-AS1-Exo.tif]

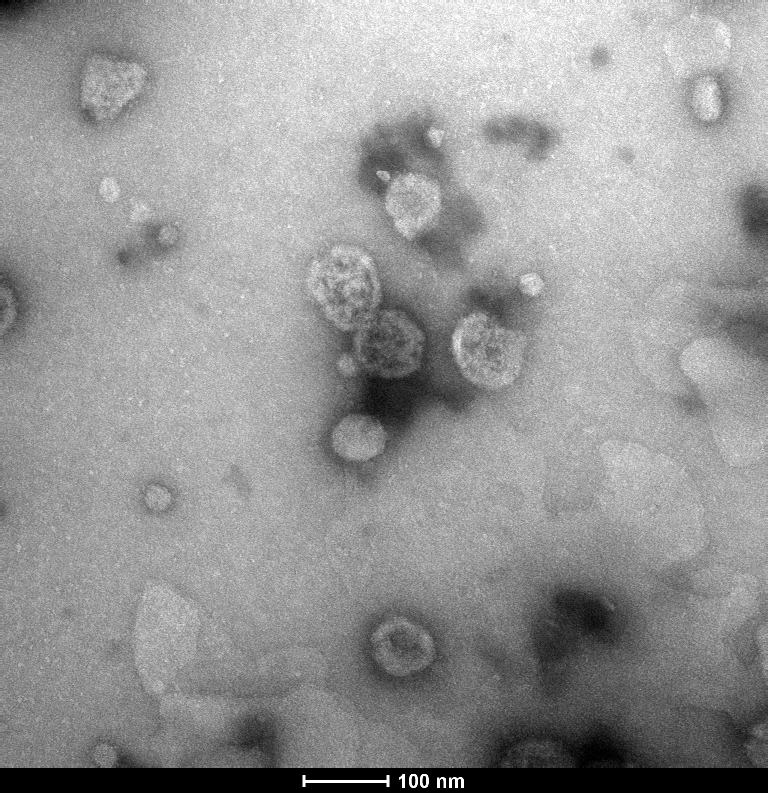

Supplement: Supplementary Figure 1 — The level of ELFN1-AS1 is upregulated in SARC tissues. [file DataSheet_1.zip › Figure 4/A/143BpcDNA3.1-Exo.tif]

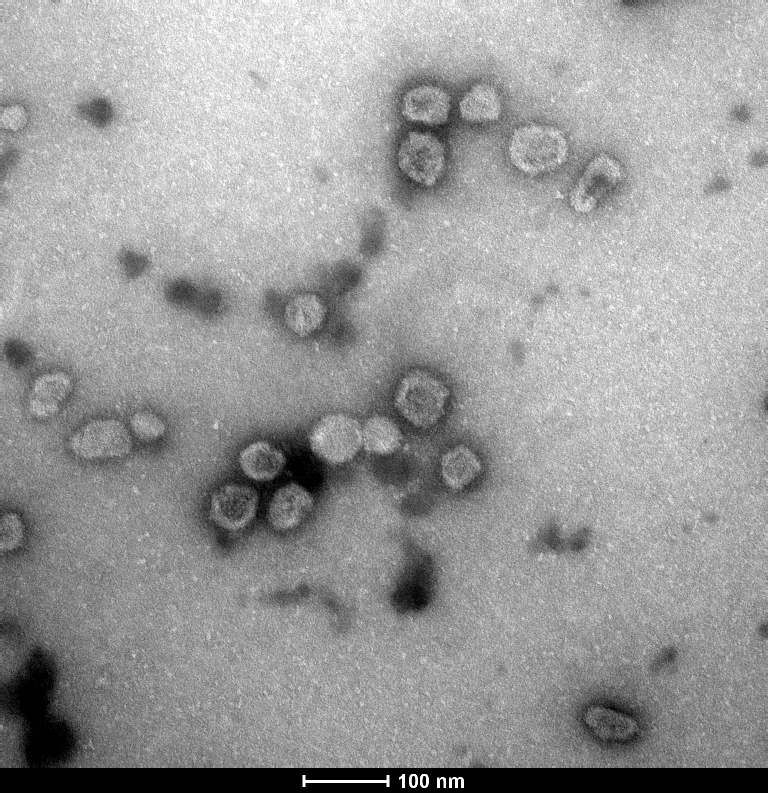

Supplement: Supplementary Figure 1 — The level of ELFN1-AS1 is upregulated in SARC tissues. [file DataSheet_1.zip › Figure 4/A/143BsiRNA NC-Exo.tif]

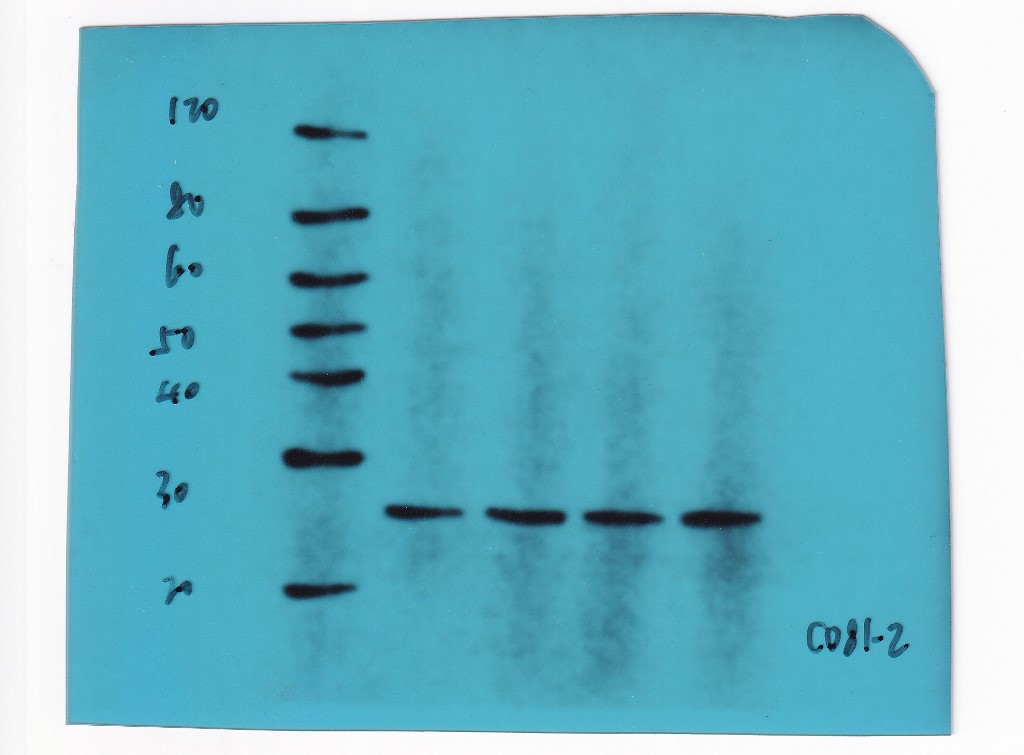

Supplement: Supplementary Figure 1 — The level of ELFN1-AS1 is upregulated in SARC tissues. [file DataSheet_1.zip › Figure 4/B/Figure 4B-CD81.jpg]

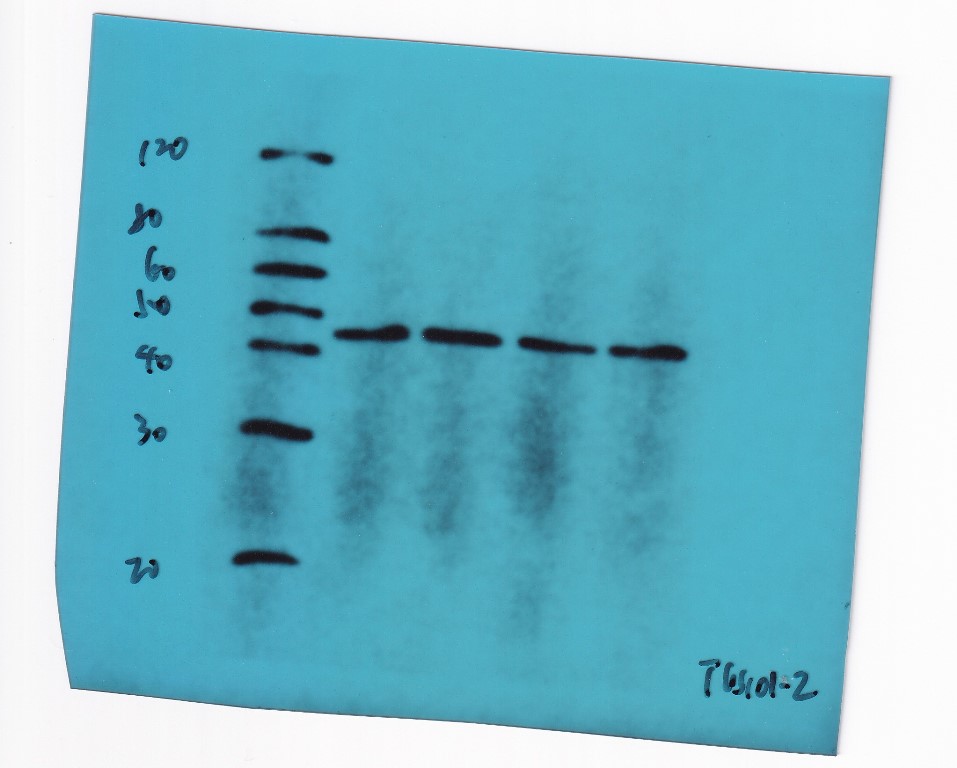

Supplement: Supplementary Figure 1 — The level of ELFN1-AS1 is upregulated in SARC tissues. [file DataSheet_1.zip › Figure 4/B/Figure 4B-TSG101.jpg]

# Overall Survival

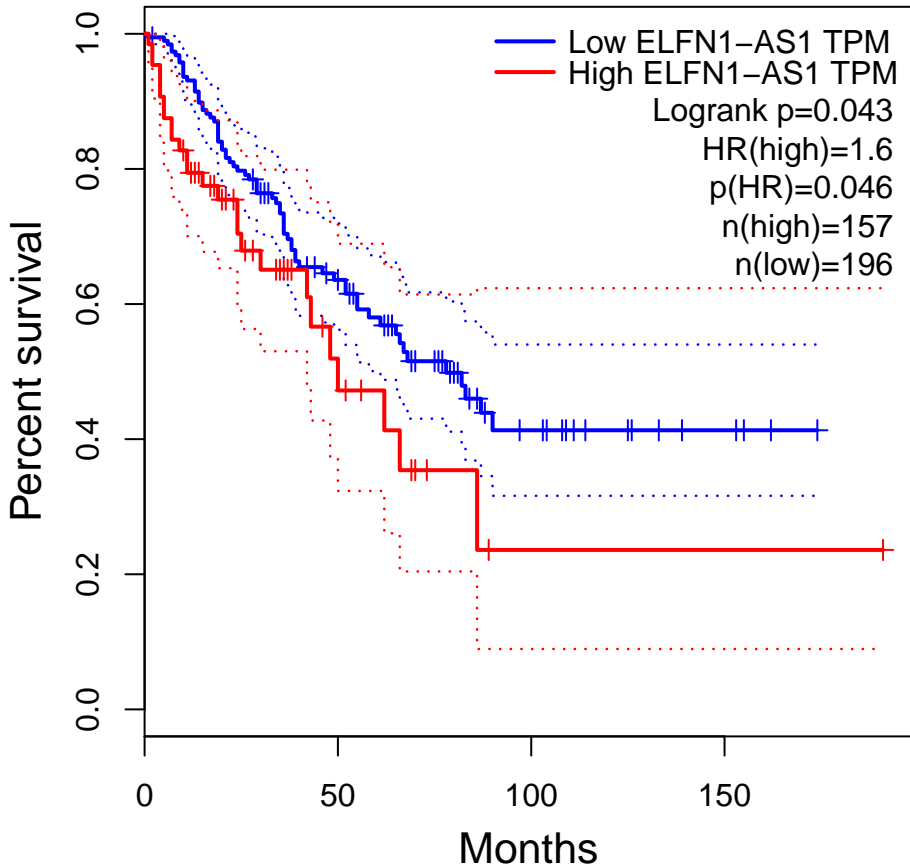

Supplement: Supplementary Figure 1 — The level of ELFN1-AS1 is upregulated in SARC tissues. [file DataSheet_1.zip › Figure 1/B.pdf]

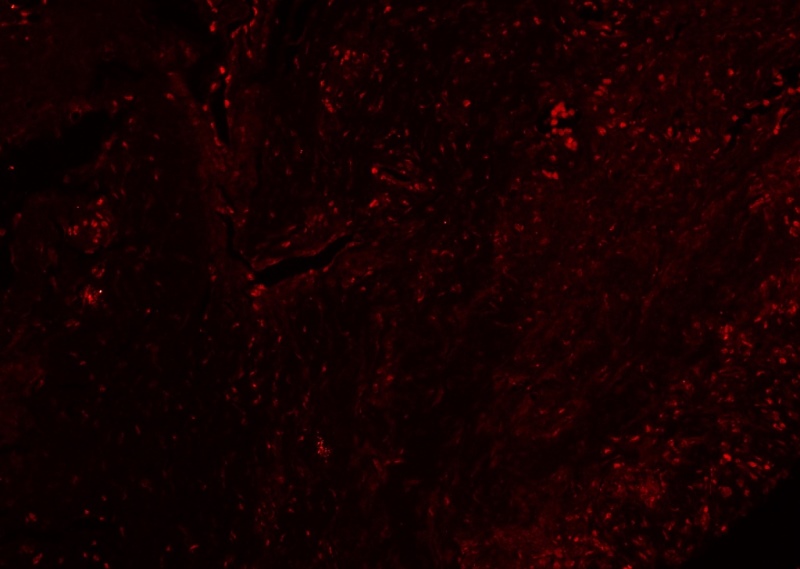

Supplement: Supplementary Figure 1 — The level of ELFN1-AS1 is upregulated in SARC tissues. [file DataSheet_1.zip › Figure 1/E/Normal 1.jpg]

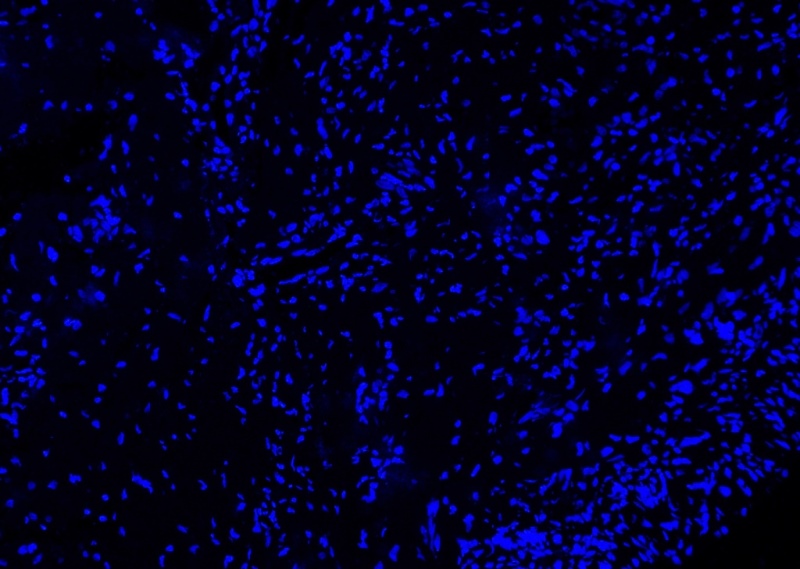

Supplement: Supplementary Figure 1 — The level of ELFN1-AS1 is upregulated in SARC tissues. [file DataSheet_1.zip › Figure 1/E/Normal 2.jpg]

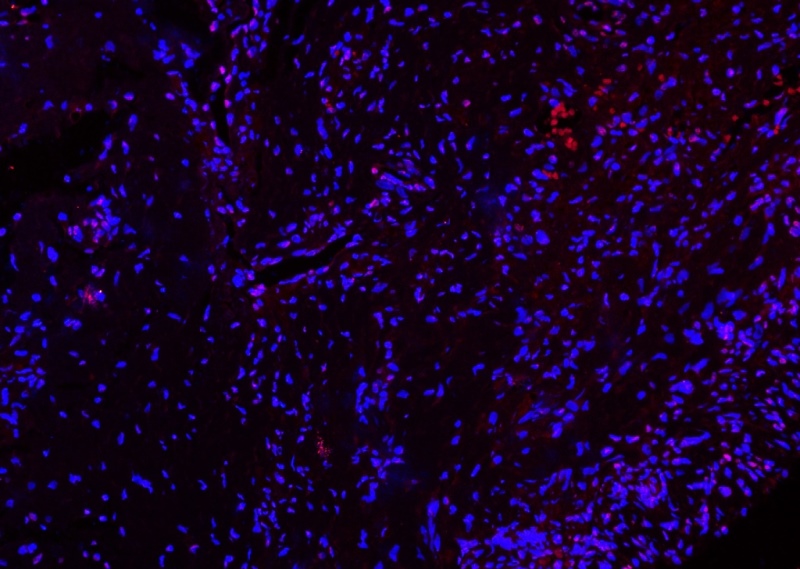

Supplement: Supplementary Figure 1 — The level of ELFN1-AS1 is upregulated in SARC tissues. [file DataSheet_1.zip › Figure 1/E/Normal merge.jpg]

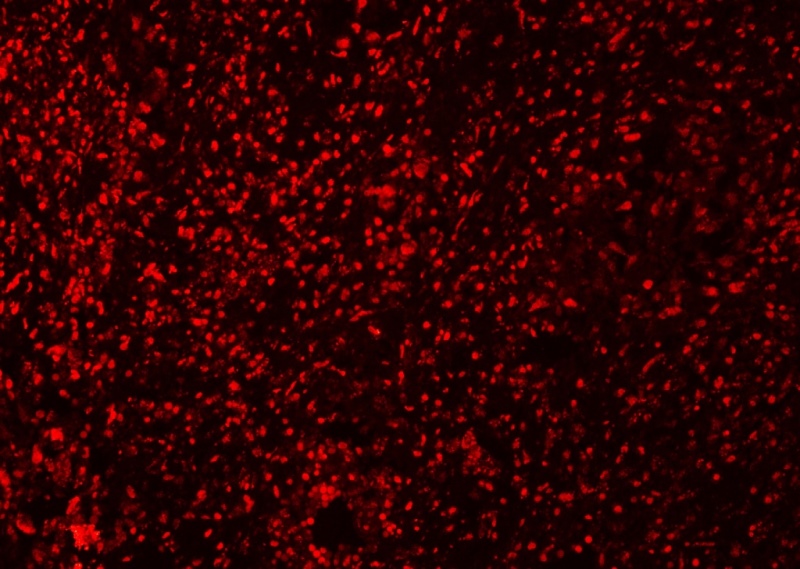

Supplement: Supplementary Figure 1 — The level of ELFN1-AS1 is upregulated in SARC tissues. [file DataSheet_1.zip › Figure 1/E/Tumor 1.jpg]

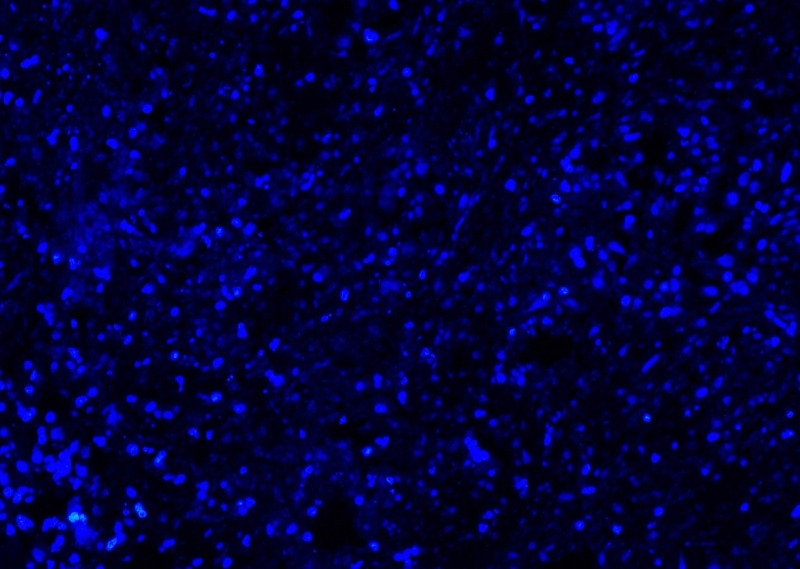

Supplement: Supplementary Figure 1 — The level of ELFN1-AS1 is upregulated in SARC tissues. [file DataSheet_1.zip › Figure 1/E/Tumor 2.jpg]

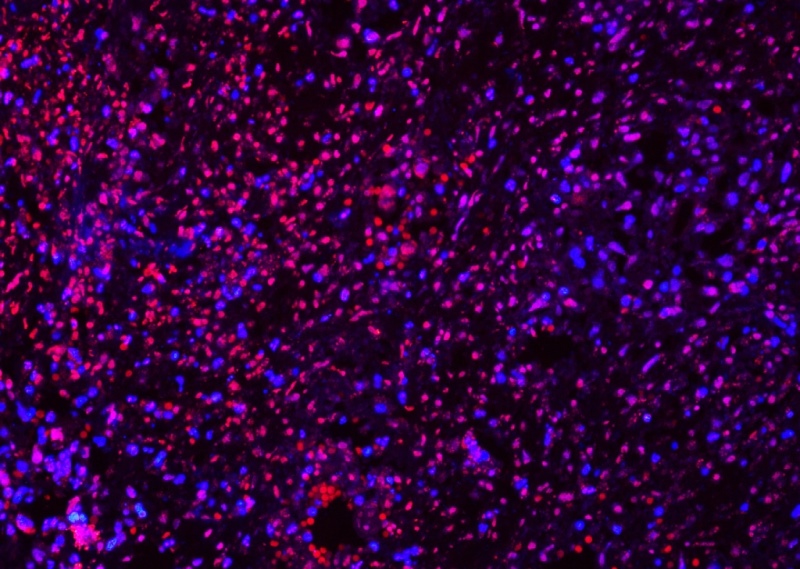

Supplement: Supplementary Figure 1 — The level of ELFN1-AS1 is upregulated in SARC tissues. [file DataSheet_1.zip › Figure 1/E/Tumor merge.jpg]

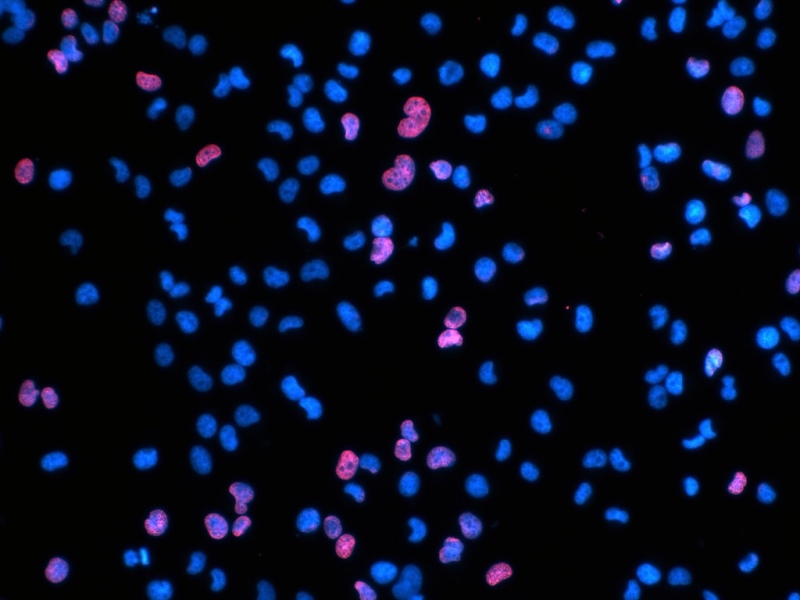

Supplement: Supplementary Figure 1 — The level of ELFN1-AS1 is upregulated in SARC tissues. [file DataSheet_1.zip › Figure 2/E/143B/143B ELFN1-AS1-siRNA 200-3+4.jpg]

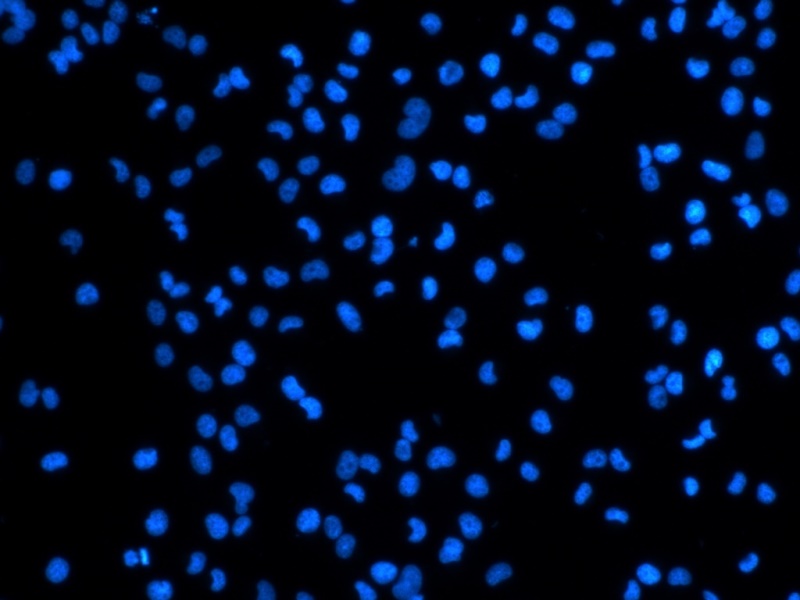

Supplement: Supplementary Figure 1 — The level of ELFN1-AS1 is upregulated in SARC tissues. [file DataSheet_1.zip › Figure 2/E/143B/143B ELFN1-AS1-siRNA 200-3.jpg]

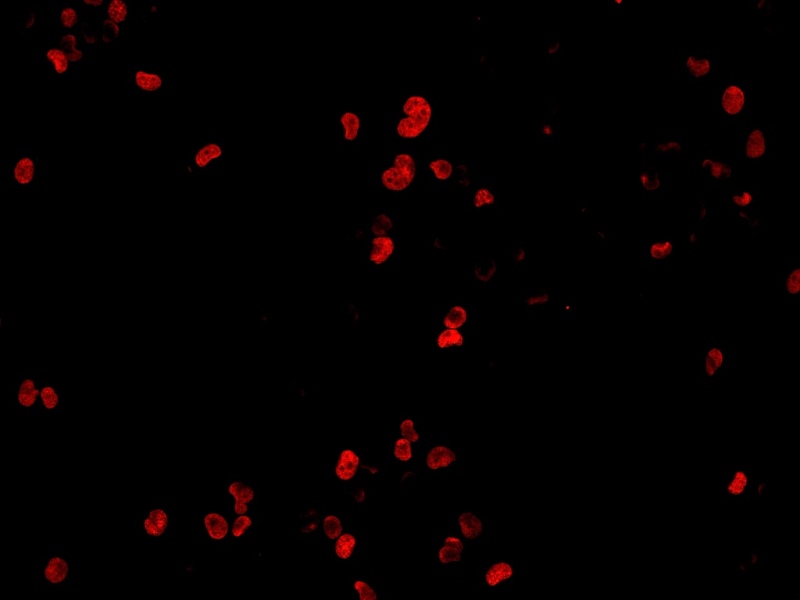

Supplement: Supplementary Figure 1 — The level of ELFN1-AS1 is upregulated in SARC tissues. [file DataSheet_1.zip › Figure 2/E/143B/143B ELFN1-AS1-siRNA 200-4.jpg]

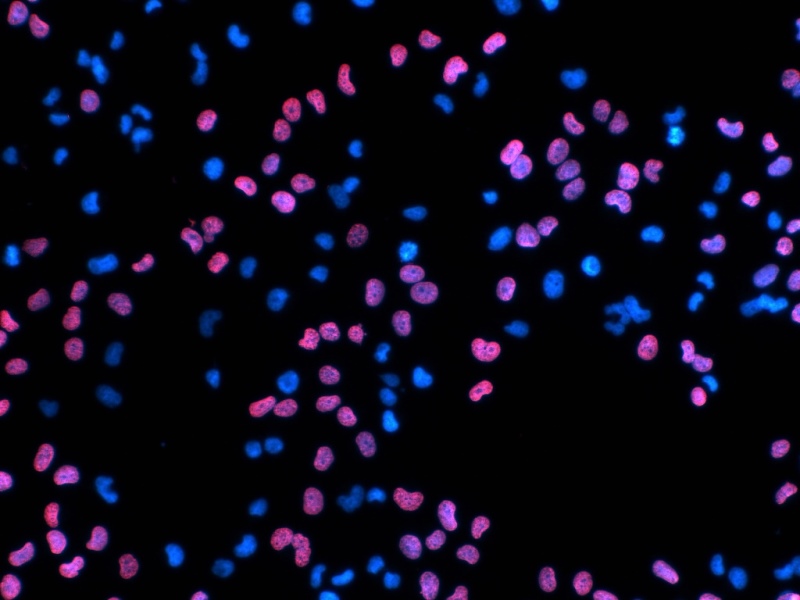

Supplement: Supplementary Figure 1 — The level of ELFN1-AS1 is upregulated in SARC tissues. [file DataSheet_1.zip › Figure 2/E/143B/143B ELFN1-AS1-siRNA-NC 200-1+2.jpg]

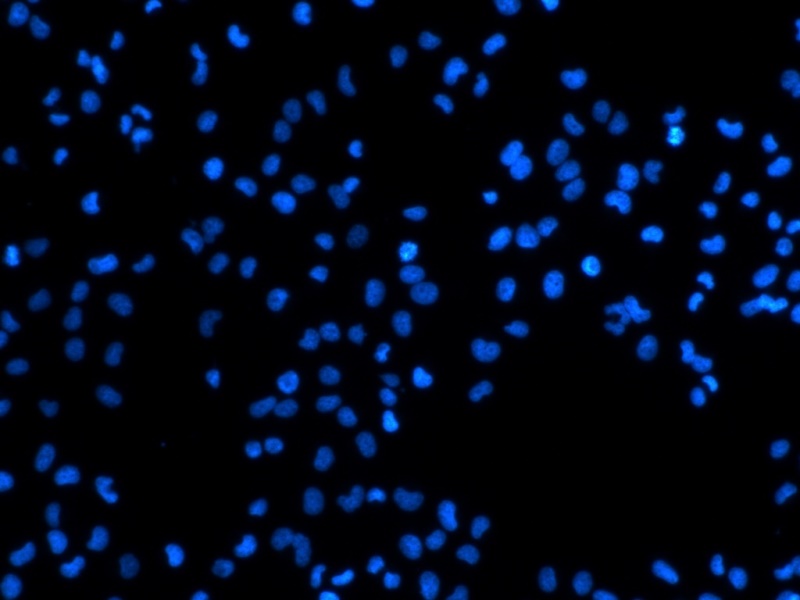

Supplement: Supplementary Figure 1 — The level of ELFN1-AS1 is upregulated in SARC tissues. [file DataSheet_1.zip › Figure 2/E/143B/143B ELFN1-AS1-siRNA-NC 200-1.jpg]

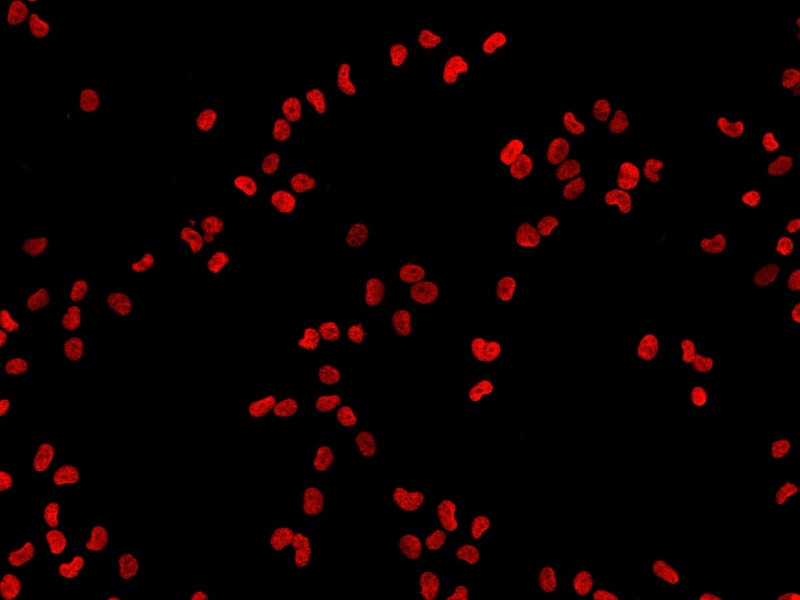

Supplement: Supplementary Figure 1 — The level of ELFN1-AS1 is upregulated in SARC tissues. [file DataSheet_1.zip › Figure 2/E/143B/143B ELFN1-AS1-siRNA-NC 200-2.jpg]

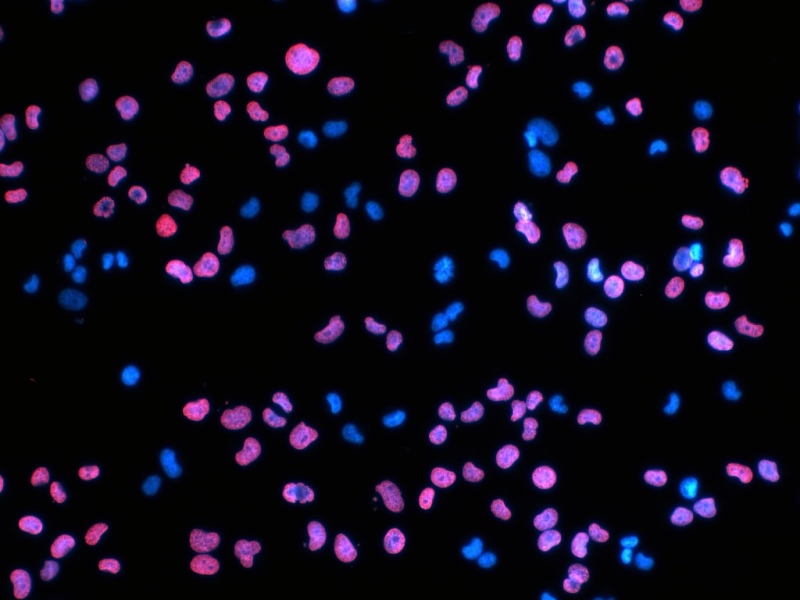

Supplement: Supplementary Figure 1 — The level of ELFN1-AS1 is upregulated in SARC tissues. [file DataSheet_1.zip › Figure 2/E/143B/143B pcDNA3.1 200-1+2.jpg]

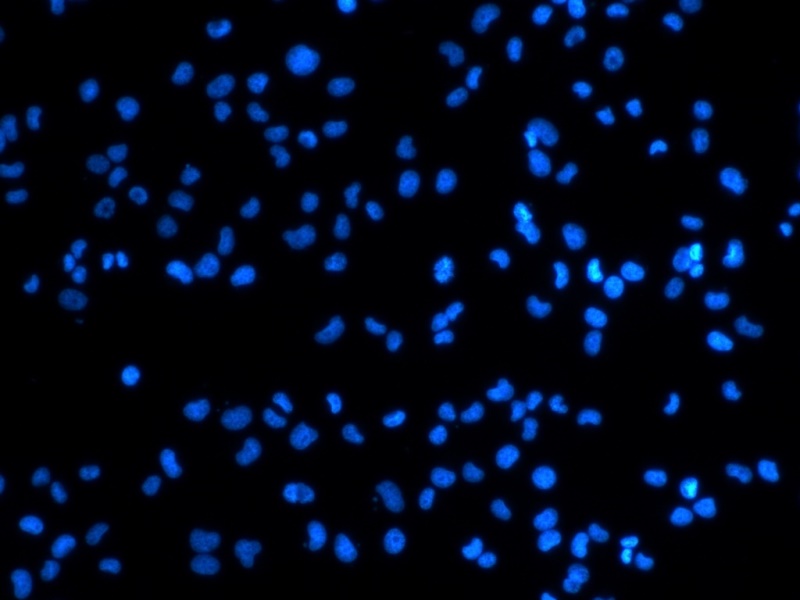

Supplement: Supplementary Figure 1 — The level of ELFN1-AS1 is upregulated in SARC tissues. [file DataSheet_1.zip › Figure 2/E/143B/143B pcDNA3.1 200-1.jpg]

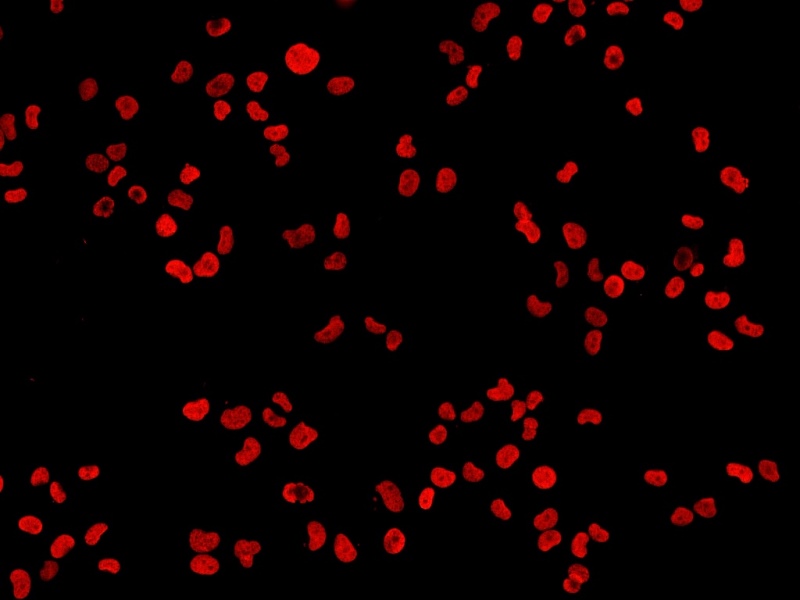

Supplement: Supplementary Figure 1 — The level of ELFN1-AS1 is upregulated in SARC tissues. [file DataSheet_1.zip › Figure 2/E/143B/143B pcDNA3.1 200-2.jpg]

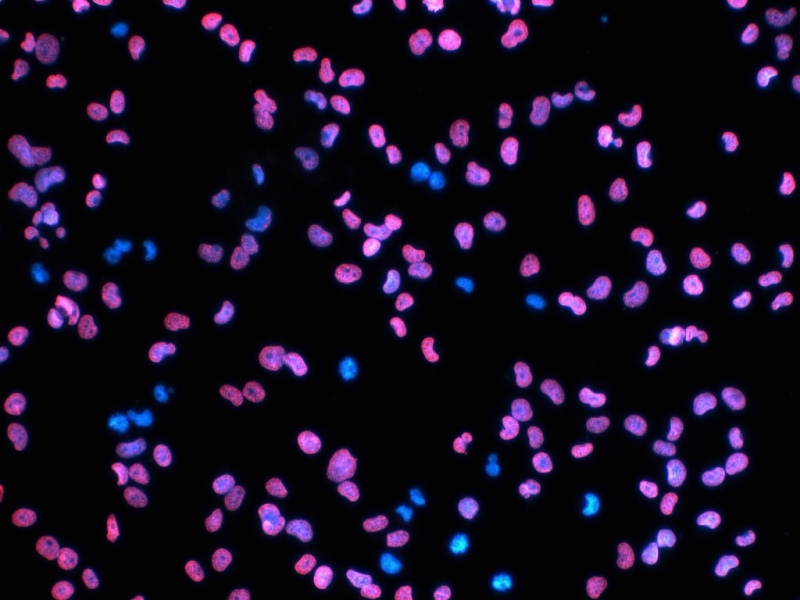

Supplement: Supplementary Figure 1 — The level of ELFN1-AS1 is upregulated in SARC tissues. [file DataSheet_1.zip › Figure 2/E/143B/143B pcDNA3.1-ELFN1-AS1 200-5+6.jpg]

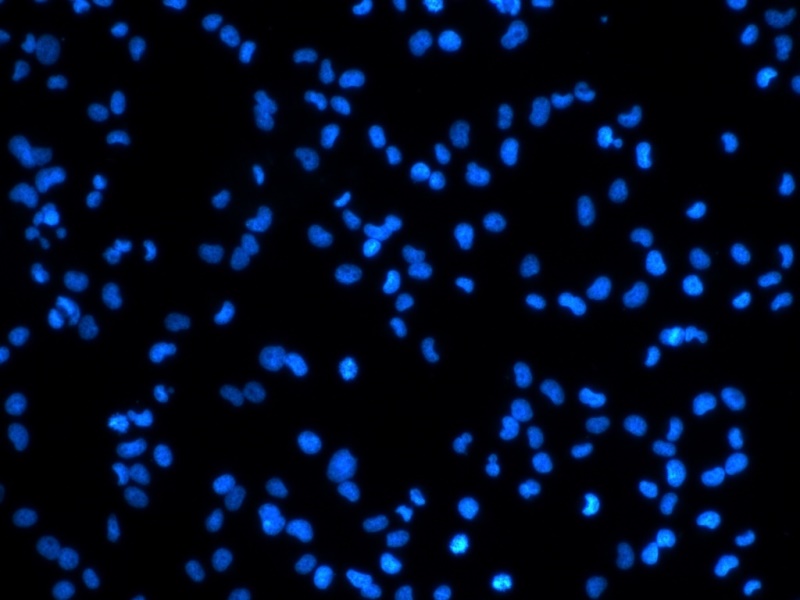

Supplement: Supplementary Figure 1 — The level of ELFN1-AS1 is upregulated in SARC tissues. [file DataSheet_1.zip › Figure 2/E/143B/143B pcDNA3.1-ELFN1-AS1 200-5.jpg]

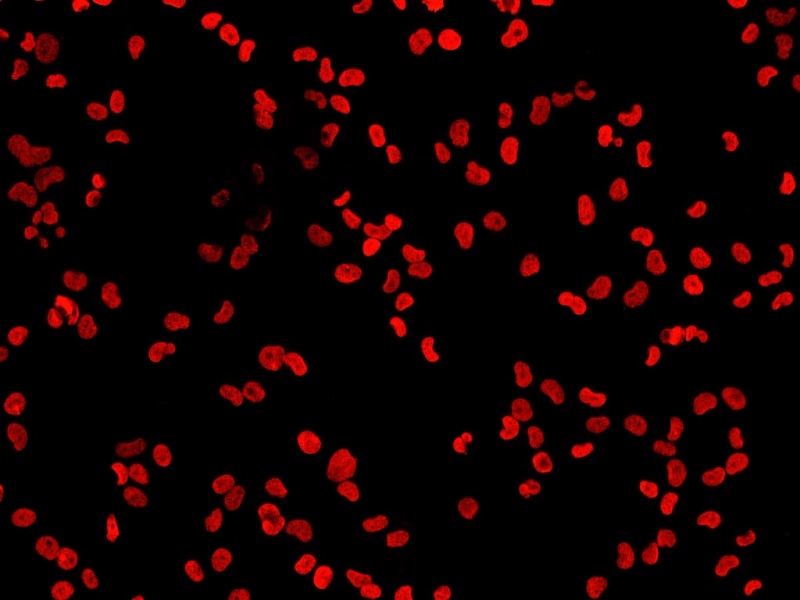

Supplement: Supplementary Figure 1 — The level of ELFN1-AS1 is upregulated in SARC tissues. [file DataSheet_1.zip › Figure 2/E/143B/143B pcDNA3.1-ELFN1-AS1 200-6.jpg]

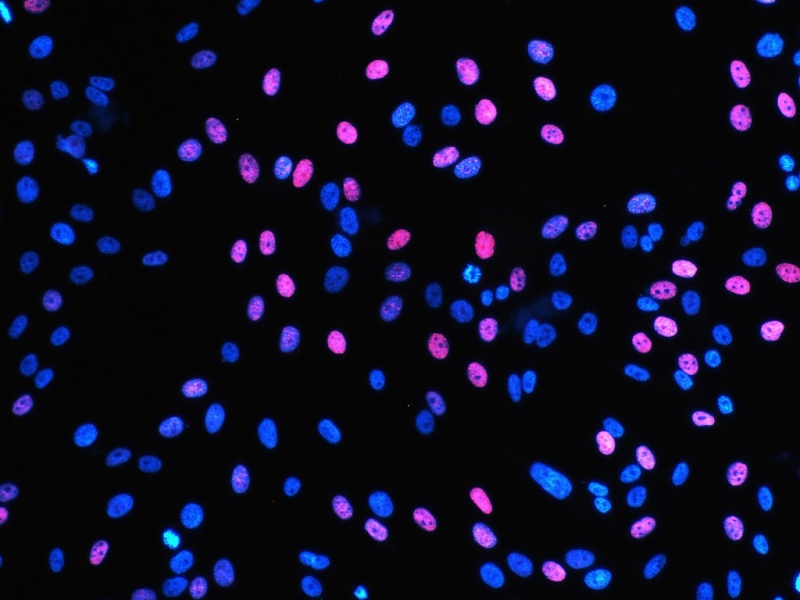

Supplement: Supplementary Figure 1 — The level of ELFN1-AS1 is upregulated in SARC tissues. [file DataSheet_1.zip › Figure 2/E/MG63/MG-63 ELFN1-AS1-siRNA-NC 200-1+2.JPG]

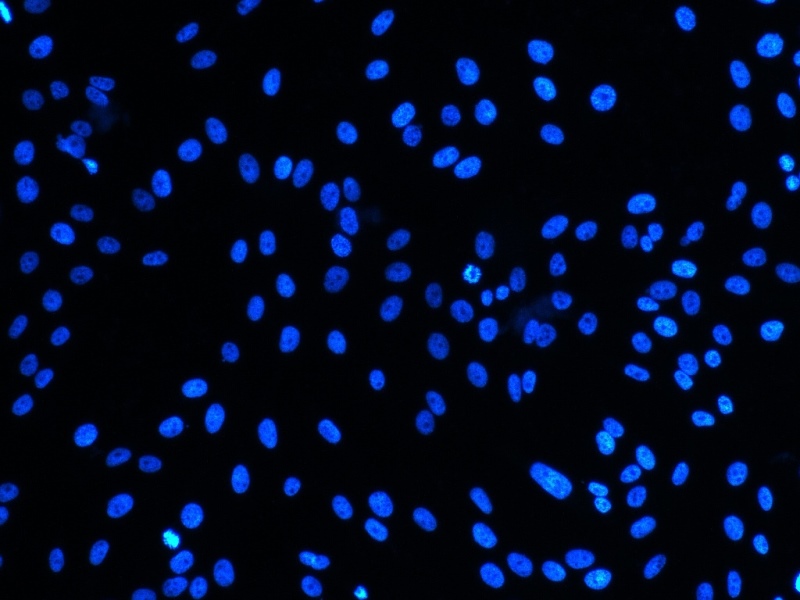

Supplement: Supplementary Figure 1 — The level of ELFN1-AS1 is upregulated in SARC tissues. [file DataSheet_1.zip › Figure 2/E/MG63/MG-63 ELFN1-AS1-siRNA-NC 200-1.jpg]

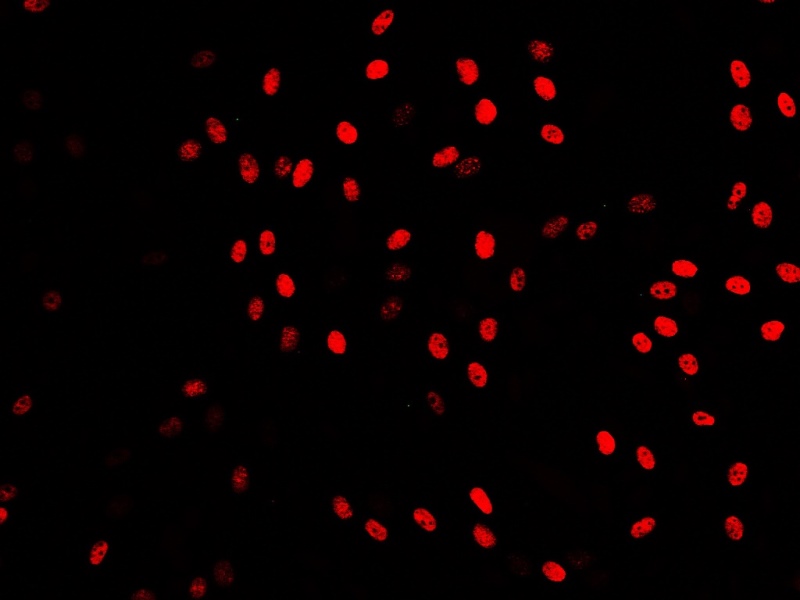

Supplement: Supplementary Figure 1 — The level of ELFN1-AS1 is upregulated in SARC tissues. [file DataSheet_1.zip › Figure 2/E/MG63/MG-63 ELFN1-AS1-siRNA-NC 200-2.jpg]

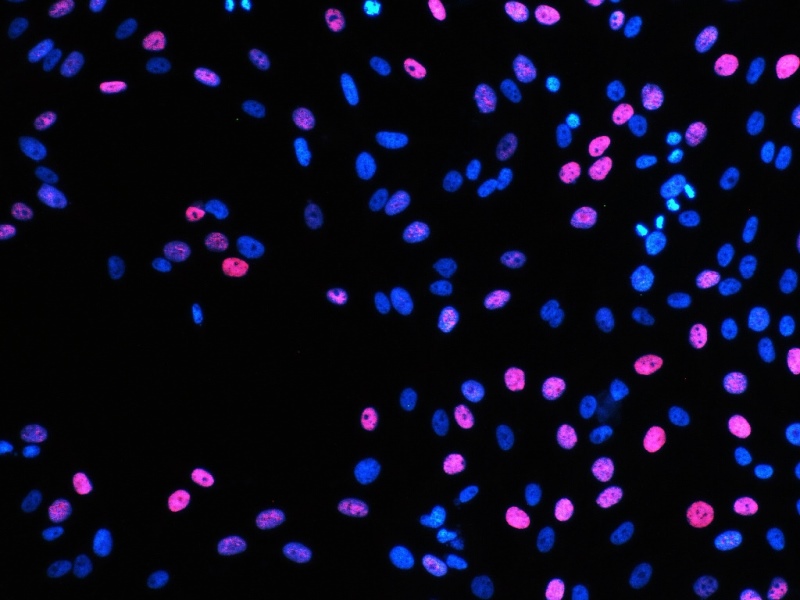

Supplement: Supplementary Figure 1 — The level of ELFN1-AS1 is upregulated in SARC tissues. [file DataSheet_1.zip › Figure 2/E/MG63/MG-63 ELFN1-AS1-siRNA1 200-1+2.JPG]

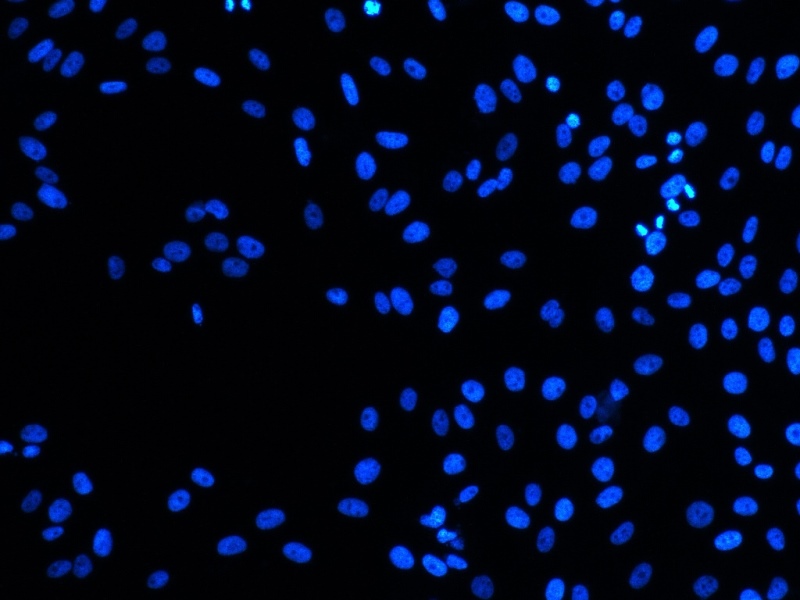

Supplement: Supplementary Figure 1 — The level of ELFN1-AS1 is upregulated in SARC tissues. [file DataSheet_1.zip › Figure 2/E/MG63/MG-63 ELFN1-AS1-siRNA1 200-1.jpg]

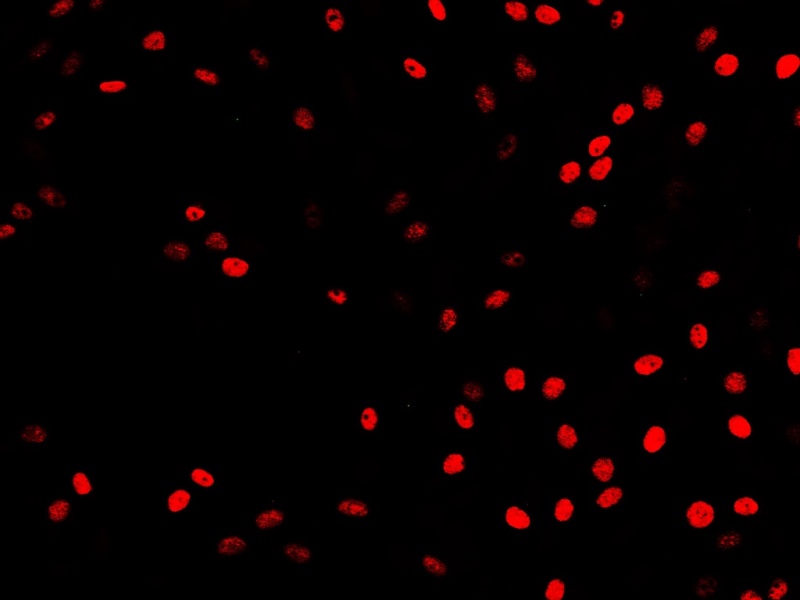

Supplement: Supplementary Figure 1 — The level of ELFN1-AS1 is upregulated in SARC tissues. [file DataSheet_1.zip › Figure 2/E/MG63/MG-63 ELFN1-AS1-siRNA1 200-2.jpg]

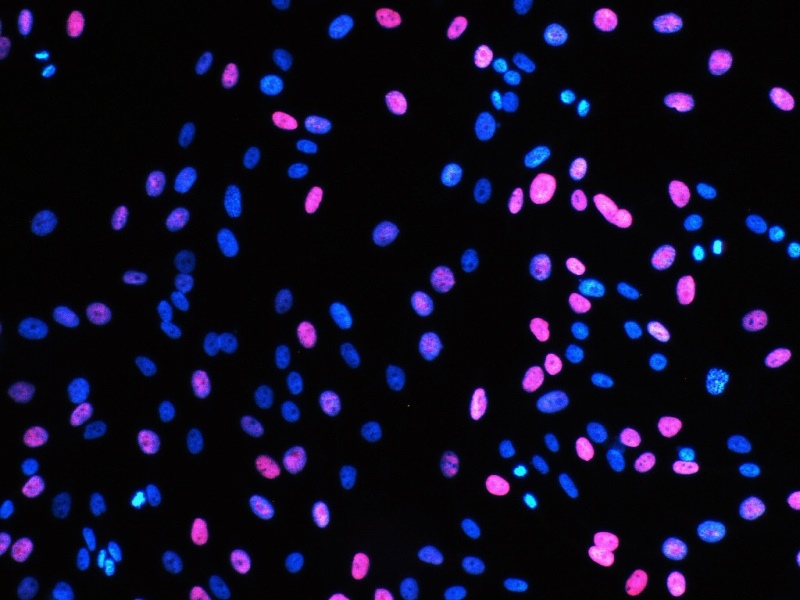

Supplement: Supplementary Figure 1 — The level of ELFN1-AS1 is upregulated in SARC tissues. [file DataSheet_1.zip › Figure 2/E/MG63/MG-63 pcDNA3.1 200-1+2.jpg]

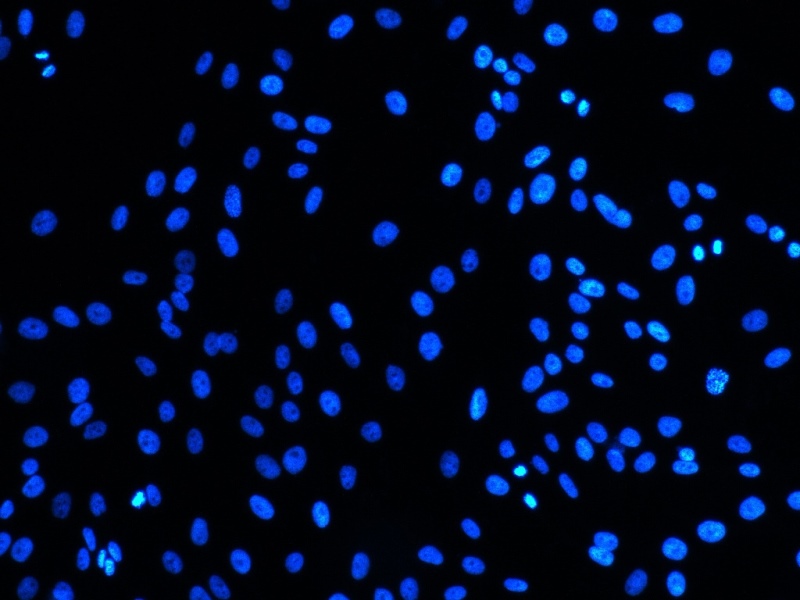

Supplement: Supplementary Figure 1 — The level of ELFN1-AS1 is upregulated in SARC tissues. [file DataSheet_1.zip › Figure 2/E/MG63/MG-63 pcDNA3.1 200-1.jpg]

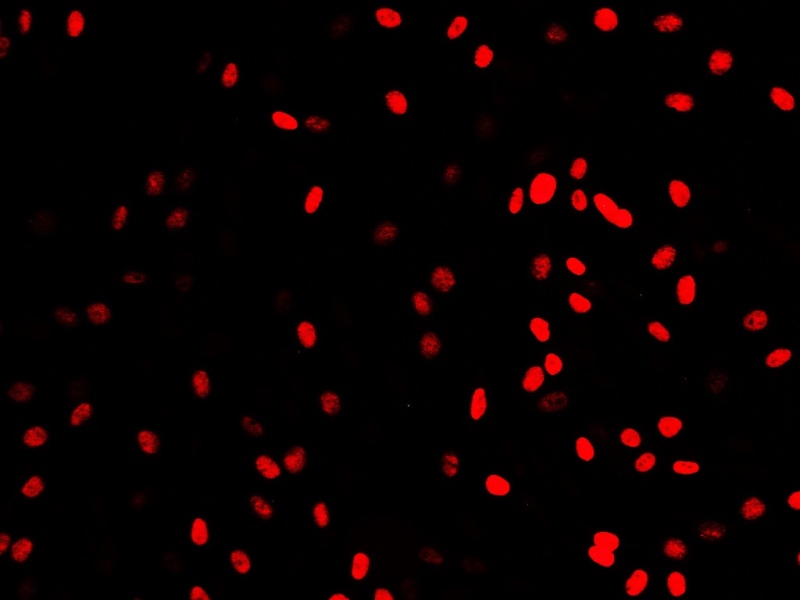

Supplement: Supplementary Figure 1 — The level of ELFN1-AS1 is upregulated in SARC tissues. [file DataSheet_1.zip › Figure 2/E/MG63/MG-63 pcDNA3.1 200-2.jpg]

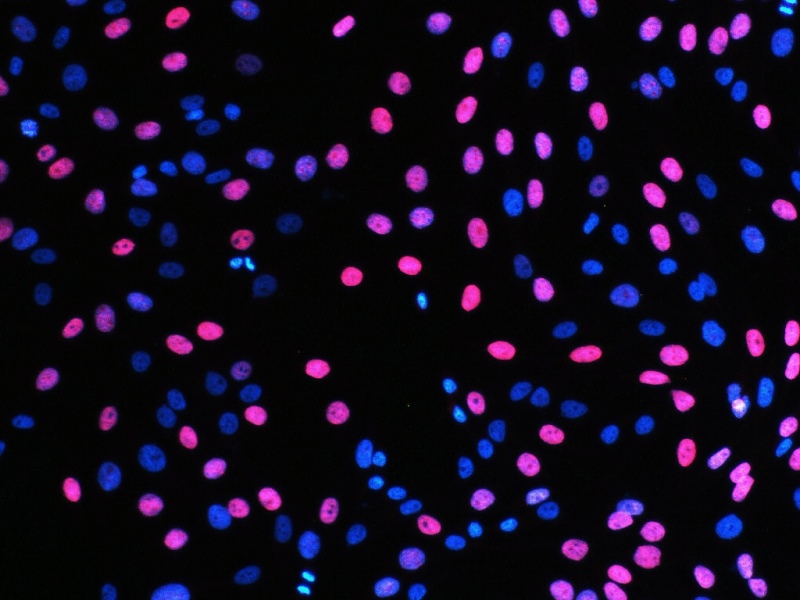

Supplement: Supplementary Figure 1 — The level of ELFN1-AS1 is upregulated in SARC tissues. [file DataSheet_1.zip › Figure 2/E/MG63/MG-63 pcDNA3.1-ELFN1/250CAS1 200-7+8.jpg]

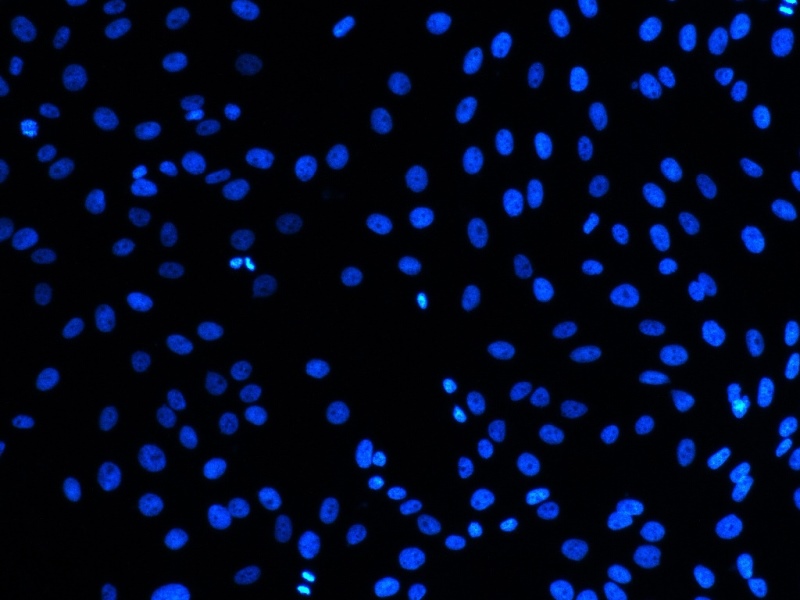

Supplement: Supplementary Figure 1 — The level of ELFN1-AS1 is upregulated in SARC tissues. [file DataSheet_1.zip › Figure 2/E/MG63/MG-63 pcDNA3.1-ELFN1/250CAS1 200-7.jpg]

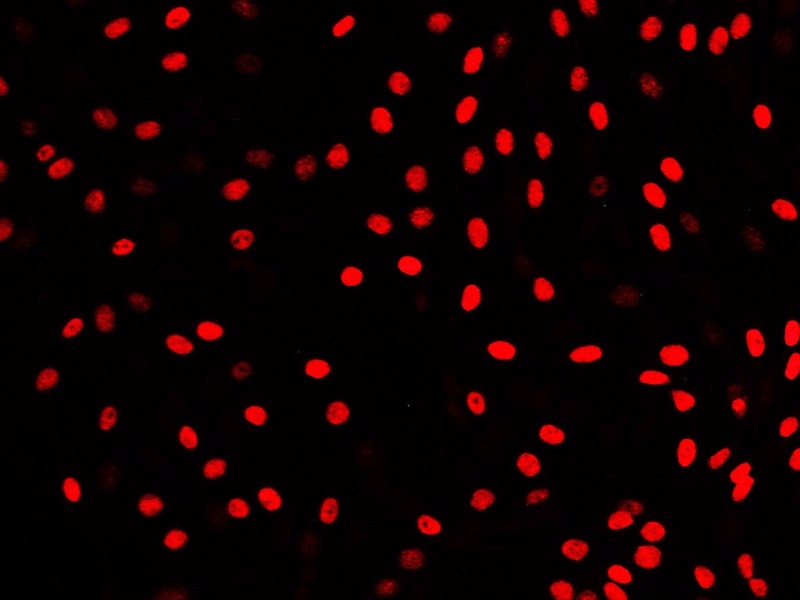

Supplement: Supplementary Figure 1 — The level of ELFN1-AS1 is upregulated in SARC tissues. [file DataSheet_1.zip › Figure 2/E/MG63/MG-63 pcDNA3.1-ELFN1/250CAS1 200-8.jpg]

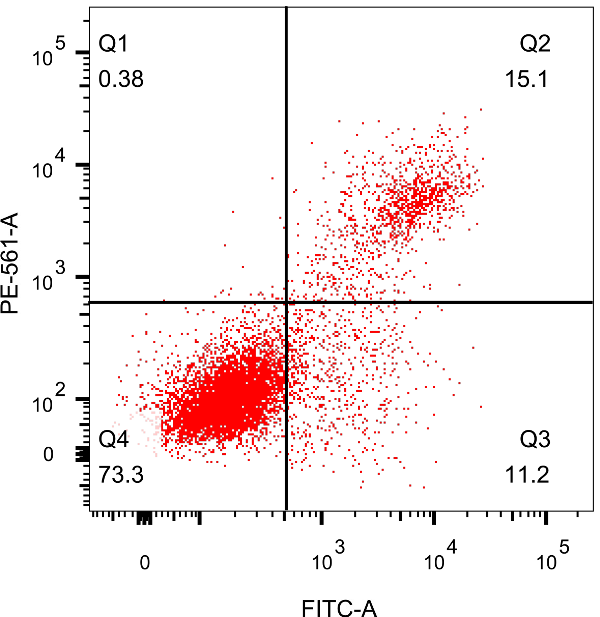

Supplement: Supplementary Figure 1 — The level of ELFN1-AS1 is upregulated in SARC tissues. [file DataSheet_1.zip › Figure 2/F/143B/ELFN1-AS1-siRNA1/1.tif]

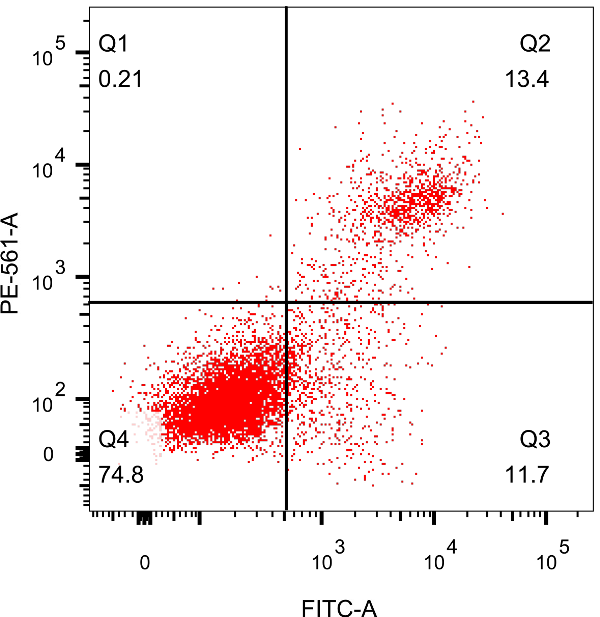

Supplement: Supplementary Figure 1 — The level of ELFN1-AS1 is upregulated in SARC tissues. [file DataSheet_1.zip › Figure 2/F/143B/ELFN1-AS1-siRNA1/2.tif]

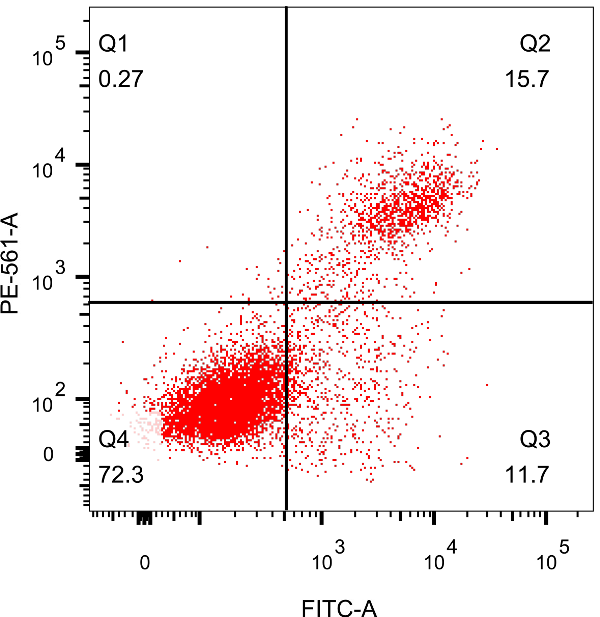

Supplement: Supplementary Figure 1 — The level of ELFN1-AS1 is upregulated in SARC tissues. [file DataSheet_1.zip › Figure 2/F/143B/ELFN1-AS1-siRNA1/3.tif]

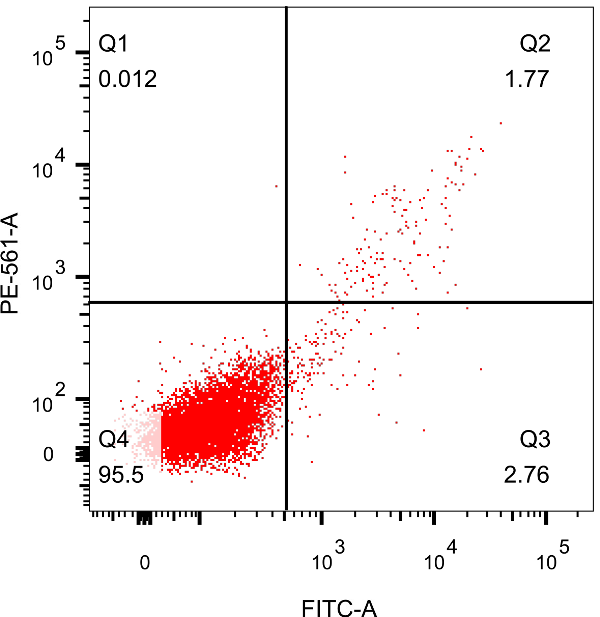

Supplement: Supplementary Figure 1 — The level of ELFN1-AS1 is upregulated in SARC tissues. [file DataSheet_1.zip › Figure 2/F/143B/pcDNA3.1/1.tif]

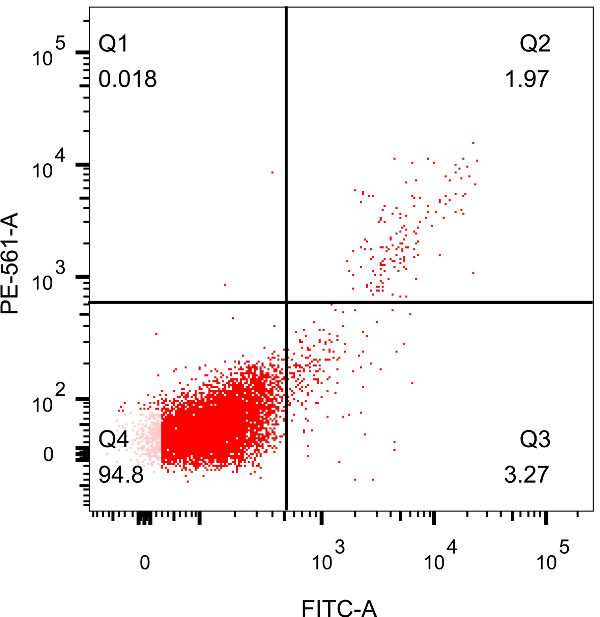

Supplement: Supplementary Figure 1 — The level of ELFN1-AS1 is upregulated in SARC tissues. [file DataSheet_1.zip › Figure 2/F/143B/pcDNA3.1/2.tif]

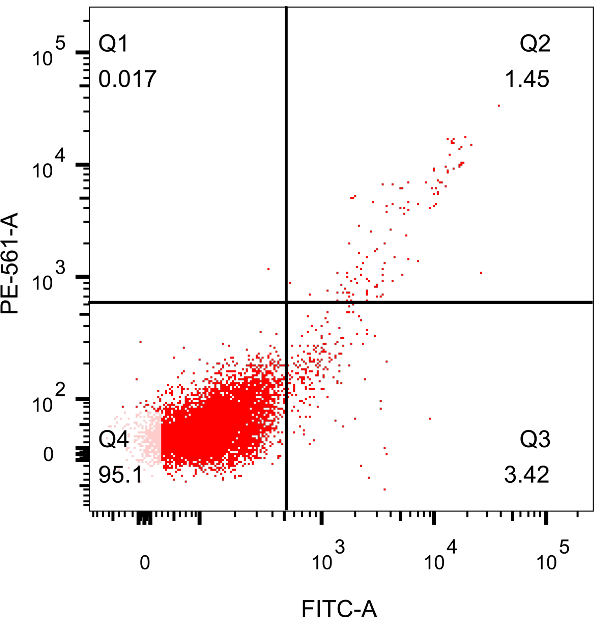

Supplement: Supplementary Figure 1 — The level of ELFN1-AS1 is upregulated in SARC tissues. [file DataSheet_1.zip › Figure 2/F/143B/pcDNA3.1/3.tif]

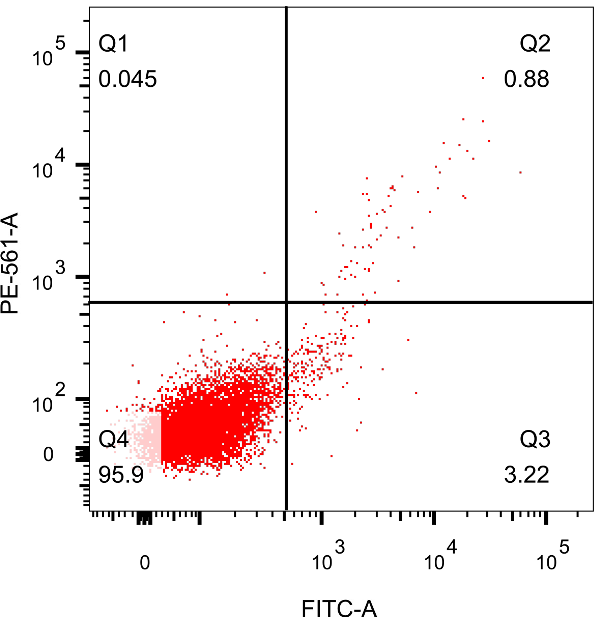

Supplement: Supplementary Figure 1 — The level of ELFN1-AS1 is upregulated in SARC tissues. [file DataSheet_1.zip › Figure 2/F/143B/pcDNA3.1-ELFN1-AS1/1.tif]

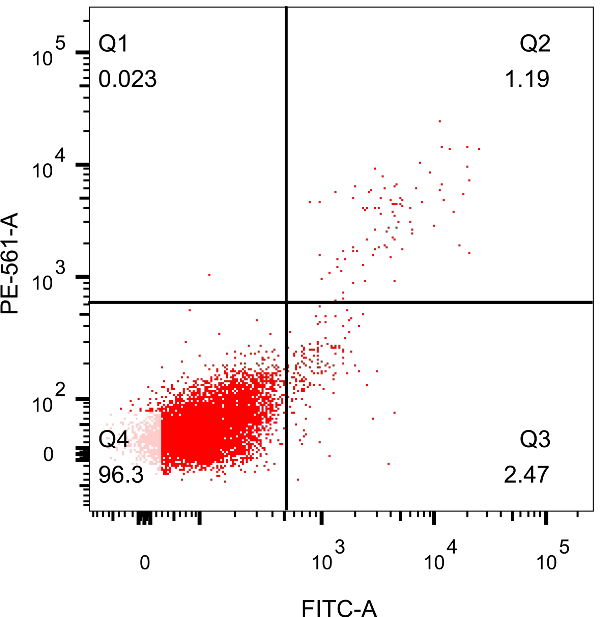

Supplement: Supplementary Figure 1 — The level of ELFN1-AS1 is upregulated in SARC tissues. [file DataSheet_1.zip › Figure 2/F/143B/pcDNA3.1-ELFN1-AS1/2.tif]

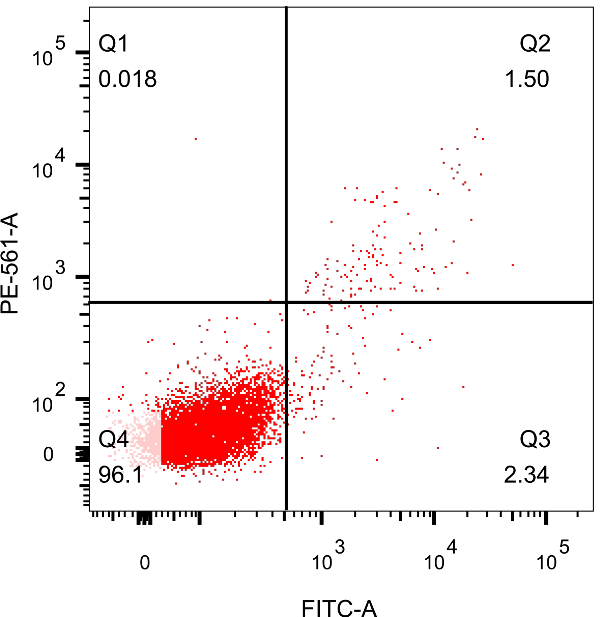

Supplement: Supplementary Figure 1 — The level of ELFN1-AS1 is upregulated in SARC tissues. [file DataSheet_1.zip › Figure 2/F/143B/pcDNA3.1-ELFN1-AS1/3.tif]

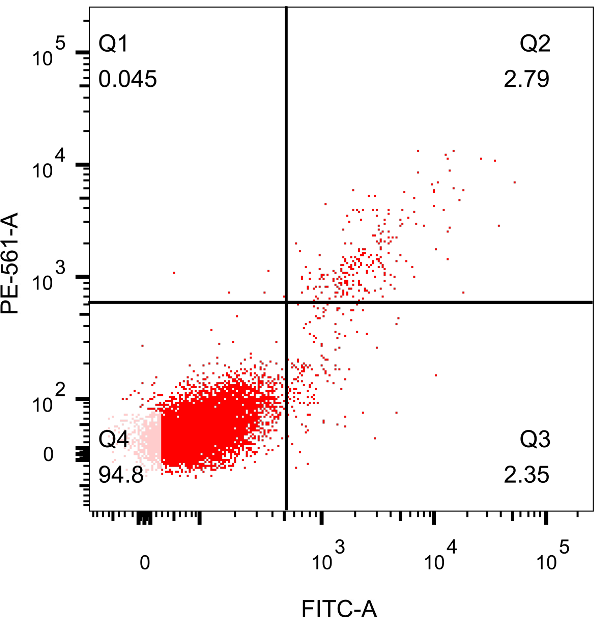

Supplement: Supplementary Figure 1 — The level of ELFN1-AS1 is upregulated in SARC tissues. [file DataSheet_1.zip › Figure 2/F/143B/si-NC/1.tif]

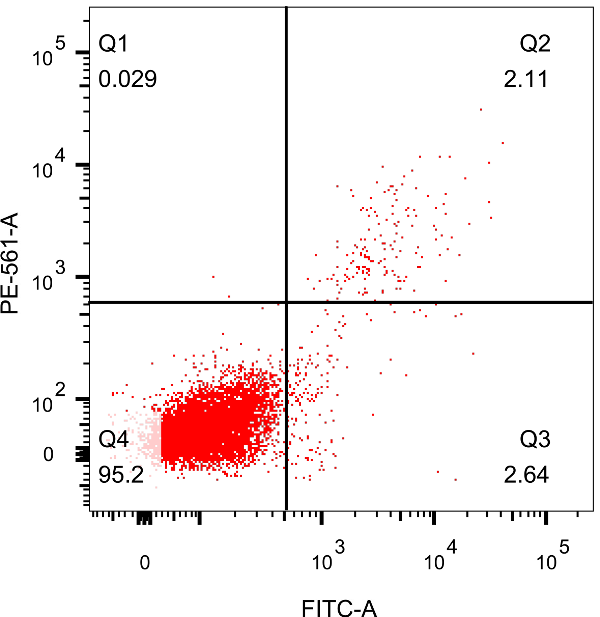

Supplement: Supplementary Figure 1 — The level of ELFN1-AS1 is upregulated in SARC tissues. [file DataSheet_1.zip › Figure 2/F/143B/si-NC/2.tif]

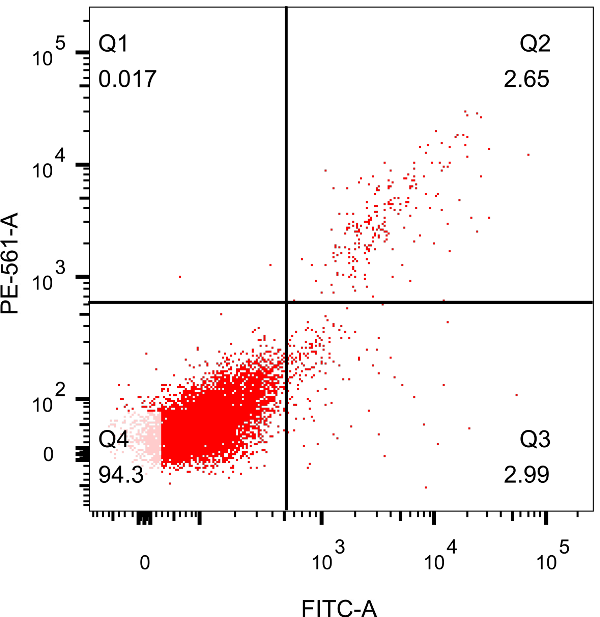

Supplement: Supplementary Figure 1 — The level of ELFN1-AS1 is upregulated in SARC tissues. [file DataSheet_1.zip › Figure 2/F/143B/si-NC/3.tif]

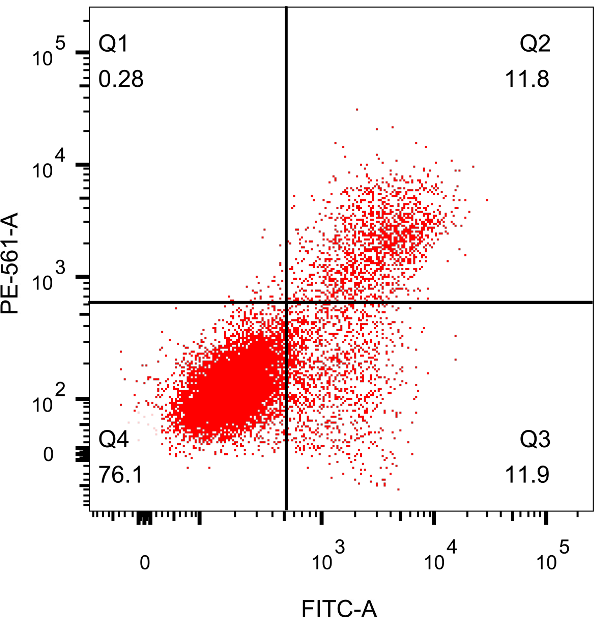

Supplement: Supplementary Figure 1 — The level of ELFN1-AS1 is upregulated in SARC tissues. [file DataSheet_1.zip › Figure 2/F/MG63/ELFN1-AS1-siRNA1/1.tif]

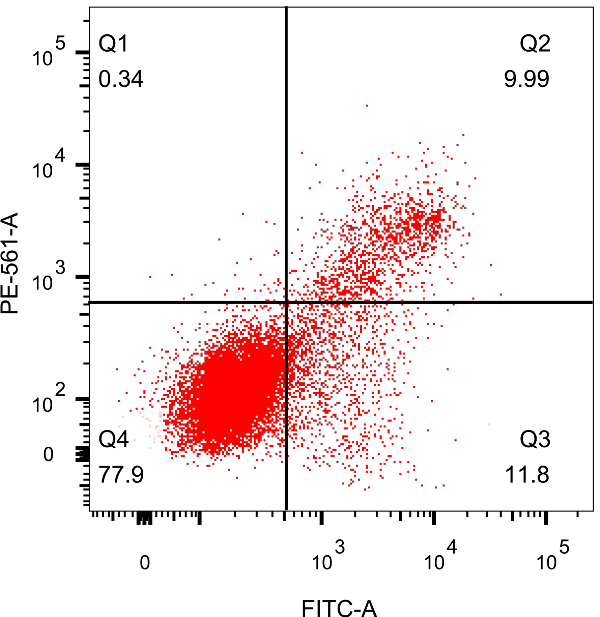

Supplement: Supplementary Figure 1 — The level of ELFN1-AS1 is upregulated in SARC tissues. [file DataSheet_1.zip › Figure 2/F/MG63/ELFN1-AS1-siRNA1/2.tif]

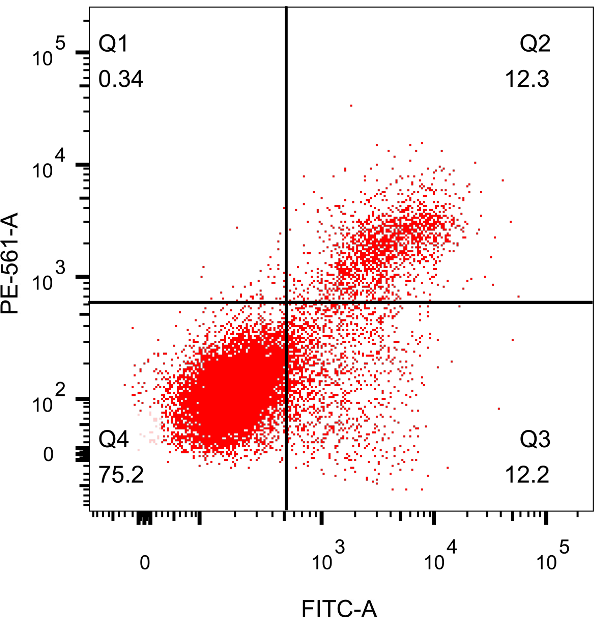

Supplement: Supplementary Figure 1 — The level of ELFN1-AS1 is upregulated in SARC tissues. [file DataSheet_1.zip › Figure 2/F/MG63/ELFN1-AS1-siRNA1/3.tif]

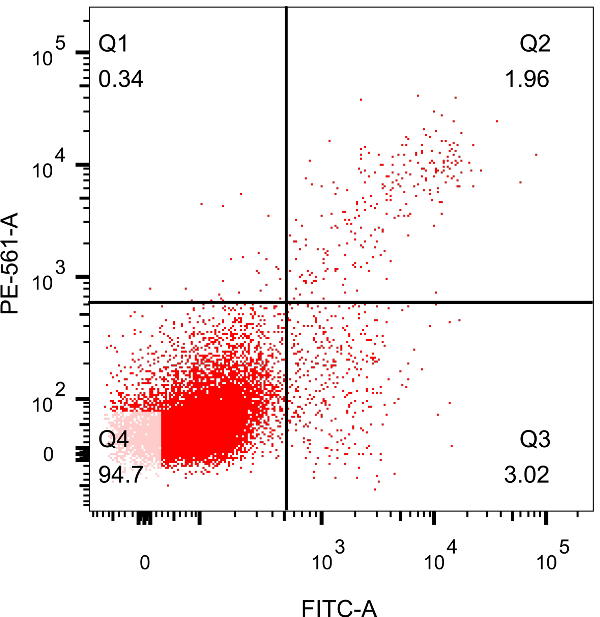

Supplement: Supplementary Figure 1 — The level of ELFN1-AS1 is upregulated in SARC tissues. [file DataSheet_1.zip › Figure 2/F/MG63/pcDNA3.1/1.tif]

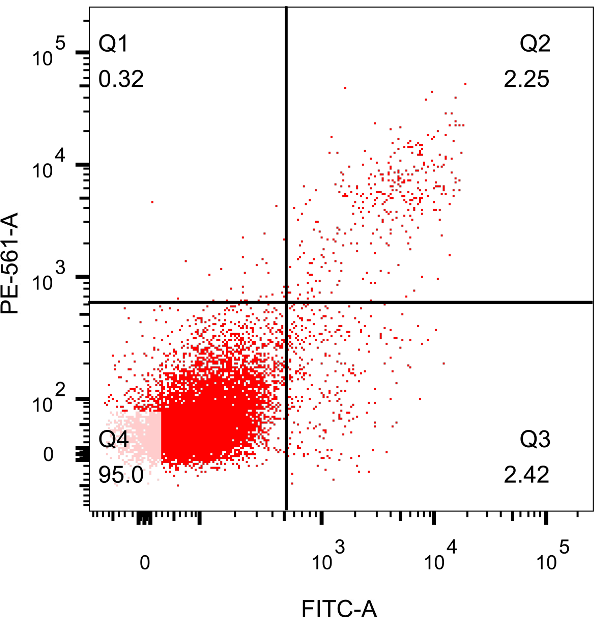

Supplement: Supplementary Figure 1 — The level of ELFN1-AS1 is upregulated in SARC tissues. [file DataSheet_1.zip › Figure 2/F/MG63/pcDNA3.1/2.tif]

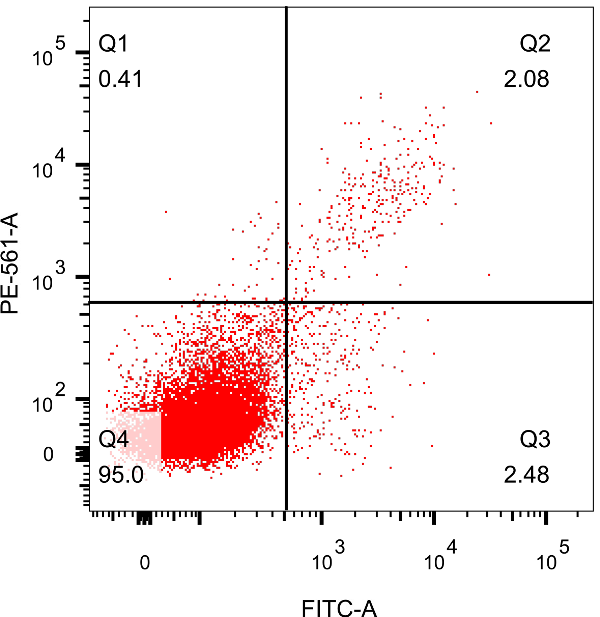

Supplement: Supplementary Figure 1 — The level of ELFN1-AS1 is upregulated in SARC tissues. [file DataSheet_1.zip › Figure 2/F/MG63/pcDNA3.1/3.tif]

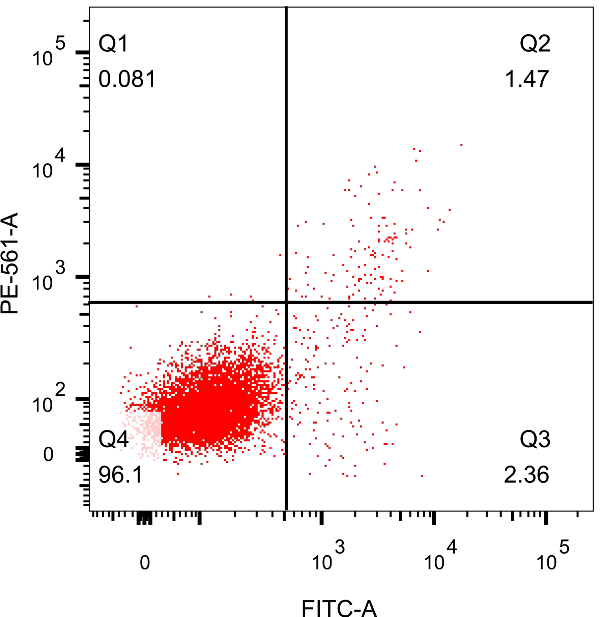

Supplement: Supplementary Figure 1 — The level of ELFN1-AS1 is upregulated in SARC tissues. [file DataSheet_1.zip › Figure 2/F/MG63/pcDNA3.1-ELFN1-AS1/1.tif]

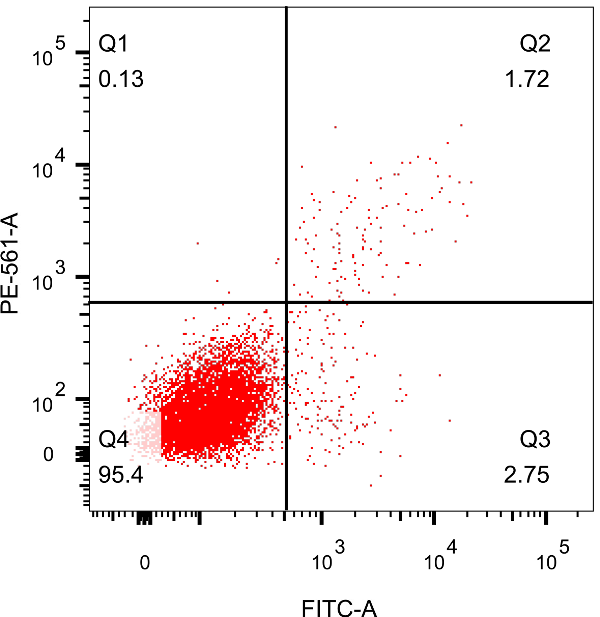

Supplement: Supplementary Figure 1 — The level of ELFN1-AS1 is upregulated in SARC tissues. [file DataSheet_1.zip › Figure 2/F/MG63/pcDNA3.1-ELFN1-AS1/2.tif]

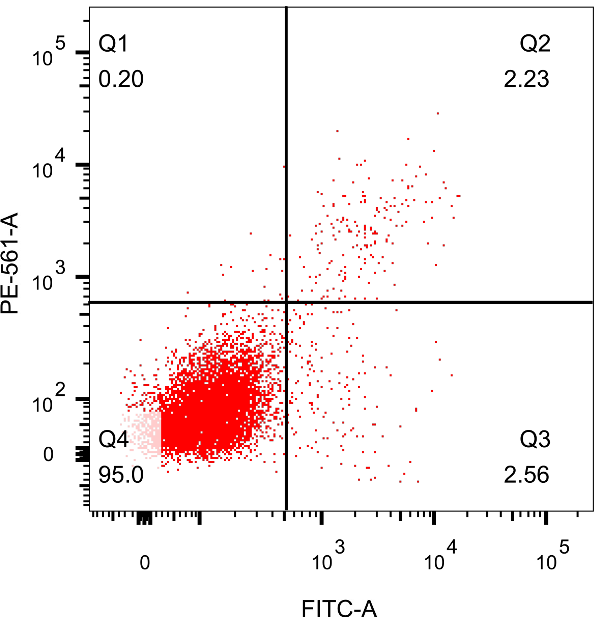

Supplement: Supplementary Figure 1 — The level of ELFN1-AS1 is upregulated in SARC tissues. [file DataSheet_1.zip › Figure 2/F/MG63/pcDNA3.1-ELFN1-AS1/3.tif]

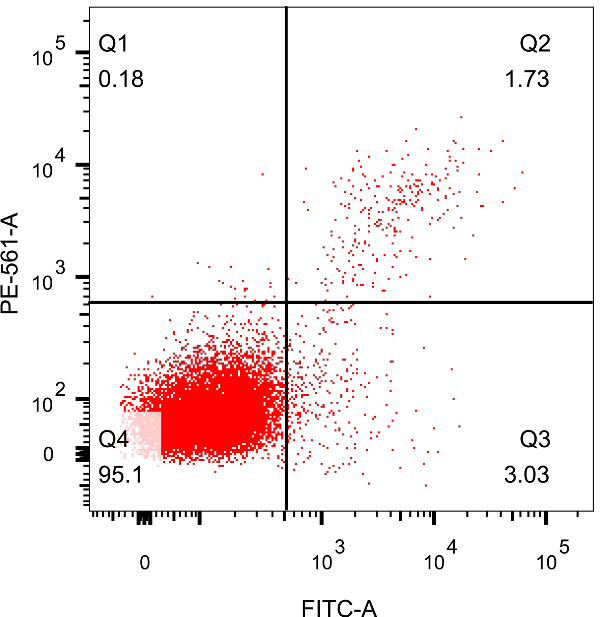

Supplement: Supplementary Figure 1 — The level of ELFN1-AS1 is upregulated in SARC tissues. [file DataSheet_1.zip › Figure 2/F/MG63/si-NC/1.tif]

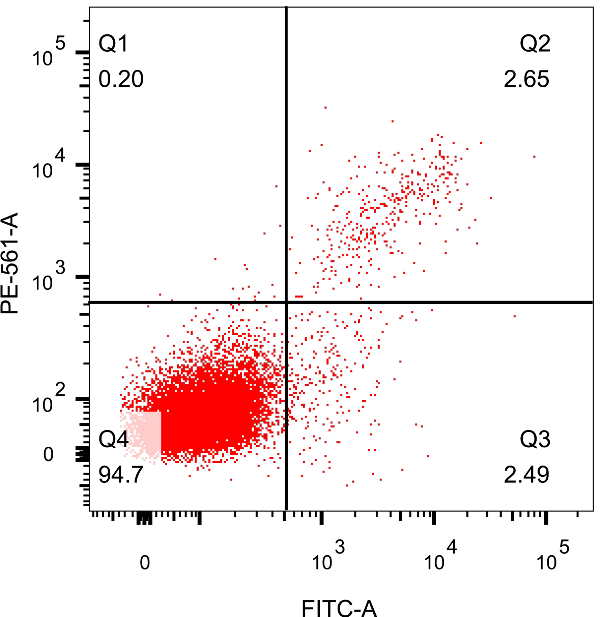

Supplement: Supplementary Figure 1 — The level of ELFN1-AS1 is upregulated in SARC tissues. [file DataSheet_1.zip › Figure 2/F/MG63/si-NC/2.tif]

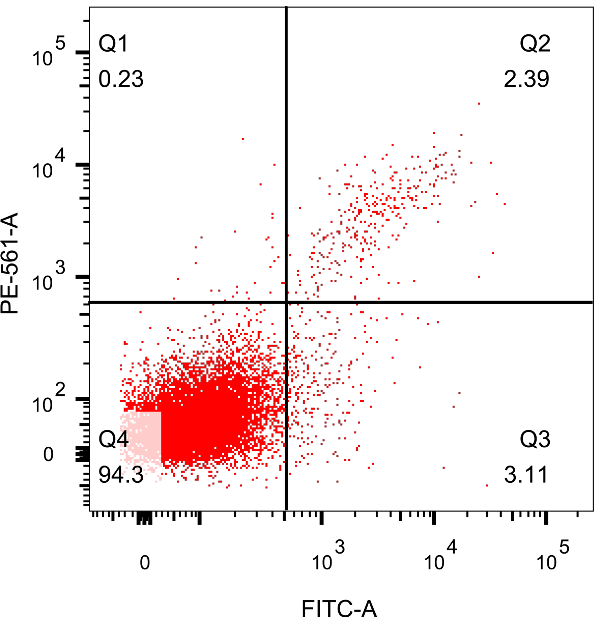

Supplement: Supplementary Figure 1 — The level of ELFN1-AS1 is upregulated in SARC tissues. [file DataSheet_1.zip › Figure 2/F/MG63/si-NC/3.tif]

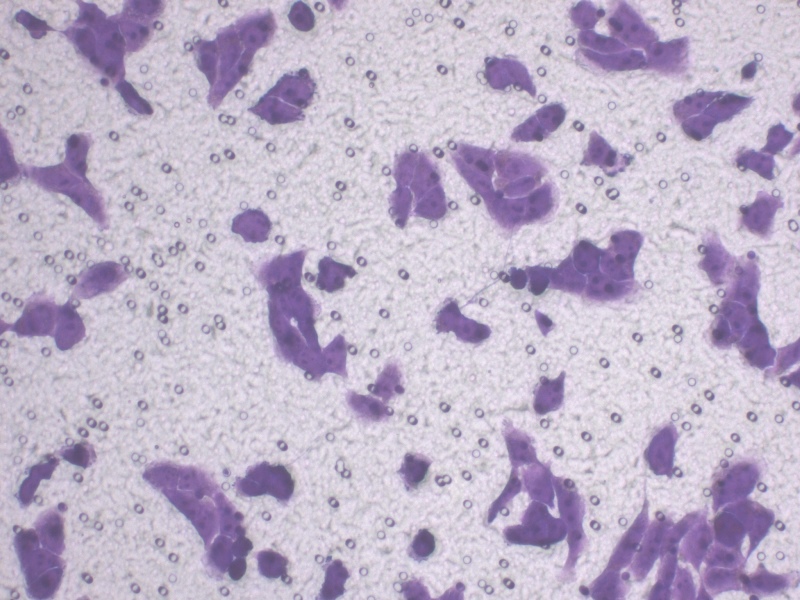

Supplement: Supplementary Figure 1 — The level of ELFN1-AS1 is upregulated in SARC tissues. [file DataSheet_1.zip › Figure 2/G/143B migration/S1/1.jpg]

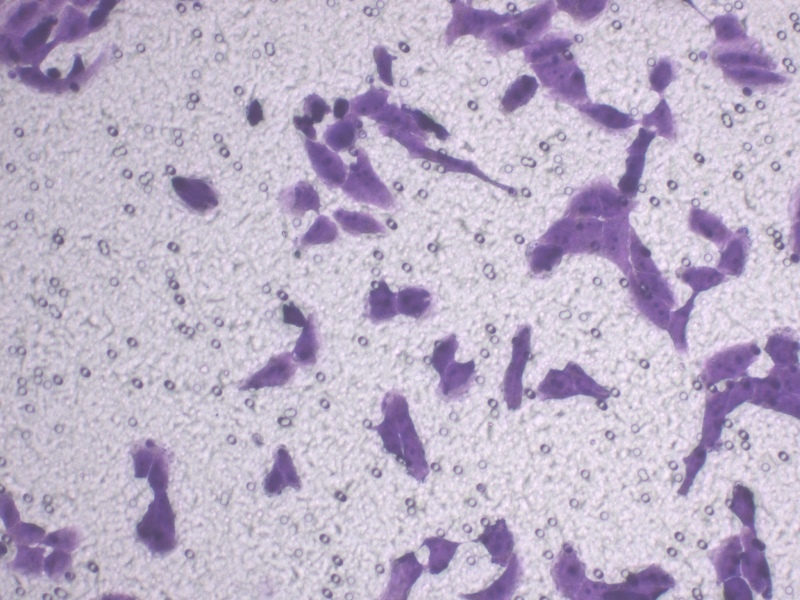

Supplement: Supplementary Figure 1 — The level of ELFN1-AS1 is upregulated in SARC tissues. [file DataSheet_1.zip › Figure 2/G/143B migration/S1/2.jpg]

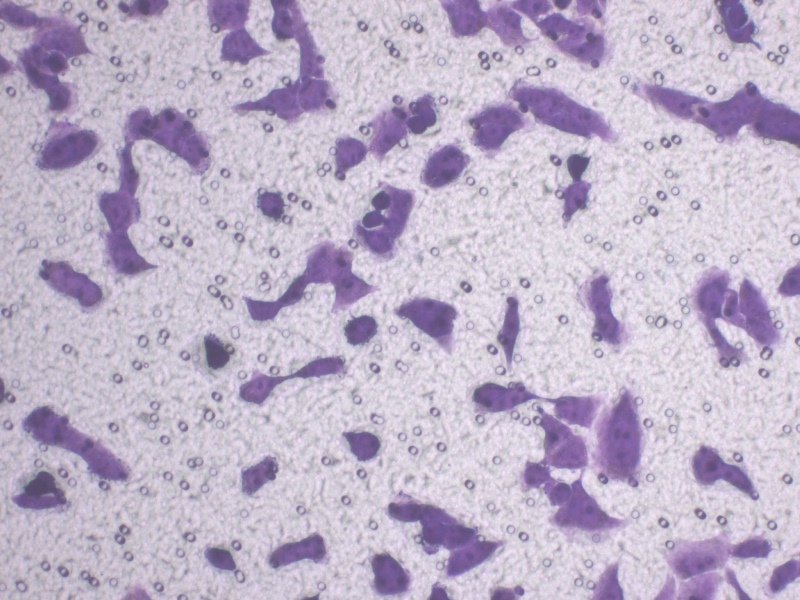

Supplement: Supplementary Figure 1 — The level of ELFN1-AS1 is upregulated in SARC tissues. [file DataSheet_1.zip › Figure 2/G/143B migration/S1/3.jpg]

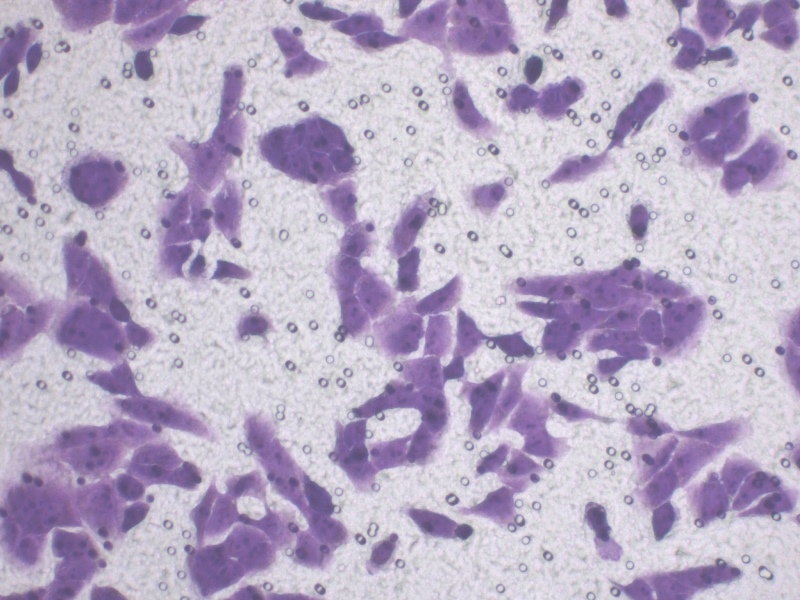

Supplement: Supplementary Figure 1 — The level of ELFN1-AS1 is upregulated in SARC tissues. [file DataSheet_1.zip › Figure 2/G/143B migration/T1/1.jpg]

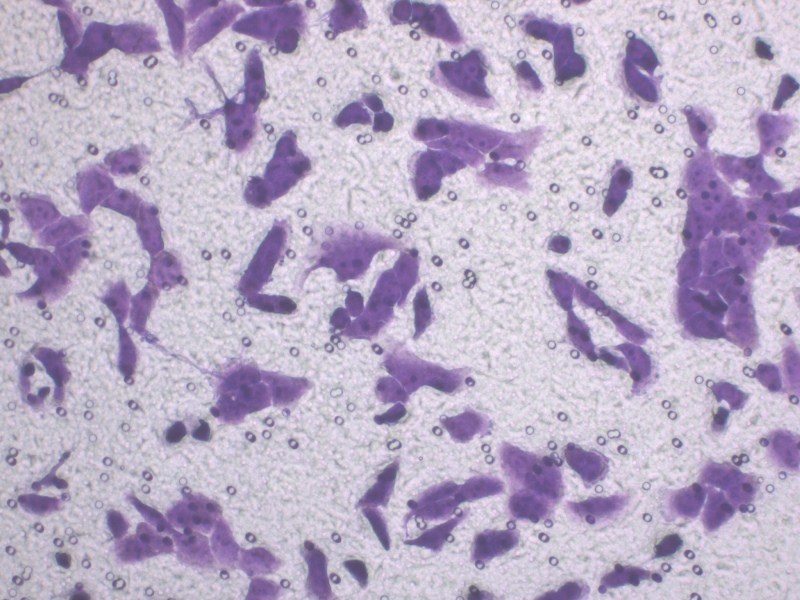

Supplement: Supplementary Figure 1 — The level of ELFN1-AS1 is upregulated in SARC tissues. [file DataSheet_1.zip › Figure 2/G/143B migration/T1/2.jpg]

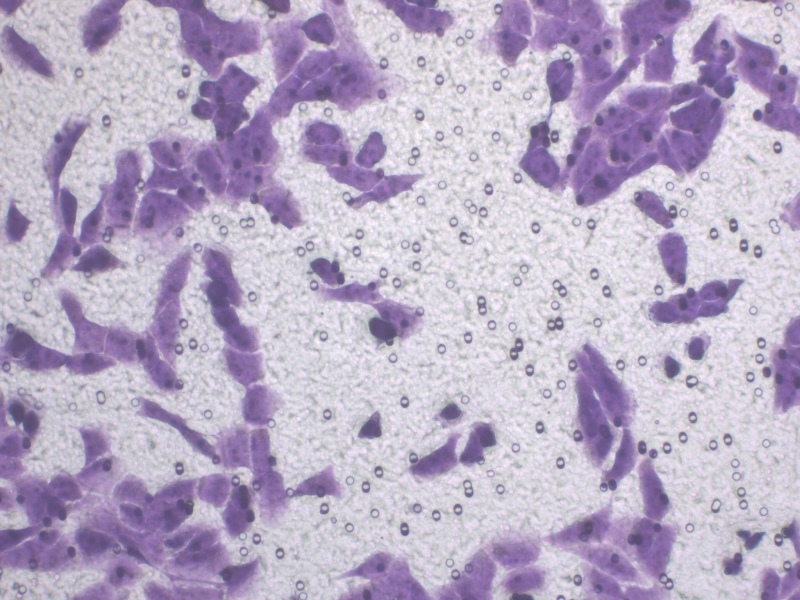

Supplement: Supplementary Figure 1 — The level of ELFN1-AS1 is upregulated in SARC tissues. [file DataSheet_1.zip › Figure 2/G/143B migration/T1/3.jpg]

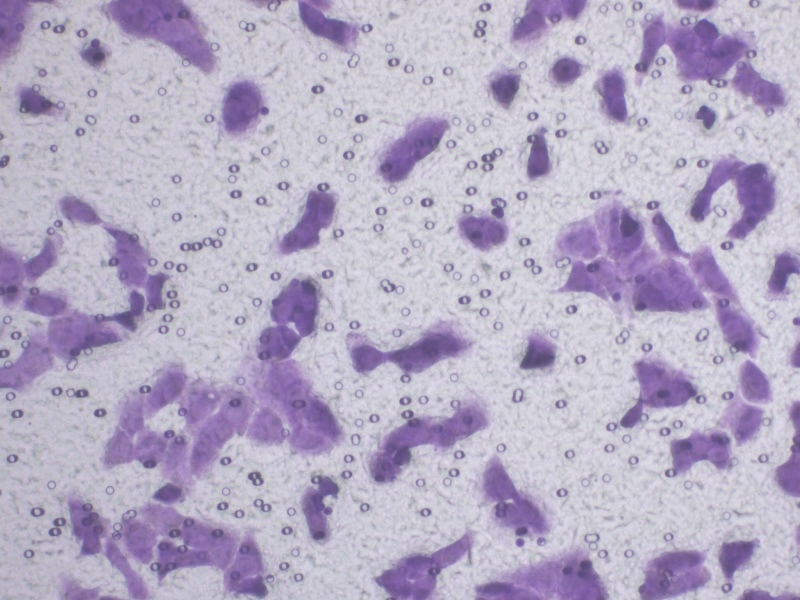

Supplement: Supplementary Figure 1 — The level of ELFN1-AS1 is upregulated in SARC tissues. [file DataSheet_1.zip › Figure 2/G/143B migration/U1/1.jpg]

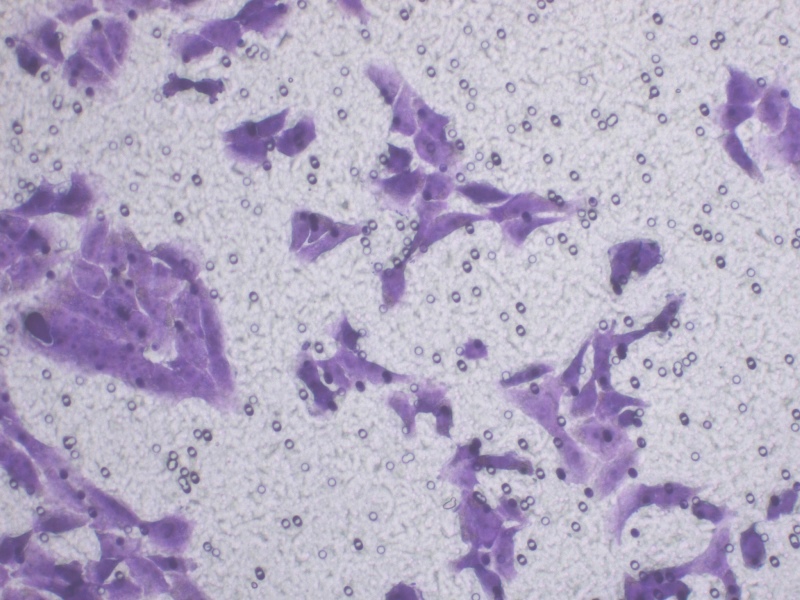

Supplement: Supplementary Figure 1 — The level of ELFN1-AS1 is upregulated in SARC tissues. [file DataSheet_1.zip › Figure 2/G/143B migration/U1/2.jpg]

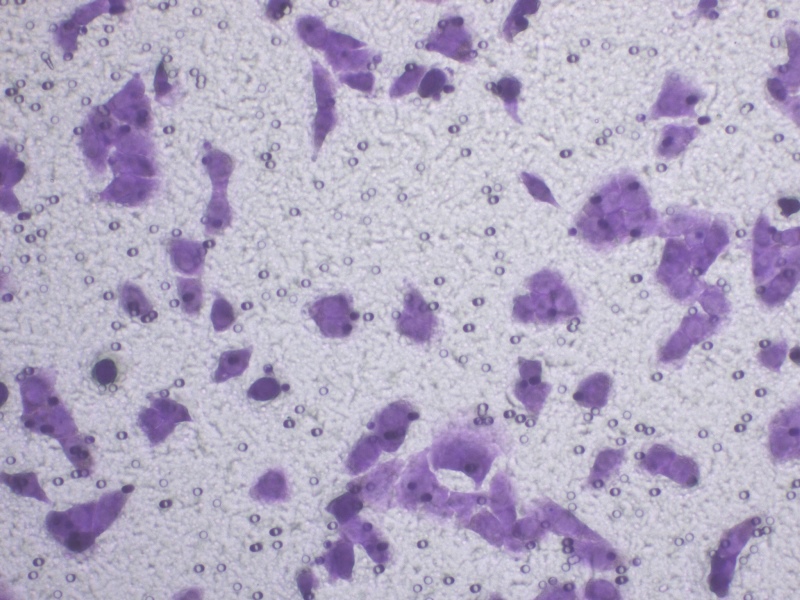

Supplement: Supplementary Figure 1 — The level of ELFN1-AS1 is upregulated in SARC tissues. [file DataSheet_1.zip › Figure 2/G/143B migration/U1/3.jpg]

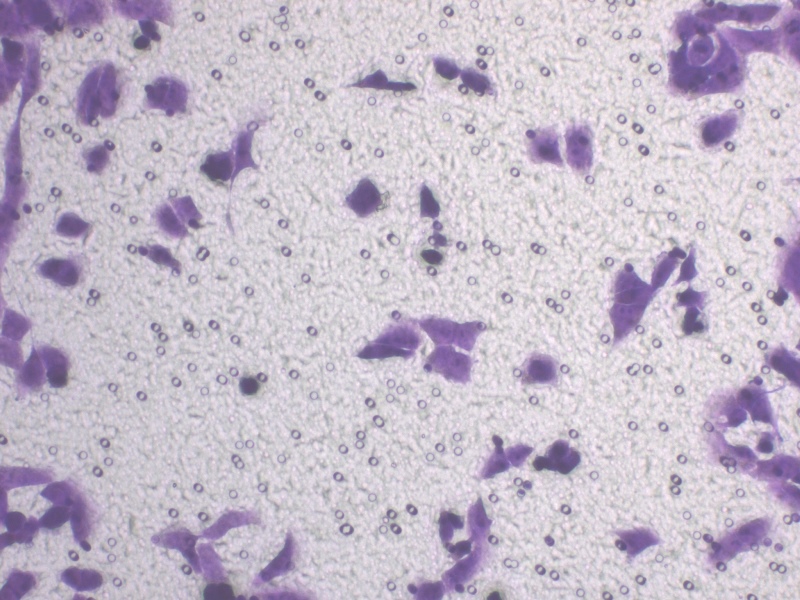

Supplement: Supplementary Figure 1 — The level of ELFN1-AS1 is upregulated in SARC tissues. [file DataSheet_1.zip › Figure 2/G/143B migration/V1/1.jpg]

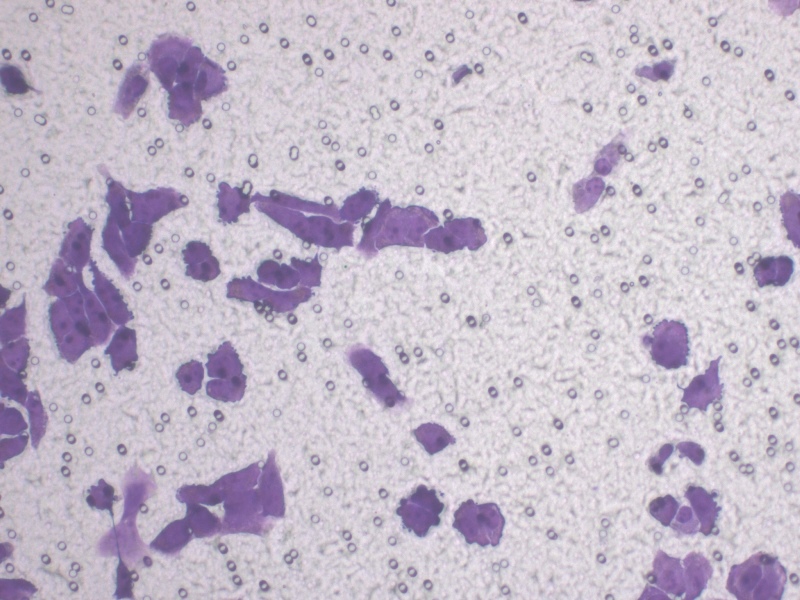

Supplement: Supplementary Figure 1 — The level of ELFN1-AS1 is upregulated in SARC tissues. [file DataSheet_1.zip › Figure 2/G/143B migration/V1/2.jpg]

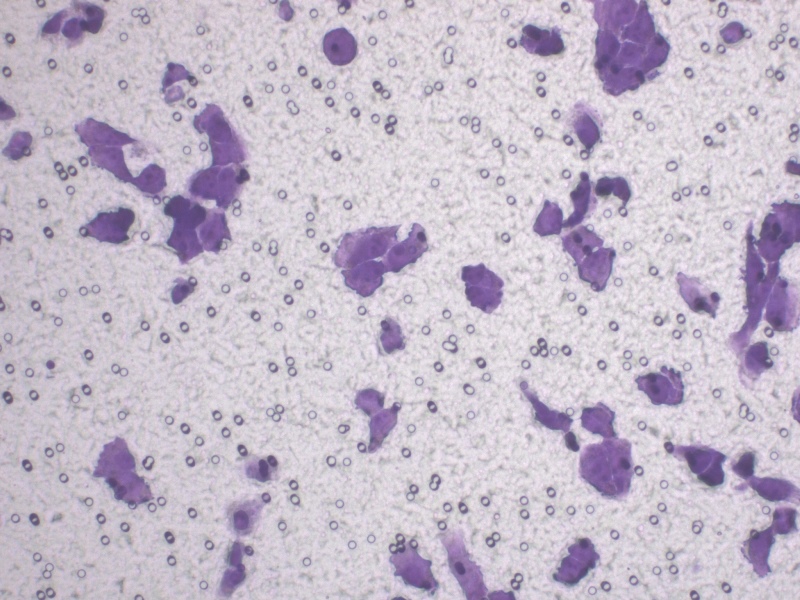

Supplement: Supplementary Figure 1 — The level of ELFN1-AS1 is upregulated in SARC tissues. [file DataSheet_1.zip › Figure 2/G/143B migration/V1/3.jpg]

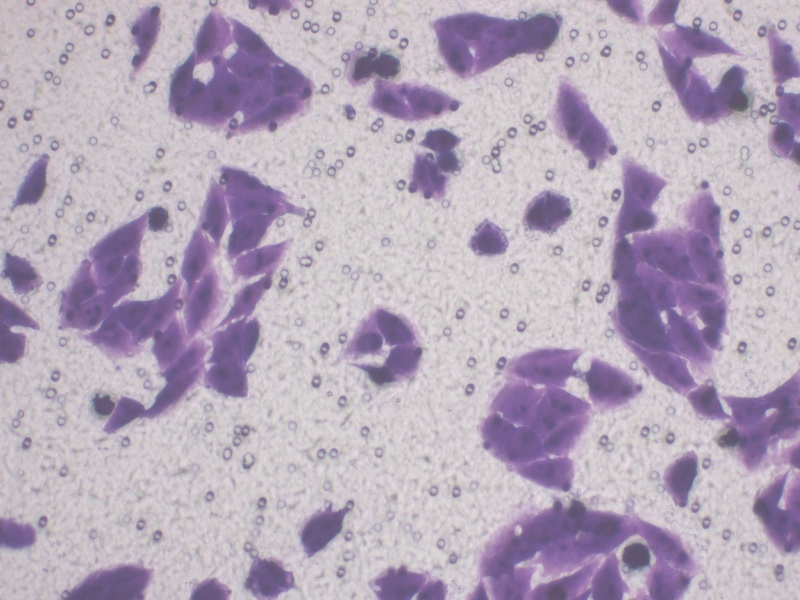

Supplement: Supplementary Figure 1 — The level of ELFN1-AS1 is upregulated in SARC tissues. [file DataSheet_1.zip › Figure 2/G/MG63 migration/W1/1.jpg]

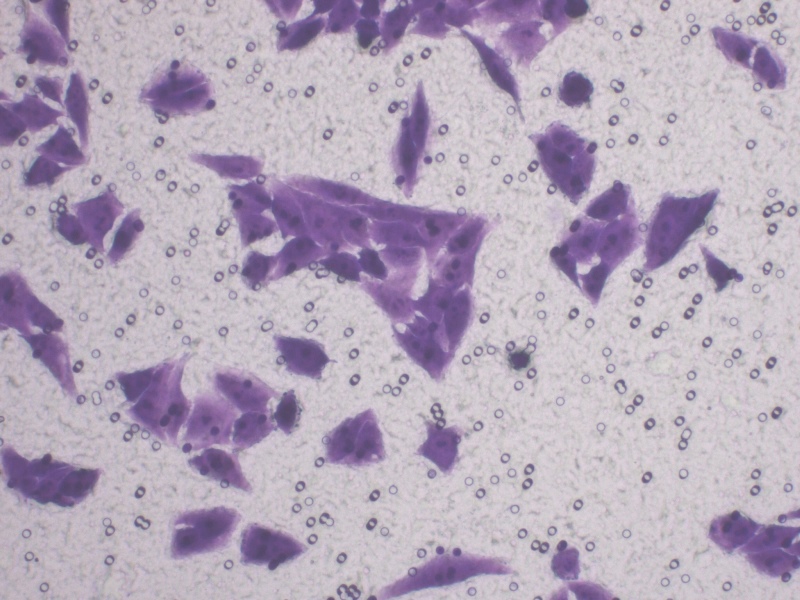

Supplement: Supplementary Figure 1 — The level of ELFN1-AS1 is upregulated in SARC tissues. [file DataSheet_1.zip › Figure 2/G/MG63 migration/W1/2.jpg]

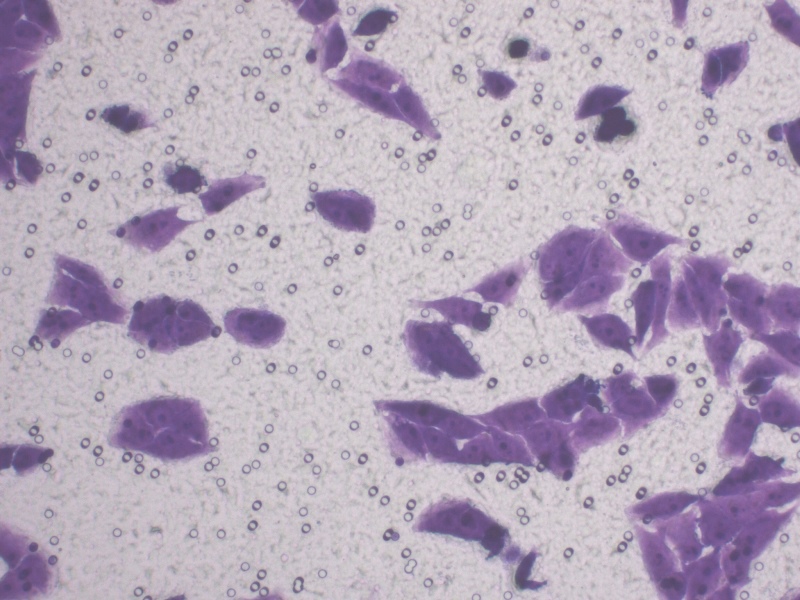

Supplement: Supplementary Figure 1 — The level of ELFN1-AS1 is upregulated in SARC tissues. [file DataSheet_1.zip › Figure 2/G/MG63 migration/W1/3.jpg]

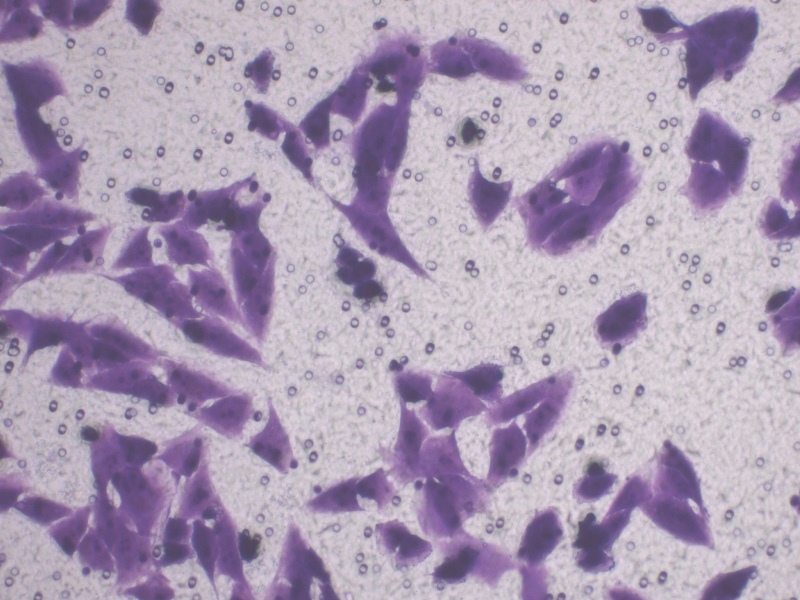

Supplement: Supplementary Figure 1 — The level of ELFN1-AS1 is upregulated in SARC tissues. [file DataSheet_1.zip › Figure 2/G/MG63 migration/X1/1.jpg]

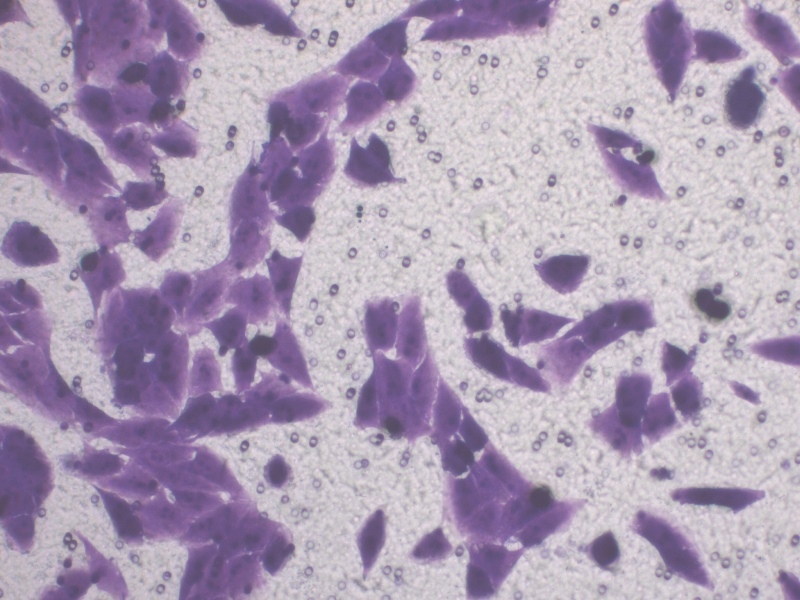

Supplement: Supplementary Figure 1 — The level of ELFN1-AS1 is upregulated in SARC tissues. [file DataSheet_1.zip › Figure 2/G/MG63 migration/X1/2.jpg]

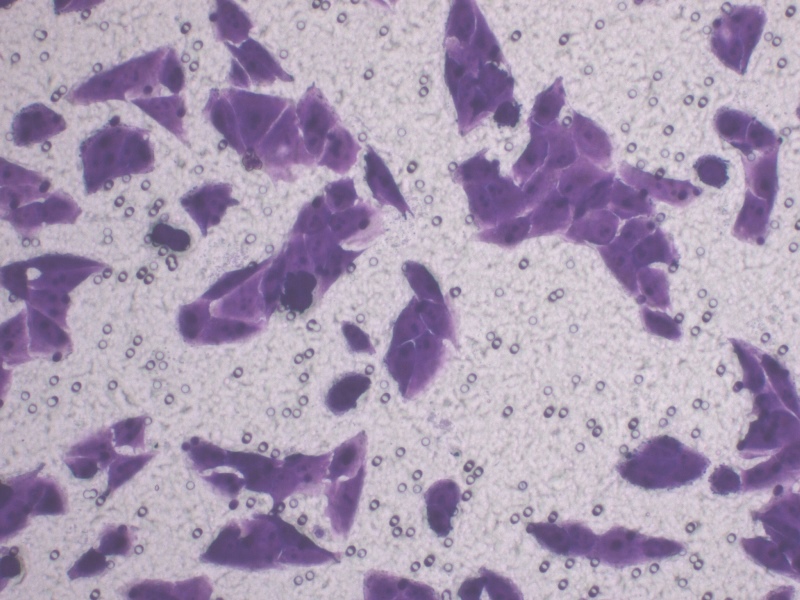

Supplement: Supplementary Figure 1 — The level of ELFN1-AS1 is upregulated in SARC tissues. [file DataSheet_1.zip › Figure 2/G/MG63 migration/X1/3.jpg]

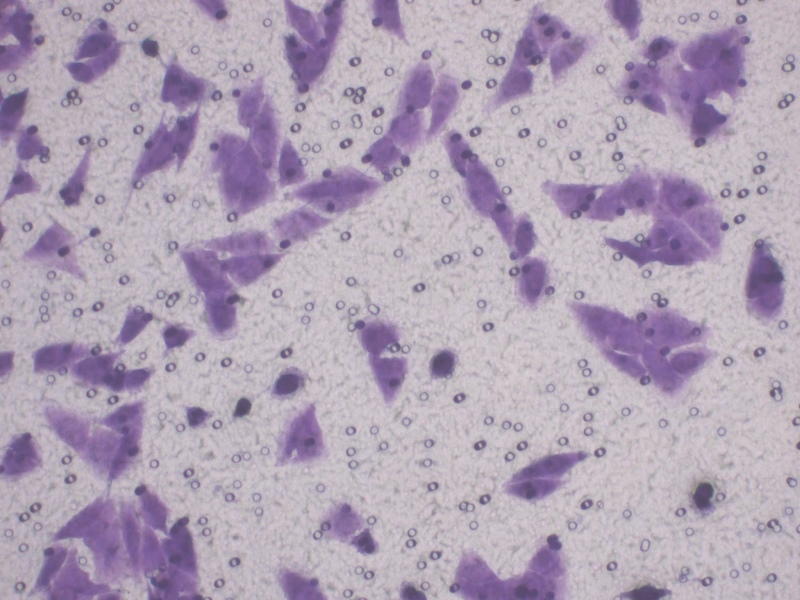

Supplement: Supplementary Figure 1 — The level of ELFN1-AS1 is upregulated in SARC tissues. [file DataSheet_1.zip › Figure 2/G/MG63 migration/Y1/1.jpg]

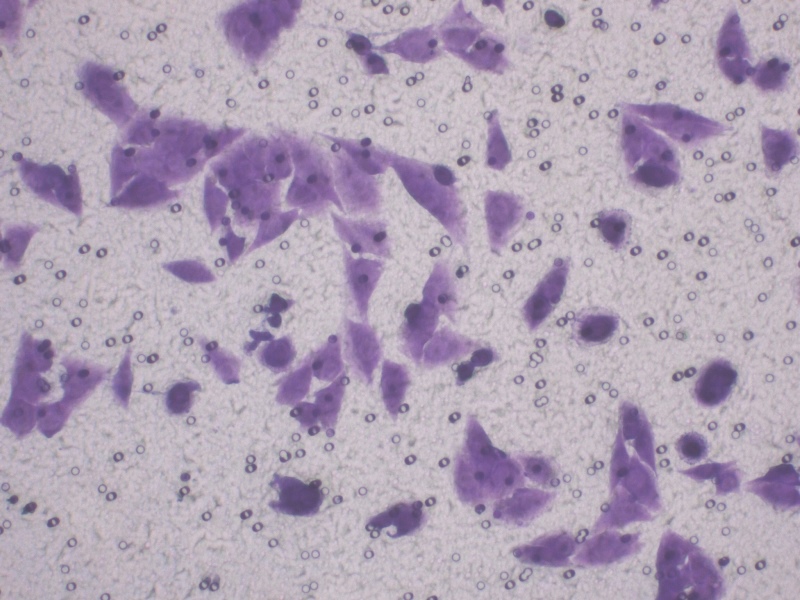

Supplement: Supplementary Figure 1 — The level of ELFN1-AS1 is upregulated in SARC tissues. [file DataSheet_1.zip › Figure 2/G/MG63 migration/Y1/2.jpg]

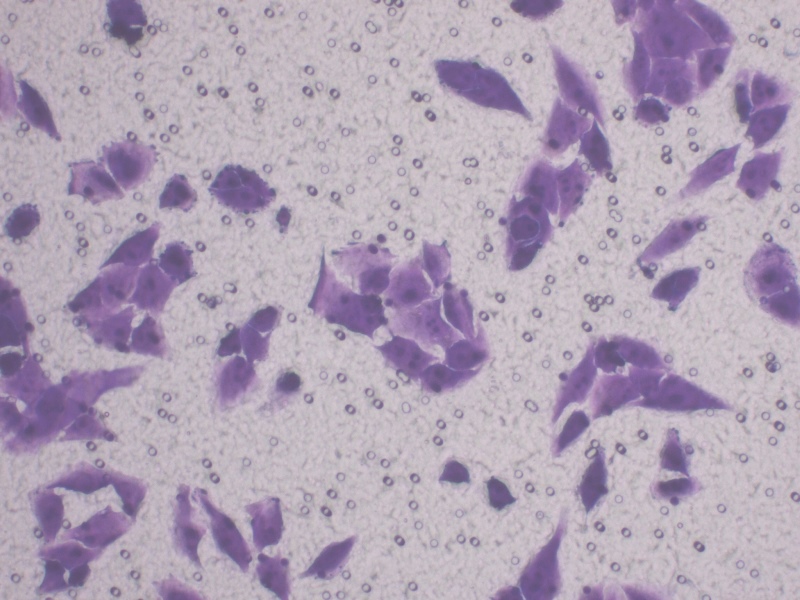

Supplement: Supplementary Figure 1 — The level of ELFN1-AS1 is upregulated in SARC tissues. [file DataSheet_1.zip › Figure 2/G/MG63 migration/Y1/3.jpg]

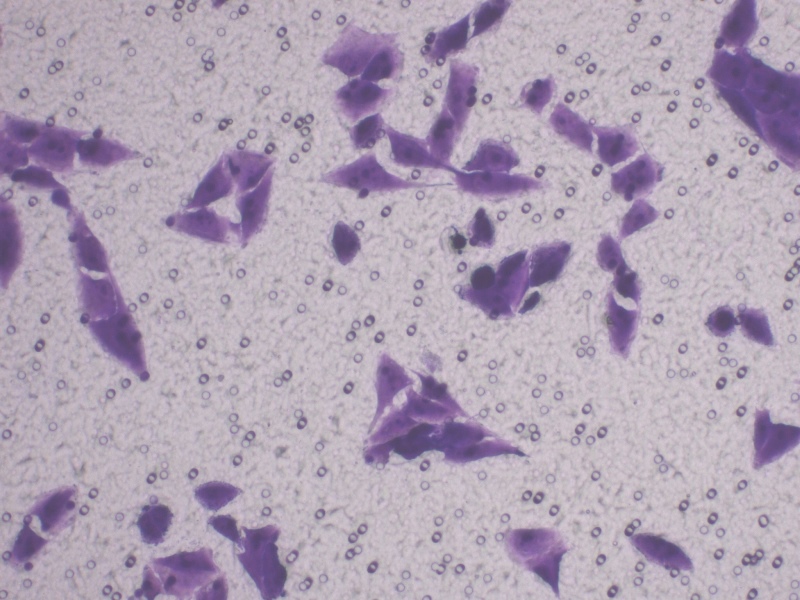

Supplement: Supplementary Figure 1 — The level of ELFN1-AS1 is upregulated in SARC tissues. [file DataSheet_1.zip › Figure 2/G/MG63 migration/Z1/1.jpg]

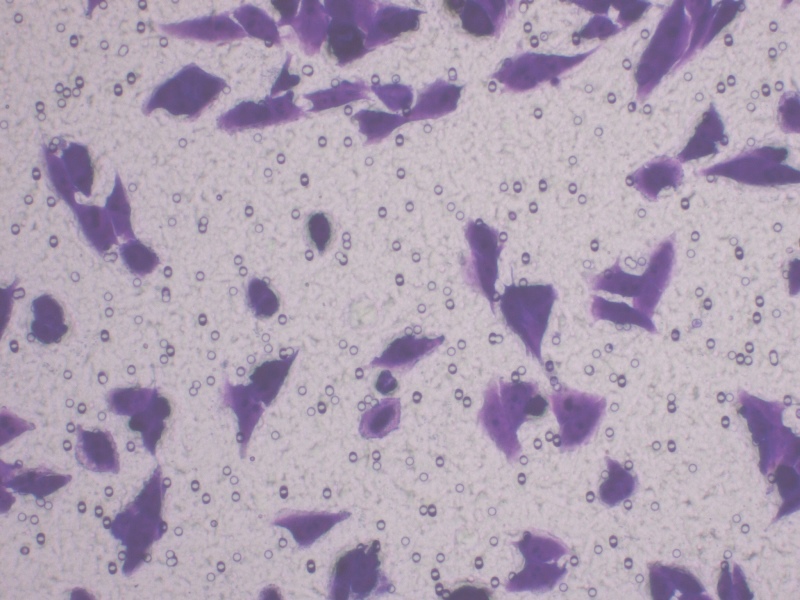

Supplement: Supplementary Figure 1 — The level of ELFN1-AS1 is upregulated in SARC tissues. [file DataSheet_1.zip › Figure 2/G/MG63 migration/Z1/2.jpg]

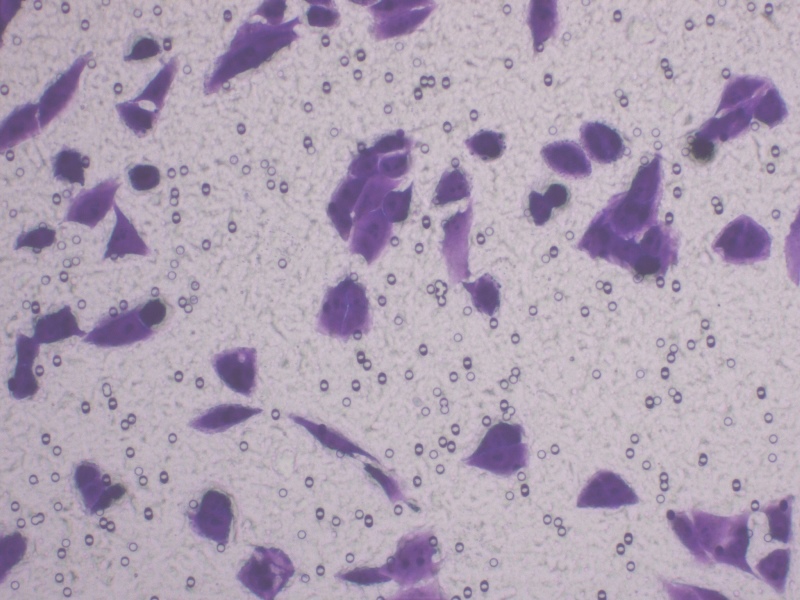

Supplement: Supplementary Figure 1 — The level of ELFN1-AS1 is upregulated in SARC tissues. [file DataSheet_1.zip › Figure 2/G/MG63 migration/Z1/3.jpg]

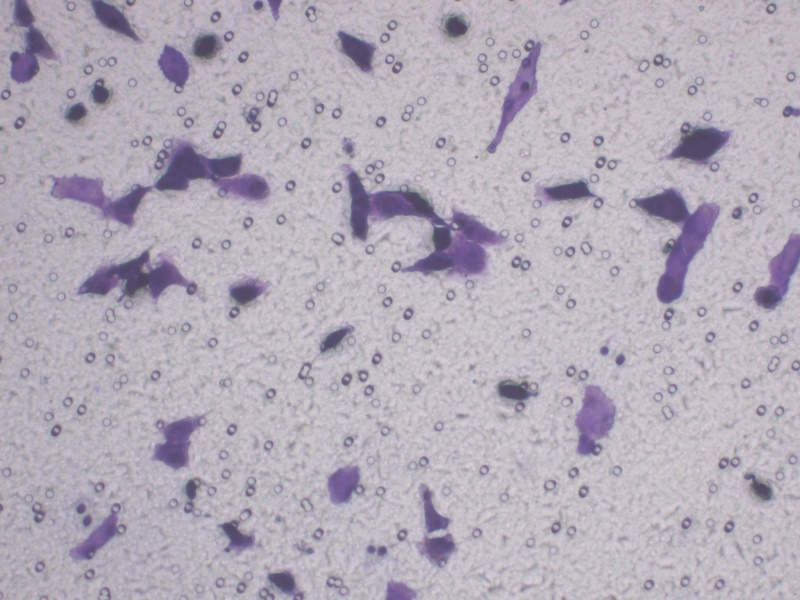

Supplement: Supplementary Figure 1 — The level of ELFN1-AS1 is upregulated in SARC tissues. [file DataSheet_1.zip › Figure 2/H/143B invasion/S1/1.jpg]

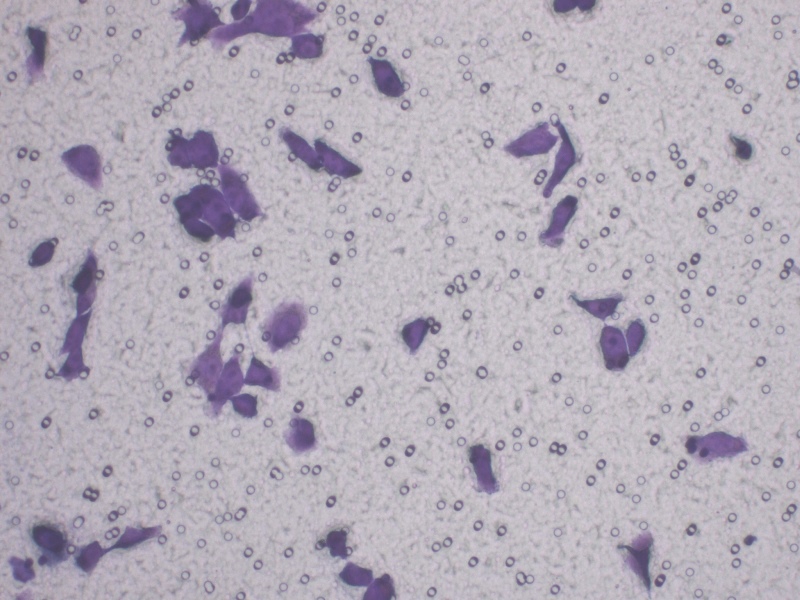

Supplement: Supplementary Figure 1 — The level of ELFN1-AS1 is upregulated in SARC tissues. [file DataSheet_1.zip › Figure 2/H/143B invasion/S1/2.jpg]

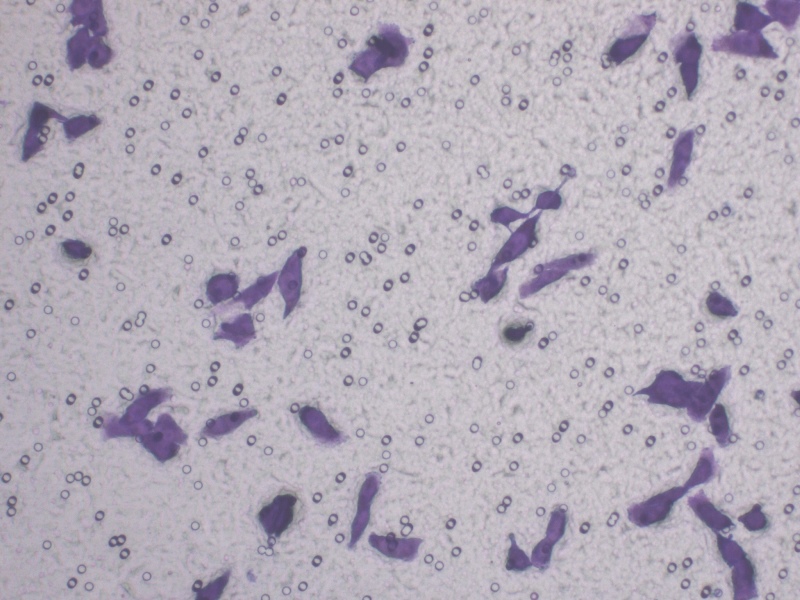

Supplement: Supplementary Figure 1 — The level of ELFN1-AS1 is upregulated in SARC tissues. [file DataSheet_1.zip › Figure 2/H/143B invasion/S1/3.jpg]

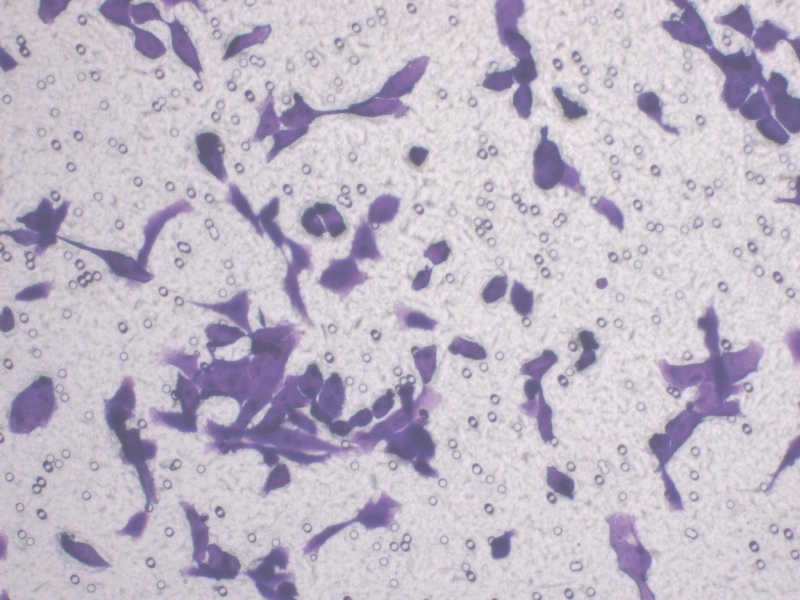

Supplement: Supplementary Figure 1 — The level of ELFN1-AS1 is upregulated in SARC tissues. [file DataSheet_1.zip › Figure 2/H/143B invasion/T1/1.jpg]

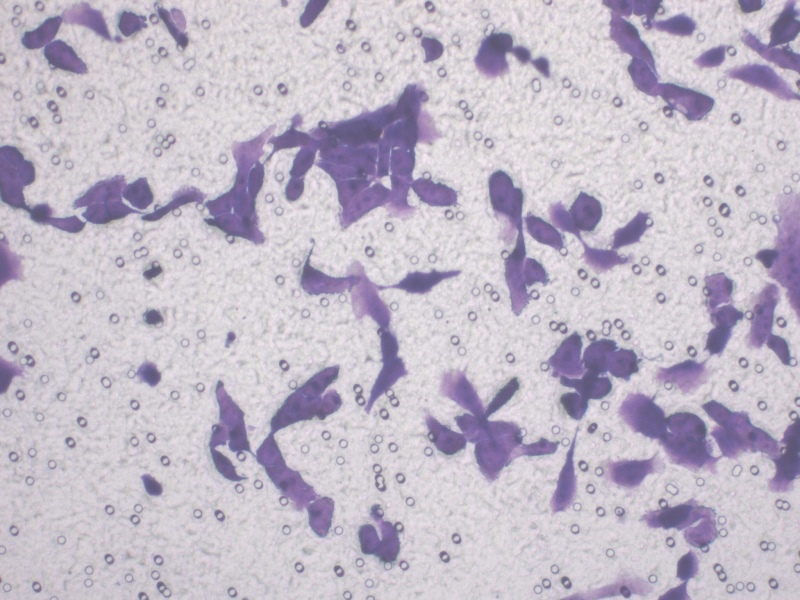

Supplement: Supplementary Figure 1 — The level of ELFN1-AS1 is upregulated in SARC tissues. [file DataSheet_1.zip › Figure 2/H/143B invasion/T1/2.jpg]

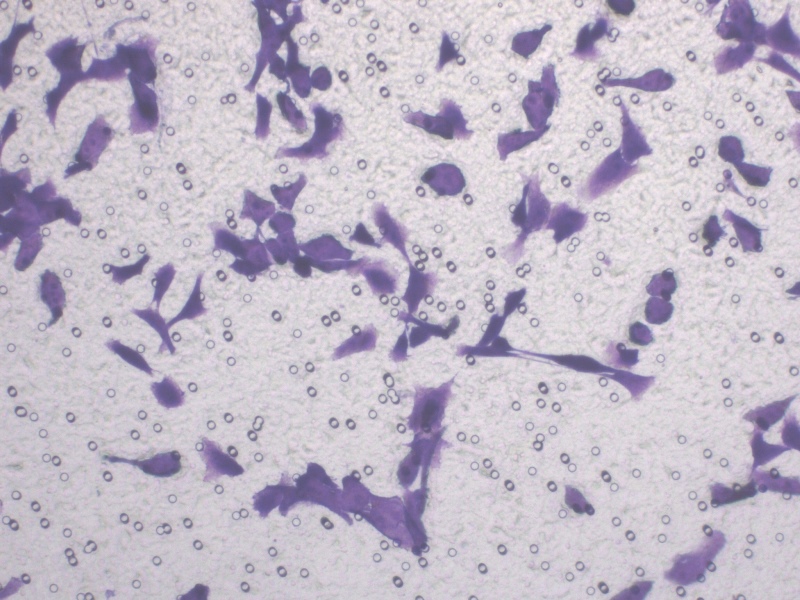

Supplement: Supplementary Figure 1 — The level of ELFN1-AS1 is upregulated in SARC tissues. [file DataSheet_1.zip › Figure 2/H/143B invasion/T1/3.jpg]

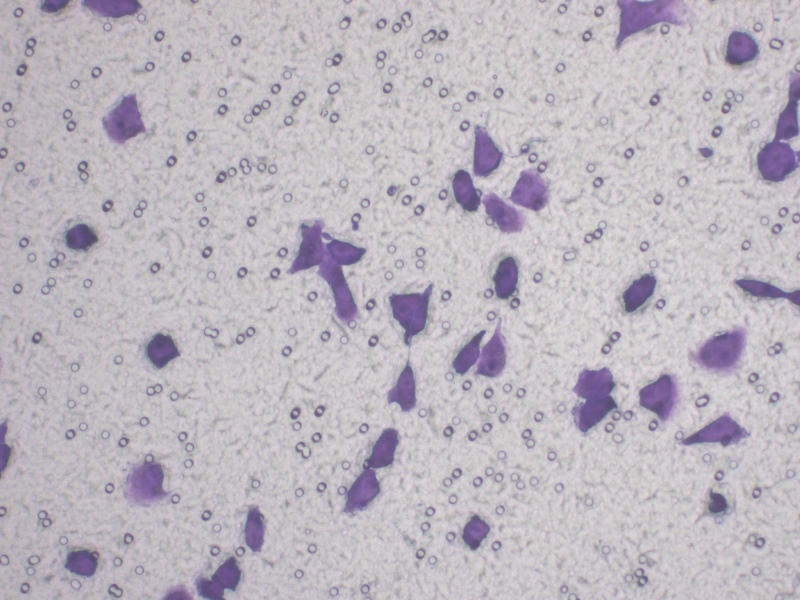

Supplement: Supplementary Figure 1 — The level of ELFN1-AS1 is upregulated in SARC tissues. [file DataSheet_1.zip › Figure 2/H/143B invasion/U1/1.jpg]

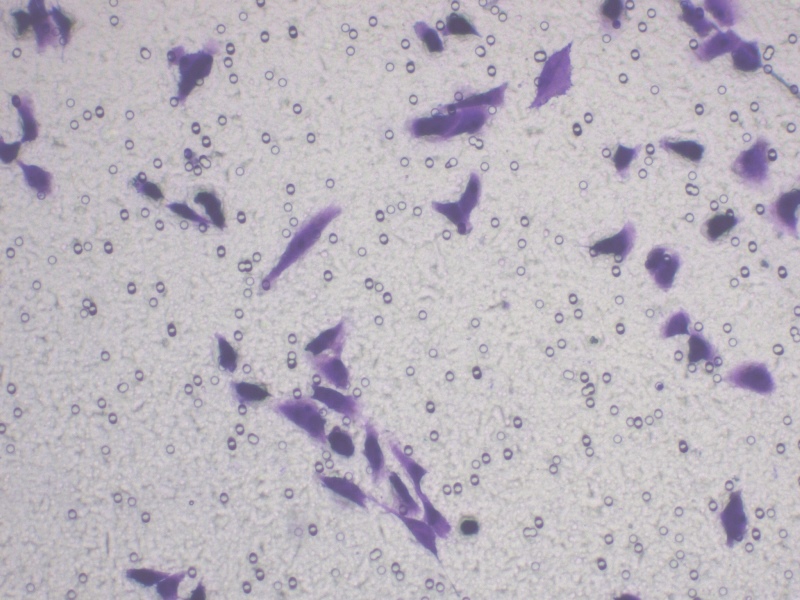

Supplement: Supplementary Figure 1 — The level of ELFN1-AS1 is upregulated in SARC tissues. [file DataSheet_1.zip › Figure 2/H/143B invasion/U1/2.jpg]

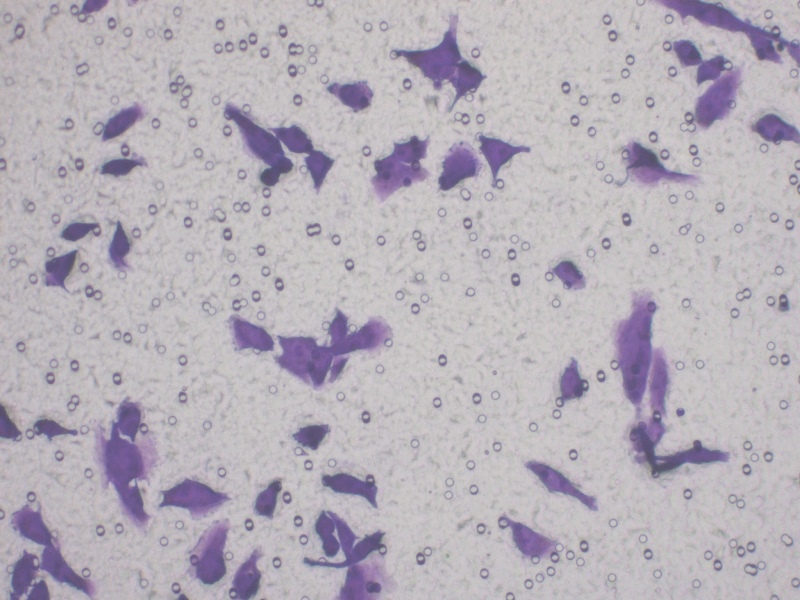

Supplement: Supplementary Figure 1 — The level of ELFN1-AS1 is upregulated in SARC tissues. [file DataSheet_1.zip › Figure 2/H/143B invasion/U1/3.jpg]

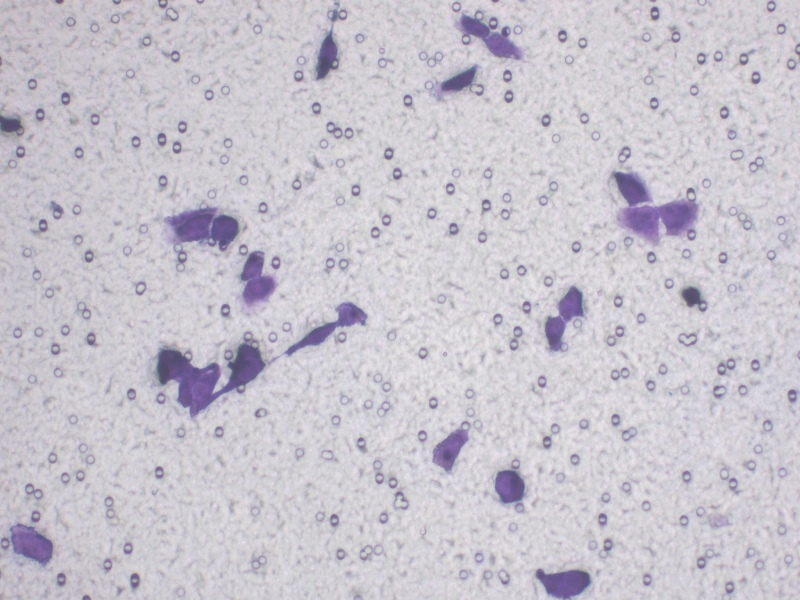

Supplement: Supplementary Figure 1 — The level of ELFN1-AS1 is upregulated in SARC tissues. [file DataSheet_1.zip › Figure 2/H/143B invasion/V1/1.jpg]

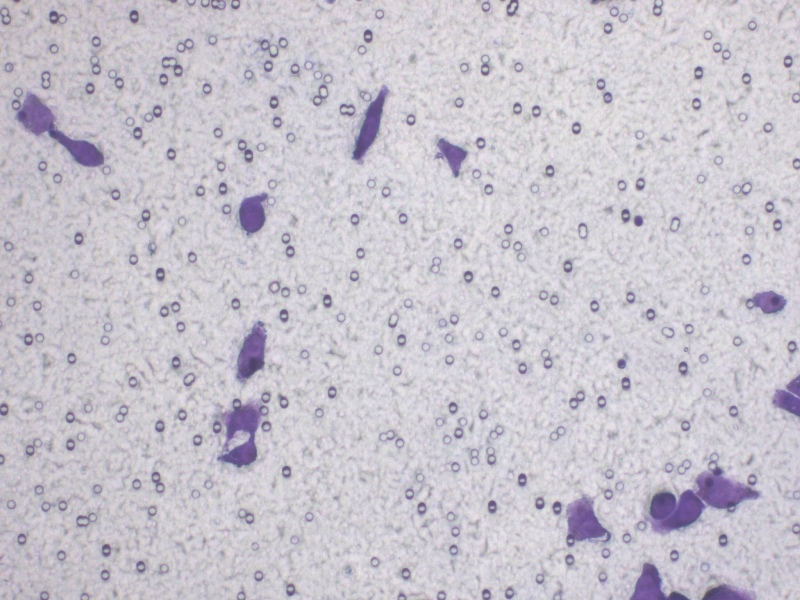

Supplement: Supplementary Figure 1 — The level of ELFN1-AS1 is upregulated in SARC tissues. [file DataSheet_1.zip › Figure 2/H/143B invasion/V1/2.jpg]

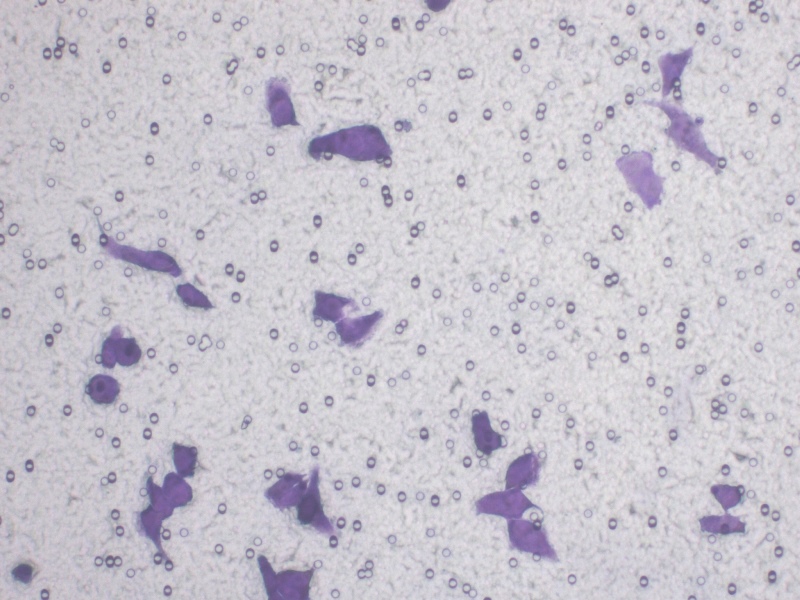

Supplement: Supplementary Figure 1 — The level of ELFN1-AS1 is upregulated in SARC tissues. [file DataSheet_1.zip › Figure 2/H/143B invasion/V1/3.jpg]

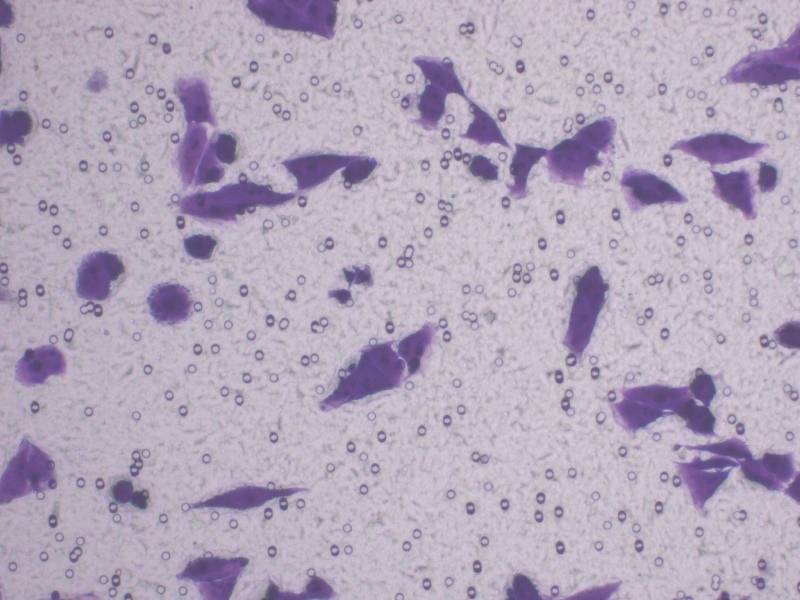

Supplement: Supplementary Figure 1 — The level of ELFN1-AS1 is upregulated in SARC tissues. [file DataSheet_1.zip › Figure 2/H/MG63 invasion/W1/1.jpg]

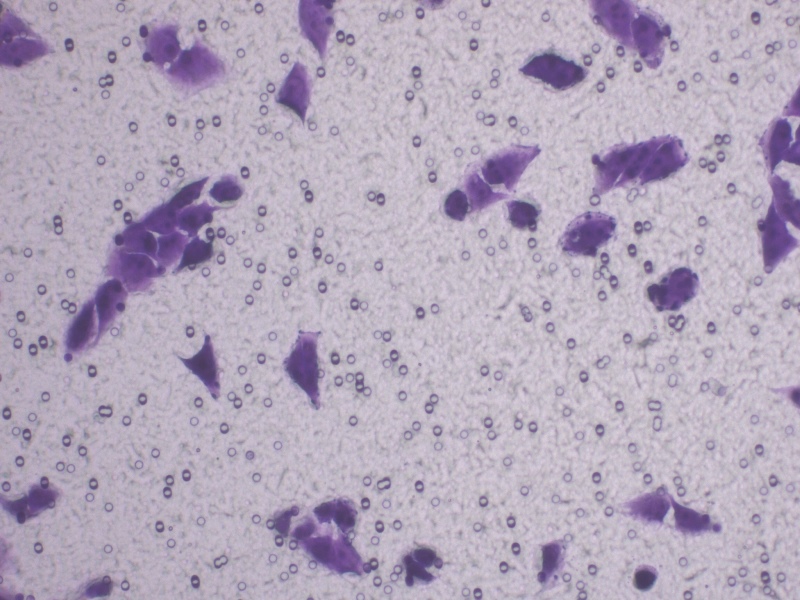

Supplement: Supplementary Figure 1 — The level of ELFN1-AS1 is upregulated in SARC tissues. [file DataSheet_1.zip › Figure 2/H/MG63 invasion/W1/2.jpg]

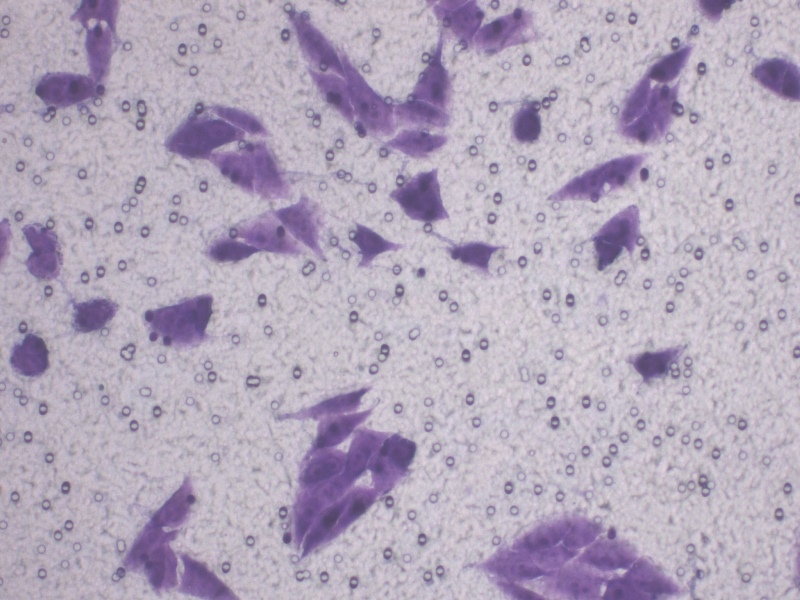

Supplement: Supplementary Figure 1 — The level of ELFN1-AS1 is upregulated in SARC tissues. [file DataSheet_1.zip › Figure 2/H/MG63 invasion/W1/3.jpg]
